# Pancreatic surgery outcomes: multicentre prospective snapshot study in 67 countries

**DOI:** 10.1093/bjs/znad330

**Published:** 2023-11-09

**Authors:** PancreasGroup.org Collaborative, Giuseppe Kito Fusai, Dimitri Aristotle Raptis, Mohamed Abu Hilal, Claudio Bassi, Marc Besselink, Kevin Conlon, Brian Davidson, Marco Del Chiaro, Christos Dervenis, Isabella Frigerio, Massimo Falconi, Thilo Hackert, Ewen M Harrison, Shailesh V Shrikhande, Ajith Siriwardena, Martin Smith, Christopher Wolfgang, Aditya Borakati, Deniz Balci, Muhammed Elhadi, Camila Hidalgo Salinas, Nikolaos Machairas, Giovanni Marchegiani, Atsushi Oba, Christian Oberkofler, Ioannis Passas, Reena Ravikumar, Patricia Sánchez Velázquez, Martin de Santibañes, Andreas Anton Schnitzbauer, Fiammetta Soggiu, Domenico Tamburrino, Pascale Tinguely, Alice Wei, Marinos Zachiotis, Kamel Bentabak, Salah Eddine Kacimi, Mehrdad Nikfarjam, Aliaksei Shcherba, Gregory Sergeant, Gustavo Coelho, Orlando Torres, Nikolay Belev, Ephraim Tang, Christian Diaz, Kongyuan Wei, Maher Hendi, Nikolaos Gouvas, Thalis Christophides, Andrej Nikov, Dalia Fathallah, Mahmoud Saad, Olav Tammik, Heikki Huhta, Laurent Sulpice, Renato Lupinacci, Zaza Demetrashvili, Gregor A Stavrou, Greece Evangelos Felekouras, Vasileios Papaziogas, Sanjeev Misra, Hashim Talib, Maytham Al-Juaifari Al-Sader, Sohei Satoi, Khaled Obeidat, Ildar Fakhradiyev, Mohamad Khalife, Muhammed Elhadi, Audrius Dulskas, Shahi Ghani, Alejandro Eduardo Padilla, Javier Melchor-Ruan, Sarnai Erdene, Amine Benkabbou, Pueya Nashidengo, Jonathan Koea, Nigeria Ademola Adeyeye, Sarah Amro, Walaa Mohammed Alnammourah, Catherine The, Michał Pędziwiatr, Wojciech Polkowski, Sorin Traian Barbu, Daniel Galun, Brian K P Goh, Blaž Trotovšek, Jones Omoshoro-Jones, Benedetto Ielpo, Abdelfatah Abdelmageed, Per Sandström, Alessandra Cristaudi, Beat Gloor, Christoph Kuemmerli, Alaa Hamdan Tishreen, Mohammad Karam Chaaban, Chien Hui Wu, Po-Chih Yang Fu Jen, Oussama Baraket, Mark Taylor, Nigel Jamieson, Satheesh Iype, Emmanouil Giorgakis, Motaz Qadan, Sabha Ganai, Hamza Al-Naggar, Onesai Chihaka, Amor El Behi, Aya Tinhinane Kouicem, Aissam Chibane, Chafik Bouzid, Kamel Bentabak, Imene Bouali, Noureddine Samai, Boudouh Aya, Boutheyna Drid, Anisse Tidjane, Benali Tabeti, Nabil Boudjenan-Serradj, Mohammed Hakim Larbi, Ilhem Ouahab, Souhem Touabti, Ouahab Ilhem, Souad Bouaoud, Abdoun Meriem, Amel Ouyahia, Aya Tinhinane Kouicem, Meriem Abdoun, Rais Mounira, Mounira Rais, Omar Riffi, Salah Eddine Kacimi, Lucas McCormack, Pablo Capitanich, Jeremias Goransky, Martin de Santibanes, Oscar Mazza, Ivana Pedraza Salazar, Dario Roberto Ramallo, Farinelli Pablo, Gabriel E Gondolesi, Pablo Barros Schelotto, Jorge Rodriguez, Christos Apostolou, Neil Merrett, Adrian Fox, Sayed Hassen, Shantanu Joglekar, Sivakumar Gananadha, Rudyard Wake, Krista Hagen, Mithra Sritharan, Kat Hall, Vijayaragavan Muralidharan, Kai Brown, Mehrdad Nikfarjam, Daniel Croagh, Mithra Sritharan, Roger Berry, Aly Fayed, Russell Hodgson, Thiep Kuany, Benjamin Loveday, Samuel Banting, Alistair Rowcroft, Adrian Fox, Brett Knowles, Lillian Taylor, Lynn Chong, Simon Banting, Marcos Perini, Mehrdad Nikfarjam, Yi-Ju Lin, Ali Alsoudani, David Burnett, Kalpesh Shah, Matthew Fuge, Nicholas Bull, Stanley Chen, Suresh Navadgi, Zi Qin Ng, Mikael Johansson, Nur Sabrina Binti Babe Azaman, Andrew Pearson, Christos Apostolou, Hans Mischinger, Peter Schemmer, Peter Kornprat, Andreas Hauer, Andreas Hauer, Klaus Kirbes, Reinhold Klug, Rudolf Schrittwieser, Alexander Klaus, Alexandra Entschev, Daniel Reichhold, Keti Ugrekhelidze, Marcus Fink, Radoslava Stoyanova, Marah Sabateen, Zainab Mahfoodh, Hamdi Al Shenawi, Rami Yaghan, Mohammad Chowdhury, Aliaksei Shcherba, Leonid Kirkovsky, Sergey Korotkov, Bert Van den Bossche, Kim Boterbergh, Martin Poortmans, Bart Smet, Sébastien Strypstein, Tom Feryn, El Mahdi Wahib, sara oubella, Geert Roeyen, Vera Hartman, Bart Bracke, Bart Hendrikx, Filip Gryspeerdt, Frederik Berrevoet, Natalie Poortmans, Thomas Apers, Bart Appeltans, Bart Appeltans, Dennis Wicherts, Gregory Sergeant, Fernanda Oliveira Barreto Garcia, Ian Barroso dos Santos, Rafael Garcia, Rinaldo Pinto, Thais Lins Soares Leite, Marciano Anghinoni, Caroline Celestino Girão Nobre, Gustavo Coelho, Ivens Filizola Soares Machado, Nubyhelia Carvalho, Lucio Morais, Aldo Vieira Barros, Gustavo Gomes, Igor Lima Buarque, Alessandro Bersch Osvaldt, Matheus Militz, Marcio Boff, Luciano Marcelino, Enilde Guerra, Lucas Torelly, Fábio Luiz Waechter, Pablo Rodrigues, Uirá Fernandes Teixeira, Alessandro Osvaldt, Luciano Marcelino, Matheus Militz, Eduardo De Mello, Rinaldo Goncalves, Silvio Balzan, Eduardo Jose B Ramos, Jose Maria Assunção Moraes-Junior, Orlando Jorge M Torres, Diego Vaz da Silva, Felipe Coimbra, Felipe José Fernández Coimbra, Narimã Marques, Narimã Marques, Silvio Melo Torres, Adriano Sampaio, Carlos Augusto Canteras, Fabio Ferreira, Marcel Autran Machado, Diego Kleinubing, Livia Lellis, Sarah L Brum, Muhammad Gohar, Boiko Atanasov, Mihail Tanev Slavchev, Mihail Slavchev, Nikolay Belev, Panche Krastev, Ivelin Takorov, Nikola Vladov, Radoslav Kostadinov, Tsonka Lukanova, Vassil Mihaylov, Plamen Milchev Chernopolsky, Rossen Madjov, Vasil Markov Bozhkov, Vasil Danielov Kostov, Daniel Kostov, Evgeni Nikolaev, Fabrice Muhezagiro, Jérémie Niyonkuru, Pacifique Irakoze, Elijah Dixon, Elisabeth Lo, Leyo Ruo, Daniel D'Souza, Pablo E Serrano, Anton Skaro, Ephraim Tang, Juan Glinka, Janet Martin, George Zogopoulos, Peter Metrakos, Prosanto Chaudhury, Rodrigo Torres-Quevedo, Alejandro Brañes, Alejandro Brañes, Cristian Diaz, Erwin Buckel, Jean Butte, Nicolas Devaud, Luis Paqui, Kongyuan Wei, Huaizhi Wang, Lei Cai, Shixing Guo, Yiming Chen, Maher Hendi, Tan To Cheung, Carlos Millan, Pedro Argüello, Goran Pavlek, Hrvoje Silovski, Igor Petrovic, Ivan Romic, Jurica Zedelj, Fedor Amic, Marijan Kolovrat, Mislav Rakic, Danko Mikulic, Ivan Štironja, Tomislav Bubalo, Nikolaos Gouvas, Panayiotis Papatheodorou, Thalis Christophides, Lukas Burda, Martin Straka, Dusan Klos, Jana Tesarikova, Martin Loveček, Michal Gregorik, Pavel Skalicky, Christiana Stögerová, Jakub Fichtl, Skalický TomáŠ, Pavel Zaruba, Andrej Nikov, Christoph Tschuor, Mohamed Mohamed, Bassant Sayed, Ahmed Shaheen, Ahmed Farid, Almoatazbellah Attalla, Dalia Fathallah Ibrahim, Dalia Fathallah, Eman Elmzaien, Bothina Magdy, Samer Salah, Ahmed Saleh, Ahmed Abd Elglel Saker, Ahmed Swealem, Esraa Ibrahim Sallam, Hebatullah Rozza, Mahmoud Bassiony, Manar Elhassan, Merna Elmalah, Mohamed Belal, Mohamed El Gohary, Mohamed Atef Hassanin, Nada Elsayed, Shiamaa Aboelfath, Islam El-Sayes, Mosaab Tayiawi, Abdulrahman Altatari, Ahmad Mohammad Altatari, Ahmed Saleh, Mostafa Shehata Qatora, Mohamed Said, Amr Najjar, Farouq Alahmed, Fatimah Mardhiyyah Binti Zamri, Hajer Ealreibi, Hoor Alahmed, Ismaeil Alyasin, Karim Abdelhalim, Maryam Abd Alfatah, Mohamed Abdallah Sharaan, Mohamed Abd El Moneam, Mohamed Abdelalemm, Mohamed Mourad, Najiha Binti Sohaimee, Nour Eldin Abosamak, Nur Mazlia Farzana Binti Suhaimi, Shaher Shokralla, Yomna E Dean, Yousef Tanas, Zuraiha Waffa, Ahmed Nafea, Dina Ramadan, Abdelrahman Abdelaal, Abdelrahman Mahmoud, Ahmed Mahmoud Nafea, Ahmed S A M E Abuali, Islam Korayem, Marina Fahmy, Menna Ibraheem, Mohammed Hamouda, Rana Helaly, Yazan Fayez Khdour, Yazan Khdour, Marina Farag, Abdelrahman Ibrahim, Hajer Ehab Elareibi, Muneera Alboridy, Ahmed Mansour, Mohamed Galal Ragab, Mohamed Naguib, Shrouq Allam, Hagar Abo Elfarag, Abdelrahman Elsakka, Doaa Mannaa, Mostafa Elkeleny, Nurul Ain Batrisyia Suhaimi, Sofia Suhda Binti Mohd Uzir, Sara Nasr, Amro El-Najjar, Mohamed Dohien, Mohamed Dohien, Nermin Osman, Nuran Gad, Mohamed Hassanin, Bashir A Fadel, Eman Hassan Mohamed Hamdan, Fatma Monib, Mahmoud Saad, Ahmed Abbas, Ahmed Mohammed Abu-Elfatth, Hossam Aldein Abd Elazeem, Mohammed Hisham Zayan Abdelhafez, Nehal Omar, Ramy Hassan, Ahmed Mohamed, Samir Hosney Mahmoud, Abobakr Mahfoz Abobakr, Esraa Essam Elsayed Mohamed, Randa Ahmed, Hesham Mahmoud Hamza, Mahmoud Mohammed, Mohamed Ali Marshod, Ahmed Mokhtar Mahmoud Hussein, Ahmed Taha, Islam Ibrahim, Mariam Albatoul Nageh, Mohammed Nageh Fouly, Ramy Abdelrahim Hassan, Ahmed Kamel Ali Mohamed, Mahmoud Hasab Elnabi, Mohamed Salah, Ahmed Youssef Mohamed Ali, Esraa Gamal Ahmed Sayed, Reem Sayad, Mahmoud M Saad, Mohamed Abdelkarem, Nehal Gamal Omar, Alaa Khalifa, Hazem Faragalla, Ahmed Barakat, Ahmed Tarek Mohamed Barakat, Ahmed Elshafey, Mahmoud Fares Eleisawy, Mahmoud Eleisawy, Mohamed Samir Mohamed Zahed, Mohamed Zahed, Mohammed Omer, Mohamed Allam, Yasmeen Abuelnaga, Abdurrahman Abdelzaher, Ahmed Alnimr, Hany Dabbous, Hatem Sayed, Ibrahim Elgarhy, Mahmoud Elmeteini, Mohamed Bahaa, Mostafa Farag, Mouhanad Eid, Omar Anas, Omar Ismail, Omar Nageeb, Reham Lasheen, Samuel Tanyous, Sherein Diab, Youssef Badran, Abdelrahman Fahim, Emad Alazab, Ibrahim Mohamed Elgarhy, Mahmoud Abdeljalil, Marya Hanna, Mira Gobran, Mira Gobran, Mohamed Osama Mohammed Kamel Abdelmawla, Mostafa Nagy, Omar Emad Nageeb, Salma Ramadan, Sherif Abdelmawgoud, Taha Zidan, Yasmeen Abuelnaga, Yasmeen Tarkhan, Ahmed Saad, Ahmed K Awad, Merihan A Elbadawy, Mohamed Abdelmawla, Emad Mansy, Modather Moharam, Mohamed Elabd, Ahmed Eldabour, Lama Elwakil, Marwan Sayed Hassanien, Amr Elnashar, Hossam El-Dien Saleh, Marina Michail, Ahmed Said, Mahmoud El Garhy, Mohamed Bahaa Eldin Ahmed, Omar Anas, Omar Ismail, Kirellos Abboud, Ahmed Nabil, Mahmoud Elfiky, Abdelrahman Murad, Ahmed Azzam, Mohammed A Azab, Selmy Awad, Zeinab Othman, Abdelrahman Mohamed Fahim, Abdurrahman Taha Abdelzaher, Taha Zidan, Reham Abdelrhman, Engy Amgad Nasr Tolis, Mustafa Salem, Hussein Ebrahim, Hussein A Abdelrazek, Noha Abdelmoneim, Dinah Salman, Hussam Saa'd, Dina Ali, Ahmed Farouk, Ahmed Rafaat Mandor, Ahmed Monier, Ahmed Shehta, Amr Kassem, Amr Sanad, Reem Elsaadany, Mohamed M Shaat, Rami Elmorsi, Selmy Awad, Soliman Ghedan, Ahmed Menessy, Dina Elnabawy, Khaled Abdou, Mohamed Abdelmaksoud, Mohamed Hassan, Omnia Elweza, Rahma Elboraei, Ahmed Abdallah, Islam H Metwally, Mohamed Elhamamsy, Ahmed M Fareed, Mohammad Zuhdy, Saleh S Elbalka, Marwa Nasrelden Alansary, Mohammed Omar, Ahmed Abdelfattah Elgharably, Eman Hager, Ammar El Gady, Doaa Sabry Alsharif, Ammar Magdy Shaaban, Doaa Alsharif, Doaa Samaan, Samy Sameh Samy Samaan, Ahmed Oteem, Ammar Magdy Shaaban, Doaa Sabry Alsharif, Samy Samaan, Ahmed Zayed, Ahmed Allam, Ammar El Gady, Doaa Sabry Alsharif, Karim Badr, Salma Elnoamany, Sameh Samy Samaan, Mohamed Ellibady, Emad Ali Ahmed, Ahmed Elbassyiouny, Ahmed Boalot, Helmy Badr, Mohamed Gamal, Mohamed Abuelazm, Zeinab Othman, Abdullah Eldaly, Abdullah Sami Eldaly, Mostafa Essa, Fatma Abdelrahman, Abdelrahman Sarhan, Feras Alsabbagh, Mohamed Abd Allah, Abdulrahman Bayomi, Moaz Salama, margus kivisild, Olav Tammik, Taavi Podramagi, Heikki Huhta, Joonas H Kauppila, Minna Nortunen, Lionel Jouffret, Daniele Sommacale, Raffaele Brustia, Rim Cherif, Katia Lecolle, Mehdi El Amrani, Cesar Beugniez, Stéphanie Truant, Guillaume Piessen, Sebastien Degisors, Aurélien Dupré, Julie Perinel, Mustapha Adham, Olivia Sgarbura, Francois-Regis Souche, Antonio Iannelli, Jean Gugenheim, Natalia Savvala, Olivier Scatton, Renato Lupinacci, Emilia Ragot, Gilles Manceau, Mehdi Karoui, Nicolas Goasguen, Morgan Anyla, Sebastien Gaujoux, Rami Rhaiem, Tullio Piardi, Fabien Robin, Laurent Sulpice, Edouard Roussel, Eloise Papet, Lilian Schwarz, Emanuele Felli, Fabio Giannone, Patrick Pessaux, Irakli Pipia, Kakhi Khutsishvili, Zaza Demetrashvili, Carsten Krones, Hans-Peter Wüllenweber, Isabel Bartella, Carsten Kamphues, Florian Loch, Ioannis Pozios, Orlin Belyaev, Prem Vignesh Mohan, Waldemar Uhl, Dirk Bulian, Niklas Juengling, Panagiotis Thomaidis, Sandra Korn, Thilo Welsch, Ulrich Bork, Christian Praetorius, Jürgen Weitz, Marius Distler, Christian Krautz, Maximilian Brunner, Robert Grützmann, Elena Mazzella, Andreas Hecker, Martin Reichert, Azadeh Azizian, Jochen Gaedcke, Michael Ghadimi, Ali Aghdassi, Jessica Döbereiner, Johannes Klose, Jörg Kleeff, Ulrich Ronellenfitsch, Karl J Oldhafer, Ki Wagner, Tim Reese, Asmus Heumann, Faik G Uzunoglu, Jakob Izbicki, Mara Goetz, Pasquale Scognamiglio, Kim Honselmann, Tobias Keck, Ulrich Wellner, Benjamin Struecker, Christina Hackl, Frank W Brennfleck, Stefan Brunner, Dimitrios Kardassis, Frank Schütze, Gregor A Stavrou, Omid Ghamarnejad, Ralf Metzger, Alfred Koenigsrainer, Silvio Nadalin, Christoph Anthoni, Georgios Makridis, Stefan A Farkas, Stefan Löb, Efstathios Nikou, Nikolaos Tsoukalas, Eugenios Bairamidis, Aliki Vaia, Antonia Prountzopoulou, Evangelos Fradelos, Aristotelis Kechagias, Dionysia Kelgiorgi, Konstantinos Avgerinos, Argyrios Ioannidis, Konstantinos M Konstantinidis, Michael K Konstantinidis, Dimitrios Papakonstantinou, Ilectra Papiri, Nikolaos Michalopoulos, Zoe Petropoulou, Spyridon Christodoulou, Ioannis Margaris, Iosif Chatzialis, Jonida Selmani, Maria Papadoliopoulou, Nikolaos Arkadopoulos, Panagiotis Kokoropoulos, Pantelis Vassiliu, Stavros Parasyris, Theodoros Sidiropoulos, Paraskevas Stamopoulos, Dimitrios Stergiou, Maria Sotiropoulou, Michalis Vaslamatzis, Nikolaos Roukounakis, Stylianos A Kapiris, Stylianos A Kapiris, Vasileios Vougas, Nikolaos Roukounakis, Dimitrios Dimitroulis, Dimitrios Mantas, Eugenia Kotsifa, Eygenia Kotsifa, Nefeli Tomara, Nefeli Kaiti Tomara, Nikolaos Machairas, Panagiotis Dorovinis, Stylianos Kykalos, Theodoros Tsirlis, Andreas Larentzakis, Gavriella Zoi Vrakopoulou, George Tzimas, Spyridon Pagkratis, Ioannis Triantafyllidis, Alexandros Papalampros, Andreas Polydorou, Athanasios Syllaios, Christina Kontopoulou, Dimitrios Politis, Dimitrios Vouros, Dimitrios Schizas, Eleandros Kyros, Evangelos Felekouras, Ioannis Karavokyros, John Griniatsos, Konstantinos Bramis, Konstantinos Toutouzas, Lysandros Karydakis, Manousos Konstadoulakis, Nikolaos Memos, Prodromos Kanavidis, Dimitrios Massaras, Georgios Fragulidis, Maximos Frountzas, Kleoniki Kordeni, Antonios Vezakis, Konstantinos Iliakopoulos, Leonidas Chardalias, Ioannis Kyriazanos, Ioannis Kyriazanos, Meletios Marougkas, Nikolaos Stamos, Triantafyllos Giannakopoulos, Vasileios Kalles, Dimitrios Balalis, Dimitrios Manatakis, Dimitrios Korkolis, Maria Bourazani, Spiros Delis, Dimitrios Cyrochristos, Evangelos Baltagiannis, Georgios Glantzounis, Stylianos Stylianidis, Alexandros Diamantis, Alexandros Valaroutsos, Dimitrios Magouliotis, Dimitrios Zacharoulis, Grigorios Christodoulidis, Konstantinos Tepetes, Konstantinos Perivoliotis, Maria Fergadi, Gregory Tsiotos, Francesk Mulita, Ioannis Maroulis, Michail Vailas, Apollon Zygomalas, Dionysios Karavias, Elissaios Kontis, Ioannis Katsaros, Nikolaos Kopanakis, Andreas Tooulias, Chrysanthos Christou, Dimitrios Raptis, Georgios Katsanos, Nikolaos Beradze, Vasileios Papaziogas, Vasileios N Papadopoulos, Dimitris Giakoustidis, Anastasios Katsourakis, Evripidis Efthymiou, Iosif Chatzis, Achilleas Ntinas, Efthimios Hatzitheoklitos, Konstantinos Tsalis, Pavlos Koustas, Kambaroudis Apostolos, Panagiotis Petras, Savvas Tsaramanidis, Charalampos Iakovidis, Emmanouil Zacharakis, Tamás Marjai, Attila Bursics, Kristóf Dede, Tamás Tölgyes, Andras Vereczkei, Dezsõ Kelemen, Papp Robert, Bhavin Vasavada, Dhaivat Vaishnav, Praful Pawar, Pravin Suryawanshi, R M Shinde, Charoo Piplani, Ayaskanta Singh, Saroj Kanta Sahu, Satyaprakash Ray Choudhary, Rajesh Gupta, Anand Ramamurthy, E Babu, Sreenivasan Karuparthi, Suresh Kumar, Govind Purushothaman, Jeswanth Sathyanesan, N R Venkatesh, Solomon John, Arvind K Singh, Rahul Gupta, Sudhir K Singh, Dharmender Sharma, Kaushal Yadav, Nitin Leekha, Rashmitha Pippari, Manoj Pandey, Neville Joseph Francis, Tarun Kumar, Sundeep Jain, Dharma Ram Poonia, Jeewan Ram Vishnoi, Nivedita Sharma, Puneet Pareek, Rajendar Byshetty, Sanjeev Misra, Vaibhav Varshney, Ramdip Ray, Sumit Gulati, Supriyo Ghatak, Kshaunish Das, Sujan Khamrui, Sukanta Ray, George Mathew Sebastian, Jithin Thulsi Chand, Murali Appukuttan, Arun Chaturvedi, Naseem Akhtar, Puneet Prakash, Sameer Gupta, Shiv Rajan, Vijay Kumar, Abhinav Arun Sonkar, Ahmad Ozair, Vinay Suresh, Sargun Virk, Mohan Narasimhan, Ramesh Ardhanari, Srinivasan Ramachandran, Divakar Jain, Jayapala Reddy Velagala, Somnath Chattopadhyay, Charishma Vodyala, Jayapala Reddy Velagala, Kanchan Motwani, Ramlal Prajapati, Shruti Tilak, Varun Bansal, Raja Kalayarasan, Suryabhan Bhalerao, Induchoodan P S, Meer M Chisthi, Nizarudeen A, Abdul Latheef, Induprabha Yadev, R C Sreekumar, Induprabha Yadev, Viswanathan KV, Durgatosh Pandey, Mayank Tripathi, Ahmad Fathi Fuadi, Erik Prabowo, Ali H Abbood, Hayder Hammoodi, Maytham Aqeel Al-juaifari, Ali Al-Isawi, Sarah Al-Tekreeti, Mustafa Al-Ogaili, Hashim Talib Hashim, Eran Sadot, Roy Apel, Omri Sulimani, Evgeny Solomonov, Ortal Itzhaki, Ron Lavy, Zahar Shapira, Daniele Nicolini, Marco Vivarelli, MD Roberta Rossi, Federico Mocchegiani, Riccardo Memeo, Leonardo Vincenti, Salvatore Fedele, Valeria Andriola, Angela Gurrado, Giovanna Di Meo, Mario Testini, Vincenzo Neri, Andrea Zironda, Arianna Trizzino, Domenico Pinelli, Michele Colledan, Paolo Pizzini, Riccardo Cirelli, M Masetti, Matteo Zanello, Elio Jovine, Laura Mastrangelo, Raffaele Lombardi, Riccardo Casadei, Anna Malpaga, Antonio Frena, Stefan Patauner, Michele Ciola, Jacopo Andreuccetti, Alberto Manzoni, Mohammad Abu Hilal, Nine de Graaf, Marie Sophie Alfano, Sarah Molfino, Gian Luca Baiocchi, Adolfo Pisanu, Alfredo Mellano, Mario Virgilio Papa, Isidoro Di Carlo, Marcello Donati, Michela Zanatta, Prof Francesco Basile, Adelmo Antonucci, Davide Papis, Marina Pighin, Andrea Celotti, Diego Sasia, Fabrizio Allisiardi, Felice Borghi, Francesca Maione, Giorgio Giraudo, Marco Migliore, Sara Salomone, Stefano Giaccardi, Valentina Testa, Marco Giacometti, Sandro Zonta, Antonio Taddei, Matteo Risaliti, Paolo Muiesan, Irene Urciuoli, Lapo Bencini, Luca Moraldi, Alessandro Anastasi, Giuseppe Canonico, Tommaso Nelli, Giuseppe Lo Storto, Fabrizio D'Acapito, Giorgio Ercolani, Leonardo Solaini, Alessandro Cucchetti, Andrea Gardini, Carlo Alberto Pacilio, Andrea Barberis, Marco Filauro, Franco De Cian, Roberto Valente, Stefano Didomenico, Francesco Saverio Papadia, Stefano Di Domenico, Raffaele De Rosa, Andrea Massobrio, Stefano Scabini, Giacomo Carganico, Beatrice Pessia, Federico Sista, Mario Schietroma, Marcello G Spampinato, Stefano Garritano, Stefano D'Ugo, Tiziana Marchese, Edoardo Saladino, Giuseppe Cuticone, Nino Gullá, Alfonso Recordare, Rubina Palumbo, Alessandro Giani, Giovanni Ferrari, Michele Mazzola, Daniele Dondossola, Giorgio Rossi, Lucio Caccamo, Alessandro Zerbi, Gennaro Nappo, Marco Montorsi, Jorgelina Coppa, Michele Droz dit Busset, Vincenzo Mazzaferro, Albert Troci, Alice Frontali, Michele Crespi, Caterina Baldi, Laura Benuzzi, Francesco Ferrara, Marco Stella, Gabriele Capurso, Massimo Falconi, Domenico Tamburrino, Fabrizio Di Benedetto, Paolo Magistri, Roberto Ballarin, Giacomo Zanus, Marco Brizzolari, Fabio Uggeri, Luca Gianotti, Marco Cereda, Daniele Ferraro, Alessandro Iacomino, Daniele Ferraro, Donatella Pisaniello, Giovanni Vennarecci, Donatella Pisaniello, Gianluca Rompianesi, Roberto Ivan Troisi, Renato Patrone, Andrea Belli, Francesco Izzo, Raffaele Palaia, Marte Gianpaolo, Pietro Maida, Tammaro Pasquale, Domenico Bassi, Umberto Cillo, Lucia Moletta, Cosimo Sperti, Simone Serafini, Anna Caterina Milanetto, Claudio Pasquali, Francesca Tolin, Mario Gruppo, Ottavia De Simoni, Salvatore Buscemi, Marco V Marino, Mario Giuffrida, Raffaele Dallavalle, Francesca Calabretto, Lorenzo Cobianchi, Luigi Pugliese, Alessandro Giardino, Giovanni Butturini, Paolo Regi, Emanuele Federico Kauffmann, Gregorio Di Franco, Luca Morelli, Niccolò Furbetta, Niccoló Napoli, Ugo Boggi, Enrico Pinotti, Mauro Montuori, Antonio Giuliani, Maria Lucia Izzo, Nicola Zanini, Luigi Veneroni, Marco Giordano, Gian Marco Palini, Gianluca Garulli, Vincenzo La Vaccara, Agostino Maria de Rose, Felice Giuliante, Francesco Ardito, Andrea Mingoli, Paolo Sapienza, Pierfrancesco Lapolla, Niccolo Petrucciani, Alessandra Cossa, Alessandro Coppola, Elena Belloni, Giuseppe Nigri, Giuseppe Tisone, Roberta Angelico, Tommaso Maria Manzia, Damiano Caputo, Salomone Di Saverio, Alberto Porcu, Teresa Perra, Claudio Feo, Giulia Deiana, Lodovico Sartarelli, Salvatore Pisconti, Valeria Tonini, Damiano Patrono, Francesco Moro, Luca Grasso, Alberto Brolese, Francesco Ciarleglio, Stefano Marcucci, Cristina Nistri, Marco Massani, Stecca Tommaso, Elisa Galasso, Giacomo Zanus, Marco Brizzolari, Maurizio Romano, Serena Rossi, Simone Novello, Alessandro Ferrero, Serena Langella, Serena Armentano, Nadia Russolillo, Lorenzin Dario, Sergio Intini, Terrosu Giovanni, Alfonso Giovanni Recordare, Giovanni Pirozzolo, Rubina Palumbo, Francesco Calabrese, Giorgio Querini, Sandro Zonta, Andrea Caneparo, Marco Giacometti, Maura De Francesco, Alberto Balduzzi, Alfredo Guglielmi, Andrea Ruzzenente, Calogero Iacono, Edoardo Poletto, Giovanni Marchegiani, Giulia Isa, Giuseppe Malleo, Laura Alaimo, Roberto Salvia, Simone Conci, Stefano Francesco Crinò, Tommaso Campagnaro, Isabella Frigerio, Francesco De Marchi, Michele Bonomo, Sara Napetti, Alice Frontali, Andrea Chierici, Christian Cotsoglou, Elson Gjoni, Sissi Paleino, Stefano Granieri, Daisuke Hashimoto, Sohei Satoi, Tomohisa Yamamoto, Kenichiro Uemura, Ippei Matsumoto, Keiko Kamei, Hiromitsu Maehira, Masaji Tani, Satoshi Hirano, Toru Nakamura, Toshimichi Asano, Keiichi Akahoshi, Minoru Tanabe, Takeshi Ishii, Mohamed H Al Saffaf, Mohammad Al Hamoud, Rahaf Khattab, Subhi Alissawi, Anas Hassouneh, Basma Al-Maadani, Mahmoud Bilal Alali, Quasi Ahmad Hmdan, Aiman Obed, Dania Nijadat, Khayry Al-Shami, Manar Al-shami, Maram Mohsen, Rozana Al-Mallah, Safa' Albaba, Mohammad Theab, Noor Massadeh, Reem Abdelrahman Khader Theab, Salah Wardeh, Salahwardeh, Subhi Zahi Alissawi, Anas Mardini, Hamza Ayman Abdelhalem Arabiyat, Hamza Arabiyat, Abdelkareem Al-Hyari, Hazim Ababneh, Mo'taz Fawzat Naffa', Mohammad Musallam Buwaitel, Ayat Al-Mekhlafi, Hebah Rababa, Mohammed Alzoubi, Almuatasim Khamees, Amro Abuleil, Sari Almiani, Khaled Obeidat, Zouhair Amarin, Abdulaziz Al-Samawi, Amr A Al Hammoud, H Hammad, Maeen Ali Alkhadem, Mahmoud R Mahafdah, Mohamed AlSaffaf, Mohamed Alsabah, Mohammad Bani Hani, Mohammad A AL Hamoud, Mohammad W Alzghoul, Rahaf N Khattab, Saeed K Shumrakh, Ansam Rababah, Heyam Alghazo, Tasneem Rababah, Almu'Atasim Khamees, Sajeda Awadi, Sarah Al Sharie, Ahmad Abdelnoor, Duaa Mohammad Shaout, Qais Omarieh, Sara Yassir Abu-Ghazal, Sara Abu-Ghazal, Dua'A Shaout, Sara Yasser Abughazal, Ildar Fakhradiyev, Shynar Tanabayeva, Timur Saliev, Ibragim Issabekov, Zhanat Spatayev, Ho-Seong Han, Yeongsoo Jo, Haralds Plaudis, Katrina Deja Martinsone, Kristaps Atstupens, Mohamad Jawad, Mohamad Khalife, Walid Ghazi Faraj, Fatoom Alowjali, Mohammed N Albaraesi, Wafa Aldressi, Aihab Benamwor, Suhil Ben Ali, Suhil Saleh, Abdulhadi Alshatshat, Eman Younes, Eman Younes, Reyad Ekhmaj, Sumayyah Bahroun, Sumayyah Ghayth Bahroun, Reyad Ekhmaj, Sumayyah Ghayth Bahroun, Dania Burgan, Hajer Abd Alhamed Aalem, Ramadan Kamoka El Hussain, Marwa Morgom, Entisar Alshareea, Amera Ali Dakheel Ellafi, Shoukrie Shoukrie, Taha Mahmoud Omar Shamakhi, Elham Bareig, Malek Abusannuga, Abdulbari Dkhakhni, Ahmed Abdualla Gerwash, Ahmed Gerwash, Ali Zawia, Eman Othman, Muhannud Binnawara, Sabriya Juma Ali Abdalsalam, Sarah Aljamal, Sultan Ahmeed, Wegdan Ibrahim Almabrouk Khalil, Wegdan Khalel, Tasneem Faraj, Fras Elhajdawe, Marwa Emhemed, Osama Salem, Eman Abdulwahed, Wegdan Khalil, Heba Rhuma, Mohamed Alsori, Tahani Mustafa, Sofian Albarouni, Ahmed Albishti, Muhammed Elhadi, Taha Elkhuja, Ahmed Msherghi, Najat Shaban Ben Hasan, Hayat Ben Hasan, Najat Ben Hasan, Antanas Gulbinas, Giedrius Barauskas, Povilas Ignatavicius, Romualdas Riauka, Tomas Vanagas, Algirdas Slepavicius, Jonas Jurgaitis, Sarunas Dailidenas, Vitalijus Eismontas, Vytenis Mikutaitis, Algirdas Šlepavičius, Jonas Jurgaitis, Vytenis Mikutaitis, Audrius Dulskas, Justas Kuliavas, Laura Aniukstyte, Audrius Sileikis, Aiste Gulla, Audrius Šileikis, Jaroslav Tumas, Kestutis Strupas, Marius Petrulionis, Mindaugas Kvietkauskas, Kestutis Strupas, Deblasi Vito, Edoardo Rosso, Jih Huei Tan, Andee Dzulkarnaen Zakaria, Ikhwan Sani Mohamad, Leow Voon Meng, Teoh Zhan Huai, Firdaus Hayati, Harivinthan Sellappan, Thanesh Kumar Maiyauen, Azlanudin Azman, Ian Chik, Zamri Zuhdi, Boon Yoong, Koh Peng Soon, Koong Jun Kit, Boon Koon Yoong, Jun Kit Koong, Peng Soon Koh, Aini Ibrahim, Nik Azim Nik Abdullah, Jin Bong, Shahi Ghani, Carlos Florez Zorrilla, Miguel Charco Cruz, Andre Moguel Valladares, Ismael Dominguez-Rosado, Alejandro Eduardo Padilla Rosciano, Garcia-Herrera Sebastian, Javier Melchor-Ruan, Juan Sebastian Garcia-Herrera, Erdene Sandag, Sarnai Erdene, Sergelen Orgoi, Moniba Korch, Ahmed Sami Boutti, yassmine boumzebra, Yassmine Boumzebra, Fatine Hourri, Iltimass Gouazar, Wafae Ait Belaid, Badr Serji, Bouhout Tarik, El Harroudi Tijani, Aziz Zentar, Abdelilah Ghannam, Ahmed Bounaim, Amine Souadka, Amine Benkabbou, Brahim El Ahmadi, Houmada Amina, Lahnaoui Oumayma, Laila Amrani, Mohammed Anass Majbar, Raouf Mohsine, Reda Elhassouni, Sabrillah Echiguer, Zakaria Belkhadir, Abdulrashid Pueya Nashidengo, Francis Quayson, John Abebrese, Pueya Nashidengo, Krishna Mohan Adhikari, Paleswan Joshi Lakhey, Ramesh Singh Bhandari, Marc G Besselink, Matthanja Bieze, Simone Augustinus, Olivier Busch, B K Pranger, F J H Hoogwater, J M Klaase, M Meerdink, M W Nijkamp, VE de Meijer, Bas Groot Koerkamp, Casper HJ van Eijck, Jacob L Van Dam, Louise Barbier, Peter Johnston, Richard Babor, Michael Jen Jie Chu, Tiffany Oliver, Daniel Wen, Jonathan Koea, Jonathan Koea, Lisa Brown, Sanket Srinivasa, Adam Bartlett, John Windsor, Peter Carr-Boyd, Vanshay Bindra, Andrea Cross, Saxon Connor, Todd Hore, Ashok Gunawardene, Fraser Welsh, Monique Mahadik, Alexandra Gordon, Jeremy Rossaak, Ademola Adeyeye, Elizabeth Enoch, Victor Kayode-Nissi, Henry Abiyere, Olusegun Alatise, Andrew Okomayin, Clement Odion, Esteem Tagar, Abdulrahaman Abba Sheshe, Abubakar Bala Muhammad, Ibrahim Umar Garzali, Peter Ajayi, Exhevit Kadri, Salah Al Jabri, Yahya Al Azri, Khuwaja Muahammad Inam Pal, Tayyab Siddiqui, Usama Waqar, Usama Waqar, Ahmad Areeb Chaudhry, Jibran Abbasy, Muhammad Osama Khan, Syed Shafqatullah, Muhammad Imran Khokhar, Ali Akbar, Ameer Afzal, Mohammad Asghar, Sami Ullah, Usman Ismat Butt, Usman Butt, Hassaan Bari, Bilal Nabil Mohammad, Mahmoud Hameda, Mustafa Abu Jayyab, Asmaa Hasan Mohammad Alzabadiah, Islam Adam, Khalil Abuzaina, Mohammad Farid, Mohammad Farid Mohammad Emar, Mohammad Emar, Qusai Zreqat, Rawand Titi, Sarah Ameen Idkiedek, Sarah Amro, Shahd Al-Qasrawi, Tabarak Abedlnaser Almasri, Walaa Mohammed Alnammourah, Gharam Kiswani, Raghida Sinnokrot, Zahrah Abu Harb, Hiba Nafa'A, Lyana Shtewi, Abrar Omar Salah, Aseef B A Joma, Sireen Faraj, Abdullah Zitawi, Ahmad Jamal Dawood, Ibraheem Saadeh, Alaa Hmeedan, Motaz Ayman Mahmoud Daraghmeh, Amani Netham Atia Janajreh, Fatima Manassra, Laya Moayyad Ahmed Yassin, Raya Yassin, Abrar Omar Saleh, Sireen Mahmoud Faraj, Abdallah S Sulaiman, Zain Khayyat, Aseef B A Joma, Eman Shawahni, Abrar Salah, Abdullatef khader, Ahlam Hammoudeh, Aram Abdulhaq, Reem Alawna, Gilbert Roman, Javier Targarona, Rafael Garatea Grau, Raquel Molina, Cesar Rodriguez Alegria, Guillermo Coayla, Juan Carlos Marcos Enriquez, Juan Carlos Marcos, Alyssa Nicole Hasiman, Catherine Teh, Ruby Cerdeño, Avril David, Ray I Sarmiento, Ryan Ruel Barroso, Cenon Alfonso, Dr Dante Ang, Amornetta Casupang, Monica Mamuric, Jose Mari Jardinero, Agata Motyka, Marta Flisińska, Stanisław Pierściński, Slawomir Mrowiec, Justyna Rymarowicz, Maciej Matyja, Tomasz Wikar, Marek Sierzega, Michał Pędziwiatr, Piotr Richter, Adam Durczynski, Konrad Kosztowny, Wojciech Ciesielski, Aleksander Wardeszkiewicz, Krzysztof Szwedziak, Michal Wlazlak, Oliwia Grzasiak, Patrycja Szewczyk, Piotr Hogendorf, Justyna Wyroślak-Najs, Karol Rawicz-Pruszyński, Katarzyna Sędłak, Michał Solecki, Wojciech Polkowski, Maciej Słodkowski, Michał Wierzchowski, Wojciech Korcz, Lukasz Nazarewski, Oskar Kornasiewicz, Maria Lopes, Rui Miguel Martins, Ruben Martins, Emanuel Vigia, Donzília Sousa Silva, José Davide, Andre Pereira, Nadia Tenreiro, Tiago Castro, Reem Eisa, Bogdan Diaconescu, Cezar Ciubotaru, Ionut Negoi, Valentina Negoiţă, Raluca Bievel Radulescu, Nicolae Bacalbaşa, Simona Dima, Traian Dumitrascu, Andrada Spanu, Mara Mardare, Octav Ginghina, Eduard Catrina, Iulian Brezean, Mihaela Misca, Mihaela Vilcu, Sorin Aldoescu, Sorin Petrea, Adrian Bartos, Cioltean Cristian Liviu, Ioana Iancu, Sorin Traian Barbu, Raluca Bodea, Emil Mois, Graur Florin, Nadim al Hajjar, Sergiu Matei, Florin Zaharie, Viorel Scripcariu, Ana-Maria Musina, Cristian Ene Roata, Gabriel Mihali Dimofte, Natalia Velenciuc, Sorinel Lunca, Wee Liam Ong, Wee Liam Ong, Ciprian Duta, Dan Brebu, Vlad Braicu, Alexander Belyaev, Alexey Popov, Anastasia Batova, Anastasiia Katysheva, Denis Mizgirev, Liudmila Neledova, Boris Duberman, Andrey Litvin, Artem Pobelenko, Georgy Kuznetsov, Igor Khatkov, Pavel Tyutyunnik, Roman Izrailov, Arkady Bedzhanyan, Konstantin Petrenko, Mikhail Bredikhin, Dr Garnik Shatverian, Nikita Chardarov, Nikolay Bagmet, Vladimir Lyadov, Daniil Mudryak, Ivan Semenenko, Mark Tokarev, Andrey Kriger, Ayrat Kaldarov, Gennady Ivanov, Denis Kuchin, Gaik Torgomyan, Vladimir Zagainov, Vasili Davydkin, Andrey Igorevich Baranov, Evgeniy Drozdov, Li Natalya Anatolievna, Abakar Abdullaev, Mahir Gachabayov, Mohammed Ghunaim, Mohammed Alharthi, Murad Aljiffry, Marko Bogdanovic, Marko Zivanovic, Aleksandar Bogdanovic, Daniel Galun, Vladimir Dugalic, Dragana Arbutina, Ljiljana Milic, Mihailo Bezmarevic, Andrija Antic, Dejan Radenkovic, Igor Ignjatovic, Predrag Zdujic, Stefan Kmezic, Aleksandar Karamarkovic, Dragana Arbutina, Jovan Juloski, Radisav Radulovic, Radosav Radulović, Vladica Cuk, Ljiljana Jeremic, Milan Radojkovic, Miroslav Stojanovic, Danica Golijanin, Milana Kresoja Ignjatovic, Mladjan Protic, Adrian Chiow, Lee Lip Seng, Nita Thiruchelvam, Brian Goh Kim Poh, Brian K P Goh, Darren Chua Wei Quan, Ye Xin Koh, Blaž TrotovŠek, Miha Petrič, Mihajlo Djokić, Ales Tomazic, David Badovinac, Emil Loots, Leanne Prodehl, Mohammed Uzayr Khan, Thomas Marumo, John WS Devar, Jones Omoshoro-Jones, Zafar A Khan, Ben Jugmohan, Ana Quiroga Valcarcel, Belén Matías García, Javier Mínguez, Manuel Marcello, Jose Ramia, Antonio Compañ, Carlos Fernandes, Miguel Morales, Jose Miguel Vargas Fernández, Maria Del Mar Rico-Morales, Miguel ángel Lorenzo Liñán, Joan Figueras, Ramon Soliva, Eugenia Butori, Constantino Fondevila, Fabio Ausania, Belén Martín, Manuel Rodríguez, Santiago Sánchez-Cabús, Patricia Sánchez-Velázquez, Ana Belen Martin Arnau, Ramón Soliva Domínguez, Benedetto Ielpo, Fernando Burdío Pinilla, Maria Castro, David Padilla Valverde, Esther Pilar García Santos, María del Carmen Manzanares Campillo, Patricia Ruiz, Ernesto Castro Gutierrez, Laia Falgueras, Maria Teresa Albiol Quer, Farah Al Shwely, Raquel Latorre Fragua, Daniel Bejarano Gonzalez-Serna, Marcos Alba Valmorisco, Pablo Beltran-Miranda, Juli Busquets, Lluis Secanella, Nuria Pelaez, Gabriel Plaza, Marta Lourdes Gonzalez Duaigües, Pablo Muriel álvarez, Alfredo Escartín, Carmelo Loinaz, Jana Dziakova, Sofia de la Serna, Elia Pérez-Aguirre, Iago Justo, Jorge Saavedra, Jose Castell Gomez, Nuria Losa Boñar, Elena Martín-Perez, Marcello Di Martino, ángela de la Hoz Rogriguez, Alberto Marcacuzco, Carlos Jiménez-Romero, Jorge F Roldán de la Rúa, Luis C Hinojosa-Arco, Miguel ángel Suárez-Muñoz, David Ferreras Martinez, Francisco Sanchez-Bueno, Pedro Gil Vazquez, Alberto Miyar de León, Elisa Contreras Saiz, Lorena Solar García, Ignacio Gonzalez-Pinto, José Carlos Rodríguez-Pino, Juan José Segura-Sampedro, Rafa Morales, Rafael Morales-Soriano, Fernando Rotellar, Gabriel Zozaya, Pablo Martí-Cruchaga, Jaime López-Sánchez, Luis Muñoz-Bellvis, Angel Cuadrado, Irene ortega, Rocio Fernández, Daniel Díaz Gómez, Valle Vera, Javier Padillo Padillo, Juan Bellido Luque, Erik Ilacer Millan, Rosa Jorba, María Isabel García-Domingot, Carlos Redondo, Dra Míriam Cantos, Enrique Artigues, Carlos Domingo-Del Pozo, Carmen Payá Llorente, Sergio Navarro Martínez, Cristina Ballester Ibáñez, Javier Maupoey Ibáñez, Rafael López Andujar, Dimitri Dorcaratto, Elena Muñoz Forner, Marina Garces-Albir, Juan Beltran de Heredia, Mario Montes-Manrique, Mario Rodriguez-Lopez, Alejandro Serrablo, Daniel Milian, Pablo Ruiz-Quijano, Sandra Paterna-Lopez, Arinda Dharmapala, B K Dassanayake, K B Galketiya, Ahmed Mohamed Ibrahim, Hytham Hamid, Nassir Alhaboob, Abdelfatah Abdelmageed, Samah Suliman Osman Taha, Caroline Vilhav, Johanna Hansson Wennerblom, Svein Olav Bratlie, Bergthor Bjornsson, Linda Lundgren, Per Sandström, Bobby Tingstedt, Roland Andersson, Bodil Andersson, Caroline Williamsson, Ernesto Sparrelid, Marcus Holmberg, Poya Ghorbani, Ioannis Gkekas, Christoph Kuemmerli, Martin Bolli, Andreas Andreou, Anna Silvia Wenning, Beat Gloor, Andrea Peloso, Christian Toso, Graziano Oldani, Beat Moeckli, Charles-Henri Wassmer, Alessandra Cristaudi, Majno-Hurst Pietro, Pietro Edoardo Majno-Hurst, Raffaello Roesel, Fariba Abbassi, Ignazio Tarantino, Thomas Steffen, Carlo Ferrari, Jan Schmidt, Olga Meier, Markus Weber, Stefan Gutknecht, Jan Philipp Jonas, Pierre-Alain Clavien, Ahmad Al-Haj, Ahmad Aljaber, Ahmad Amir Kayali, Lama Kadoura, Ezzeldin Nashed, Hala Helaly, Hasan Kayali, Mais Alhashemi, Marwa Aloulou, Mohammed Alshaghel, Nihad Mahli, Omar Al-Abed, Oula Azizeh, Sana Shaikh Torab, Wael Alkhaleel, Marwan Al Aliwy, Omar Alannaz, Ahmad Ghazal, Ruqaya Masri, Zain Douba, Ahmed Saeed Saad, Aya Abdulmonem, Mahmoud Shaban, Ahmad Nabil Alhouri, Ahmad Alhouri, Alnour Soliman, Hasan Nabil Al Houri, Hasan Al Houri, Sarah Omran, Aram Abbas, Majd Chaaban, Mhd Adib Al Kudmani, Mohammad Karam Chaaban, Riad Alhmaidi, Amal Yousef, Amal Youssef, Muhammad Nasri, Hossain Alkhateb, Abdulrahman Almjersah, Naya Hassan, Ahmed Moussa, Alaa Hamdan, Ali Hammed, Ali Alloush, Bashar Haj Hassan, Hala Issa, Hiba Talal Dahhan, Mahmoud Souliman, Salah Hammed, Tharaa Mehdi Tobba, Alaa Hamdan, Seba Ayoub, Ming-Chin Yu, Po-Chih Yang, Chien Hui Wu, Hanen Bouaziz, Khaled Rahal, Skander Slim, Ayed Karim, Oussama Baraket, Ali Kchaou, Ammar Houssem, Mohamed Amine Said, Mohamed Ben Mabrouk, Karim Ben Hamida, Montassar Ghalleb, Ahmed Ben Mahmoud, Houcine Maghrebi, Montasser Jameleddine Kacem, Mesut Tez, N Eminesariipek, özhan çetiindağ, Acar Tüzüner, Kaan Karayalçin, Ahmet Cihangir Emral, Kursat Dikmen, Mustafa Kerem, Hüseyin Bayhan, Mehmet Akif Türkoğlu, Nidal Iflazoğlu, Ahmet özet, Ulaş Aday, özcem öfkeli, Alpen Gumusoglu, Hamit Ahmet Kabuli, Mehmet Karabulut, Kivanc Peker, Sezer Saglam, Fatema Sayed İsmail Rahimi, Fatema Hanefa, Arda Isik, Ertugrul Goksoy, Ender Dulundu, Ali Emre Atici, Aysegul Bahar Ozocak, Cumhur Yegen, Ahmet Cem Dural, Nuri Alper Sahbaz, Hanife Seyda Ulgur, Husnu Aydin, Omer Faruk Ozkan, Ozgul Duzgun, Muhammet çelik, Salih Pekmezci, Ahmet çoker, Alper Uguz, Omer Vedat Unalp, Ismail Sert, Suleyman Ertekin, Mucahit Ozbilgin, Serdar Aydoğan, Enver Tekin, Bulent Calik, Degercan Yesilyurt, Semra Demirli Atici, Türkmen Bahadır Arıkan, Turkmen Arıkan, Emre Gonullu, Enis Dikicier, Recayi Capoglu, Zulfu Bayhan, Sarah Alfurais, Elif Colak, Suleyman Polat, Ahmet Burak Çiftci, James Milburn, Claire Jones, David Vass, Mark Taylor, Bobby VM Dasari, Ambareen Kausar, Asma Sultana, Daren Subar, Quentin Nunes, James Skipworth, Obi Nwogwugwu, Stijn van Laarhoven, Amar Kourdouli, Altaf Awan Awan, Imran Bhatti, Javed Latif, Fiona Hand, Francis Robertson, David Holroyd, David Holroyd, Nigel Jamieson, William Lim, David Chang, Adam Frampton, Rajiv Lahiri, Saurav Chakravartty, Harris Siddique, Manahil Bashir, Stephen Mcnally, Alistair Young, Andrew Smith, James Pine, Giuseppe Garcea, Jonathan Haqq, Deep Malde, Declan Dunne, Isobel Burridge, Peter Szatmary, Deepak Hariharan, Hemant Kocher, Vincent Yip, Amjad Khalil, Ashitha Mohandas Nair, Irene Liova, Aleem O'Balogun, Alex Rothnie, Bhargava Chikkala, Camila Hidalgo Salinas, Carlo Frola, Charalampos Tsakiris, Dimitri Raptis, Dimitrios Chasiotis, Dinesh Sharma, Fatema Jessa, Fiammetta Soggiu, Giuseppe Fusai, Ioannis Kostakis, Manikandan Kathirvel, Mohamed Elnagar, Nikolaos Dimitrokallis, Satheesh Iype, Stephanos Pericleous, Ahmed Mohamed, Alejandro Ramirez-Del Val, Pascale Tinguely, Marina Likos-Corbett, Iqraa Afzal, Ricky Bhogal, Krishnakumure Patel, Ajith K Siriwardena, Nicola de' Liguori Carino, Professor Aali Sheen, Fahed Gareb, Khaled Ammar, Rohan Thakkar, Sanjay Pandanaboyana, John Leeds, Dhanny Gomez, Gordon Gregory, Carlo Ceresa, Hussain Abbas, Lucia Lazzereschi, Srikanth Reddy, Alex Gordon-Weeks, Somaiah Aroori, Thomas Russell, Keith Roberts, Nikolaos Chatzizacharias, Robert Sutcliffe, Bilal Al-Sarireh, Guy Shingler, Matt Mortimer, Denys Skoryi, Ievgen Ilin, Margaryta Pisetska, Dmytro Cheverdiuk, Kopchak Kostyantyn, Kostiantyn Kopchak, Oleksandr Kvasivka, Sumarokova Valeriia, Valeriia Sumarokova, Vitalii Kryzhevskyi, Sergei Sikachov, Andrii Khomiak, Andrii Malik, Igor Khomiak, Andriy Bilyak, Serge Chooklin, Serhii Chuklin, Iurii Mikheiev, Oleh Shylenko, Andrii Klymenko, Shirali Patel, Steven Cunningham, Mark Callery, Tara Kent, Chandrajit Raut, Jiping Wang, Mark Fairweather, Megan Sulciner, Sameer Hirji, Thomas Clancy, Martina Nebbia, Motaz Qadan, Amanda Musser, Melissa Hogg, Jennifer Rodriquez, John Hamner, Liz Hennessy, Aaron Dinerman, Amar Gupta, Charles Kimbrough, Rachel Thompson, Herbert J Zeh, Imad Radi, Patricio M Polanco, Dimitrios Moris, Michael E Lidsky, David Lee, James Piper, Jennifer Gnerlich, Daniel Tuvin, Robert Sticca, Sabha Ganai, Niraj Gusani, Derek Krinock, Emmanouil Giorgakis, Hailey Hardgrave, Richard T Spencer-Cole, Garrett Klutts, Hailey Hardgrave, Joe Nigh, Joseph Nigh, Juan Camilo Barreto Andrade, Michail Mavros, Tamara Osborn, Cristina Ferrone, Victoria O'Connor, Brian Boone, Britney Harris, Carl Schmidt, Beth Schrope, John Chabot, Michael Kluger, Erika Tay Lasso, Avinoam Nevler, Charles Yeo, Francesca Ponzini, Harish Lavu, Ryan Lamm, Wilbur Bowne, Nina Kyser, Christos Galanopoulos, Arezou Abbasi, James Park, Jonathan Sham, Lindsay Dickerson, Venu Pillarisetty, Iswanto Sucandy, Sharona Ross, Emily Winslow, Jasn Hawksworth, Pejman Radkani, Thomas Fishbein, Armando Salim Munoz, James Lindberg, Paulo N Martins, Rafat Ameen Mohammed Al-saban, Rafat Al-Saban, Waheeb Al-Kubati, Asma Ali Ahmed Ghallab, Ghadeer Mohammed Alsanany, Hassan Almarashi, Hytham Al-Samawi, Mohammed Abdulkhaleq Mohammed Mohsen Al-Asadi, Ramzi Alsayadi, Sara Hail, Sarah Shream, Hadeel Mohmmed Bajjah, Saba Al-Ameri, Hadeel Bajjah, Saba Ahmed Ahmed Saleh Al-Ameri, Nagra Are Al-Dowsh, Nagran Aref AlDowsh, Qaeid Al-Khawlani, Yahya Ali Ali Murshed, Mohammed Al-Shehari, Amir Al-Deen Jahaf, Ebrahim Ahmed Esmail Al-sharabi, Hamdan Aldumaini, Zainab Alattas, Ali Almassaudi, Hadeel Muhammed Ahmed Hussein Bajjah, Rudaina Albakry, Hamza Al-Naggar, Sarah Abdulkhaliq Ali Shream, Anter Al Affary, Eman Al-Markiz, Fatima Al-Eryani, Heba Farhat, Qannaf Al Qadasi, Khaled Alwafy, Mahmmoud Yehia Mohammed Abdualqader, Ramzi Ali Abdullah Yahya Ali, Aisha Albar, Hikma Abdullh Bleem, Khaled Sultan Ali Galeb, Mohammed Ghushaim, Mohammed Sabbar, Muhib Esmail, Ramzi Abdullah Yahya Ali, Rana Hassan Mohammed Salem, Rana Salem, Wail Saif, Siham Al-Faiq, Ebrahim Alsharabi, Almekhlafi Tofik Abdul Hameed, Tofik Almekhlafi, Abdulrahman Omairan, Eman Almarkiz, Heba Abduljawad, Omair Mansaleh, Watheeq Al-Melhani, Mahmmoud Abdualqader, Radfan Al-Abdi, Hussein Mohammed Alwan, Chenesa Mbanje, Onesai Chihaka

**Affiliations:** Department of Hepatopancreatobiliary Surgery and Liver Transplant, Royal Free Hospital NHS Foundation Trust, London, UK; Division of Surgical and Interventional Science, University College London, London, UK

## Abstract

**Background:**

Pancreatic surgery remains associated with high morbidity rates. Although postoperative mortality appears to have improved with specialization, the outcomes reported in the literature reflect the activity of highly specialized centres. The aim of this study was to evaluate the outcomes following pancreatic surgery worldwide.

**Methods:**

This was an international, prospective, multicentre, cross-sectional snapshot study of consecutive patients undergoing pancreatic operations worldwide in a 3-month interval in 2021. The primary outcome was postoperative mortality within 90 days of surgery. Multivariable logistic regression was used to explore relationships with Human Development Index (HDI) and other parameters.

**Results:**

A total of 4223 patients from 67 countries were analysed. A complication of any severity was detected in 68.7 per cent of patients (2901 of 4223). Major complication rates (Clavien–Dindo grade at least IIIa) were 24, 18, and 27 per cent, and mortality rates were 10, 5, and 5 per cent in low-to-middle-, high-, and very high-HDI countries respectively. The 90-day postoperative mortality rate was 5.4 per cent (229 of 4223) overall, but was significantly higher in the low-to-middle-HDI group (adjusted OR 2.88, 95 per cent c.i. 1.80 to 4.48). The overall failure-to-rescue rate was 21 per cent; however, it was 41 per cent in low-to-middle- compared with 19 per cent in very high-HDI countries.

**Conclusion:**

Excess mortality in low-to-middle-HDI countries could be attributable to failure to rescue of patients from severe complications. The authors call for a collaborative response from international and regional associations of pancreatic surgeons to address management related to death from postoperative complications to tackle the global disparities in the outcomes of pancreatic surgery (NCT04652271; ISRCTN95140761).

## Teaser

The true global outcomes of pancreatic surgery remain unknown. This international snapshot study revealed a higher mortality rate in low-to-middle-HDI countries despite similar postoperative morbidity, which may be attributable to failure to rescue of patients from severe complications. A collaborative response is warranted to make pancreatic surgery safer worldwide.

## Introduction

Improvements in healthcare, including the delivery of surgical care, have been observed worldwide. These improvements, however, have not been uniform and disparity in the access to surgical treatment between high- and low-to-middle-income countries remains significant^1^. Global surgery, a commitment to advancing surgical care globally, has emerged as a response to address disparities in surgical care^2^.

Pancreatic surgery-associated mortality has reportedly decreased to rates as low as 0–3 per cent^3–5^; however, these figures represent high-volume centres from countries with a high Human Development Index (HDI), a composite metric of life expectancy, education, and income per capita. Improvements in high-HDI countries have been attributed to better patient selection, surgical expertise, and standardization of postoperative care^6^. Several risk factors for postoperative complications have been identified, including age, ASA fitness grade, diabetes mellitus, poor nutritional status, blood loss, perioperative transfusion, and pancreatic texture at surgery^7^. Despite a reduction in perioperative mortality, the associated morbidity rate reported in current literature remains as high as 30–50 per cent^8–10^, suggesting that the management of complications may be key to reducing mortality.

This international study of pancreatic surgery outcomes sought to record a snapshot of global pancreatic surgical practice, allowing analysis of current mortality, morbidity, and practice patterns.

## Methods

### Ethics

The chief investigator in the UK ensured that data recording was carried out in accordance with the Research Governance Framework for Health and Social Care, Second Edition, 2005, and its subsequent amendments. The principal investigator at each participating centre was responsible for their appropriate institutional research committee compliance, which was a prerequisite for data acceptance. In the UK, the National Research Ethics Service decision tool (https://www.hra-decisiontools.org.uk/research/) confirmed that this study would not be considered research by the National Health Service (NHS). This study was therefore registered at the Royal Free London NHS Foundation Trust audit tracker in accordance with Trust policy by the chief investigator (reference number RFH287_20/21). The study was registered prospectively with ClinicalTrials.gov (NCT04652271) and the ISRCTN registry (ISRCTN95140761).

### Study design

The International Pancreatic Surgery Outcomes Study—PancreasGroup.org—was a prospective, multicentre, cross-sectional study undertaken to provide an overview of the current practice of pancreatic surgery worldwide. The study protocol was first introduced to the international community of pancreatic surgeons attending the International Hepato-Pancreato Biliary Association (IHPBA) meeting in 2020. The website was subsequently launched and advertised on social media platforms. Pancreatic surgeons practising across the world who were interested in the study could volunteer as country leaders to contribute to the recruitment of further centres in their respective country. The study design followed the Global Surgery Collaborative Snapshot Research approach^11^ and results are reported according to the STROBE guidelines^12^ (*Table S1*).

### Study interval

The study design was initiated in January 2020 and the project was made available to the public with centre recruitment starting in September 2020. Patient recruitment took place in 2021, with 3-month prospective, consecutive patient enrolment. All patients were followed up prospectively until 90 days after surgery.

### Centre recruitment

This study was announced worldwide through various sources including e-mail lists, universities, hospitals, associations, societies, social media, and personal contacts. Furthermore, country leaders worldwide were assigned the role to recruit centres in their region. The study protocol was translated into 10 different languages and made available on the study platform. Thus, all these efforts were made to avoid centre selection bias.

### Participants and procedures

Recruited sites provided data on all adult consecutive patients undergoing pancreatic surgery. This comprised all indications (benign and malignant), open, laparoscopic or robotic, elective or emergency, partial or total pancreatectomies, as well as pancreatic tumour enucleations, procedures with concomitant vascular or other-organ resections, and surgical pancreatic duct drainage procedures for chronic pancreatitis. Exclusion criteria were: age less than 18 years, pancreas or islet cell transplantation, transcutaneous or transgastric imaging-guided ablation or electroporation, endoscopic procedures, as well as transgastric or surgical necrosectomies. This study aimed to recruit the maximum number of patients worldwide over the study interval. As a patient recruitment target, a 90-day mortality rate of 3 per cent was assumed. The aim was to recruit at least 3000 patients to allow meaningful analysis. To minimize patient selection bias, the participants were aware that data were to be reported anonymously, and not to be shared with any other institutions, societies, or government agencies.

### Data

Patient and operation characteristics were collected. Morbidity until hospital discharge was recorded according to the Clavien–Dindo classification of surgical complications^13^ and the Comprehensive Complication Index®^14^ (LGID Foundation, 8008 Zurich, Switzerland) until 90 days after surgery. Major complications were defined as those with a Clavien–Dindo grade of at least IIIa, indicating any postoperative complication requiring an intervention, the patient developing organ failure, or the complication leading to death. The failure-to-rescue rate^15^ was calculated by dividing the number of patients who died by the total number of patients with major postoperative complications. Data were collected via a case report form (CRF) available from the PancreasGroup.org platform^16^. This form was specially designed to include mandatory fields for case submission, including outcome data to ensure that there were no missing data important for the analysis. Cases with incomplete data were labelled as draft and were excluded from the analysis (*Table S3*) For cancer resections, data collection followed the AJCC, 8th edition, staging system^17^. Participating countries were classified into low-to-middle-, high-, and very high-HDI countries according to the United Nations Development Program 2021 report^18^. Briefly, HDI is a statistical composite index of life expectancy, education (mean years of schooling completed and expected years of schooling upon entering the education system), and per-capita income indicators, which is used to rank countries into four tiers of human development.

### Complexity score

A new score of complexity of pancreatic surgery was developed based on seven clinically relevant parameters according to review of clinical data. Each parameter was associated with an increased risk of complications after pancreatic surgery in previous studies, clinical experiences, and in this data set. Receiver operating characteristic curve analysis was used to assess its predictive value, and the Youden's index was used to identify optimal cut-off points for morbidity and mortality. The score was further assessed in a separate multivariable analysis of mortality. For practical purposes, each of the following seven parameters was assigned a single point (that is equal weight) so that it can be used in daily clinical practice without the need for sophisticated calculators: BMI over 35 kg/m^2^, soft pancreas, pancreatic duct smaller than 3 mm, pancreatoduodenectomy or total pancreatectomy, portomesenteric venous resection and reconstruction, arterial resection and reconstruction, and procedure extended to resection of additional organs (for example colon).

### Statistical analysis

Continuous variables were compared using the Mann–Whitney *U* test or Kruskal–Wallis *H* test, as appropriate. Differences among proportions were analysed using Fisher’s exact test or Pearson’s χ^2^ test, as appropriate. Patients with missing data on hospital stay as well as duplicate submissions were excluded from the analysis. No other patients were excluded as key CRF data were mandatory. Multivariable logistic regression was used to investigate 90-day mortality. All *P* values were two-sided and *P* < 0.050 was considered statistically significant. Statistical analysis was undertaken using R version 3.3.2 (R Core Team, R Foundation for Statistical Computing, Vienna, Austria), and R Studio version 1.0.44 (RStudio, Boston, MA, USA) with the graphical user interface rBiostatistics.com© (rBiostatistics.com, London, UK).

## Results

### Participants

A total of 4223 patients were included in the analysis after excluding 123 submitted cases (2.8 per cent) with incomplete hospital stay data and 5 (0.1 per cent) sample case entries used to test the online CRF. This cohort was derived from all 7 continents, 67 countries, 255 cities, and 354 institutions; however, 641 institutions from 81 countries initially registered with PancreasGroup.org (*Figs S2–S4*). Overall demographics revealed that patients of both sexes were similarly represented; the median age was 64 (i.q.r. 55–72) years, and the median BMI was 24.7 (22.0–27.7) kg/m^2^ (*Table S2*). A majority of patients (2129 of 4223, 50.4 per cent) had an ASA grade of II; the most common co-morbidities were diabetes mellitus (1146 of 4223, 27.1 per cent) and cardiac disease (789 of 4223, 18.7 per cent).

### Operation characteristics

Overall, 679 patients (16.1 per cent) underwent minimally invasive surgery, including 189 (4.5 per cent) who had robotic pancreatic surgery (*Table 1*). Portomesenteric venous resection was required in 504 patients (11.9 per cent), whereas 119 (2.8 per cent) underwent arterial resection. End-to-end and tangential reconstruction modalities were most commonly used in portomesenteric resections (265 (51.6 per cent) and 217 (42.2 per cent) respectively), whereas graft reconstruction was required in 73 patients (14.2 per cent). The median intraoperative estimated blood loss was 300 (i.q.r. 150–500) ml and mean(s.d.) packed red blood cell transfusion amount was 0.5(1.5) units. The mean(s.d.) complexity of pancreatic surgery score was 1.8(1.0) (range 0–6).

**Table 1:** Operation characteristics

	No. of patients* (*n* = 4223)
**Surgical approach**	
Open	3544 (83.9)
Laparoscopic	490 (11.6)
Converted to open	105 (2.5)
Robotic	189 (4.5)
Converted to open	24 (0.6)
Duration of operation (min), median (i.q.r.)	320 (240–420)
**Operative procedure**	
Distal pancreatectomy	1033 (24.5)
Spleen-preserving	194 (4.6)
Enucleation	62 (1.5)
Pancreatoduodenectomy	2501 (59.3)
Pylorus-preserving	808 (19.1)
Total pancreatectomy	291 (6.9)
Spleen-preserving	61 (1.5)
Other	219 (5.2)
Extended procedure to additional organs	599 (14.2)
**Vessel resection and reconstruction**	
Portomesenteric resection	504 (11.9)
Tangential reconstruction	217 (42.2)
End-to-end reconstruction	265 (51.6)
Autologous or cadaveric graft reconstruction	43 (8.4)
Prosthetic graft (biological or synthetic)	30 (5.8)
Arterial resection	119 (2.8)
**Intraoperative findings**	
Pancreatic texture	
Hard/fibrotic	1493 (41.4)
Soft/normal	2112 (58.5)
Pancreatic duct size (mm)†	
< 3	1178 (37.0)
3–8	1762 (55.3)
>8	245 (7.7)
**Intraoperative blood loss**	
Estimated blood loss (ml), median (i.q.r.)	300 (150–500)
Blood transfusion (units), mean(s.d.)	0.5(1.5)
**Other information**	
Use of surgical drain	4071 (96.5)
Intraoperative octreotide administration	1495 (35.7)

^*^Values are *n* (%) unless otherwise indicated. †Where applicable.

The two most frequently performed operations were pancreatoduodenectomy (2501, 59.3 per cent) and distal pancreatectomy (1033, 24.5 per cent) (*Table S3*). Of all distal pancreatectomies, only 381 (36.5 per cent) were carried out minimally invasively, and 194 patients (18.8 per cent) underwent a spleen-preserving distal pancreatectomy. Interestingly, a total of 56 different combinations of pancreatic stump closure was identified in patients undergoing distal pancreatectomy. The most frequently used pancreatic stump closure techniques were handsewn (268, 25.8 per cent), stapler (542, 52.2 per cent), and reinforced staple line (175, 16.9 per cent).

### Pancreatic ductal adenocarcinoma

Based on histopathology results, an operation was performed for cancer in 3299 patients (78.1 per cent), of whom 1894 (45.1 per cent) were found to have pancreatic ductal adenocarcinoma (*Table 2*). Neoadjuvant therapy was administered to 445 patients with pancreatic ductal adenocarcinoma (23.4 per cent). Portomesenteric venous resection was required in 392 patients (20.7 per cent) to facilitate resection. Although 449 patients (25.4 per cent) who underwent pancreatoduodenectomy had an incomplete (R1) resection on histopathology, of those undergoing portomesenteric vein resection, only 20 of 392 (5 per cent) had a positive portal vein resection margin. The most frequent positive specific resection margins on histopathology were the posterior (214 of 1894), superior mesenteric vein (124 of 1894), and superior mesenteric (92 of 1894) margins.

**Table 2:** Characteristics of patients diagnosed with pancreatic ductal adenocarcinoma

	No. of patients* (*n* = 1894)
**Patient and disease characteristics**	
Age (years), median (i.q.r.)	67 (59–73)
Sex ratio (F : M)	926 : 968
BMI (kg/m^2^), median (i.q.r.)	24.6 (22.0–27.4)
Preoperative CA19-9 (units/l), median (i.q.r.)	78 (22–310)
**Preoperative treatment**	
Neoadjuvant therapy	445 (23.4)
Neoadjuvant chemotherapy	363 (19.2)
Neoadjuvant radiotherapy	79 (4.2)
**Operative procedure**	
Pancreatoduodenectomy	1335 (70.5)
Distal pancreatectomy	350 (18.5)
Total pancreatectomy	160 (8.5)
Other	48 (2.5)
Extended procedure (additional organs resected)	252 (13.3)
**Vessel resection and reconstruction**	
Portomesenteric resection	392 (20.7)
Arterial resection	75 (3.9)
**Final pathological diagnosis**†	
Tumour size (mm), median (i.q.r.)	30 (22–40)
Total no. of lymph nodes resected, median (i.q.r.)	20 (13–28)
Total number of positive lymph nodes resected, median (i.q.r.)	1 (0–4)
Lymphovascular invasion	1085 (63.5)
Perineural invasion	1324 (77.0)
Portal vein involvement	188 (14.1)
Disease stage (*n* = 1744)	
0	20 (1.2)
IA	186 (10.8)
IB	291 (16.8)
IIA	155 (9.0)
IIB	578 (33.5)
III	403 (23.4)
IV	109 (5.3)
Resection margins (*n* = 1771)	
R0	1300 (73.4)
R1	449 (25.4)
R2	22 (1.2)
Specific positive pancreatic resection margins (*n* = 1894)	
Anterior surface	62 (3.3)
Posterior margin	214 (11.3)
SMV margin	124 (6.6)
SMA margin	92 (4.9)
Pancreatic neck/transection margin	91 (4.8)
Proximal duodenal/gastric margin	15 (0.8)
Common bile duct margin	19 (1.0)
Distal duodenal margin	10 (0.5)
Portal vein resection margin	20 (1.0)
Other	23 (1.2)

^*^Values are *n* (%) unless otherwise indicated. †Data available for variable number of patients. CA19-9, carbohydrate antigen 19-9; SMV, superior mesenteric vein; SMA, superior mesenteric artery.

### Postoperative outcomes

Postoperative outcomes were recorded until hospital discharge and 90 days after operation (*Table 3*). Of all 4223 patients, 2901 (68.7 per cent) experienced a complication of any severity, 1873 (73.6 per cent) after pancreatoduodenectomy and 634 (60.8 per cent) after distal pancreatectomy. Major complication rates (Clavien–Dindo grade at least grade IIIa) were higher after pancreatoduodenectomy than distal pancreatectomy: 717 of 2554 (28 per cent) *versus* 203 of 1043 (20 per cent) (*P* < 0.001). The most frequent complications were pancreatic fistula (1053 of 4223, 24.9 per cent), infection (890 of 4223, 21.0 per cent), and delayed gastric emptying (800 of 4223, 18.9 per cent). The rate of major complications (Clavien–Dindo grade at least IIIa) was 25.8 per cent (1090 of 4223). The 90-day postoperative mortality rate was 5.4 per cent (229 of 4223) overall, 6.2 per cent (157 of 2554) after pancreatoduodenectomy and 1.9 per cent (20 of 1043) after distal pancreatectomy. The failure-to-rescue rate was 21.0 per cent (229 of 1090) overall. Specifically, however, it was 21.9 per cent (157 of 717) after pancreatoduodenectomy and 10.0 per cent (20 of 203) after distal pancreatectomy (*Table 3*).

**Table 3:** Postoperative outcomes

	Overall (*n* = 4223)	Pancreatoduodenectomy (*n* = 2501)	Distal pancreatectomy (*n* = 1033)
**Highest Clavien–Dindo complication grade at 90 days**			
No complications	1322 (31.3)	671 (11.8)	409 (39.2)
Grade I—no treatment	744 (17.6)	450 (17.7)	202 (19.4)
Grade II—drug treatment	1067 (25.3)	706 (27.8)	229 (22.0)
Grade IIIa—intervention under LA	461 (10.9)	297 (11.7)	102 (9.8)
Grade IIIb—intervention under GA	277 (6.6)	182 (7.2)	61 (5.8)
Grade IVa—single organ failure	76 (1.8)	46 (1.8)	15 (1.4)
Grade IVb—multiorgan failure	52 (1.2)	35 (1.4)	5 (0.5)
Grade V—death	229 (5.4)	157 (6.2)	20 (1.9)
**Clavien–Dindo grade at 90 days, grouped**			
Complication of any severity	2901 (68.7)	1873 (73.6)	634 (60.8)
Grade ≥IIIa	1090 (25.8)	717 (28.2)	203 (19.5)
Grade ≥IIIb	629 (14.9)	420 (16.5)	101 (9.7)
**Comprehensive Complication Index^®^ score, median (i.q.r.)**			
Until discharge	8.7 (0–29.6)	15.0 (0–20.9)	8.7 (0–20.9)
Until 90 days after surgery	20.9 (0–33.2)	20.9 (0–35.0)	8.7 (0–26.0)
**Postoperative complications until hospital discharge**			
Delayed gastric emptying	800 (18.9)	598 (23.5)	84 (8.1)
ISGPS grade (*n* = 3127)			
A	415 (10.8)	314 (13.4)	48 (5.0)
B	234 (6.1)	181 (7.7)	17 (8.6)
C	77 (2.0)	58 (2.5)	4 (6.5)
Pancreatic fistula	1053 (24.9)	658 (25.9)	326 (31.3)
ISGPS grade (*n* = 3892)			
A	539 (13.9)	313 (13.3)	167 (19.2)
B	407 (10.4)	253 (10.7)	132 (13.6)
C	105 (2.7)	90 (9.0)	10 (1.0)
Postoperative bleeding	435 (10.3)	310 (12.2)	56 (5.4)
ISGPS grade (*n* = 3434)			
A	142 (3.7)	100 (4.3)	20 (2.1)
B	126 (3.3)	95 (4.1)	19 (2.0)
C	132 (3.4)	100 (4.3)	8 (0.8)
Biliary fistula	187 (4.4)	144 (5.7)	n.a.
Gastrojejunostomy leak	68 (1.6)	43 (1.7)	n.a.
Chyle leak	212 (5.0)	147 (5.8)	33 (3.2)
Portal vein thrombosis	58 (1.4)	29 (1.1)	14 (1.3)
Pulmonary complications	473 (11.2)	307 (12.1)	91 (8.7)
Gastrointestinal complications	349 (8.2)	217 (8.5)	62 (5.9)
Cardiac complications	250 (5.9)	163 (6.4)	48 (4.6)
Urological complications	185 (4.3)	115 (4.5)	34 (3.3)
Infection	890 (21.0)	634 (24.9)	123 (11.8)
Neurological complications	123 (2.9)	70 (2.8)	21 (2.0)
COVID-19	69 (1.6)	45 (1.8)	12 (1.2)
Other	574 (13.6)	334 (13.1)	111 (10.6)
**Treatment offered within 90 days of surgery**			
Insulin administration	929 (25.1)	461 (20.7)	181 (19.8)
Pancreatic enzyme supplementation	2152 (57.3)	1456 (63.8)	358 (39.0)
Chemotherapy offered	1758 (50.1)	1301 (59.9)	292 (33.3)
Radiotherapy offered	106 (2.9)	70 (3.1)	18 (2.0)
**Other postoperative outcomes**			
Duration of IMC/HDU stay (days), median i.q.r.)	0 (0–1)	0 (0–1)	0 (0–1)
Duration of ICU stay (days), median (i.q.r.)	1 (0–2)	1 (0–2)	0 (0–1)
Duration of hospital stay (days), median (i.q.r.)	11 (7–17)	12 (8–19)	8 (6–13)
Hospital readmission rate until 90 days	723 (19.7)	440 (19.7)	184 (20.2)
Failure-to-rescue rate	229 of 1090 (21.0)	157 of 717 (21.9)	20 of 203 (10.0)
Cost (US $), mean(s.d.)	22 317 (15 146)	23 465 (16 661)	19 770 (11 419)

Values are *n* (%) unless otherwise indicated. LA, local anaesthesia; GA, general anaesthesia; ISGPS, International Study Group on Pancreatic Surgery; n.a., not applicable; IMC/HDU, intermediate medical care/high-dependency unit; ICU, intensive care unit.

### Morbidity and mortality within Human Development Index groups

**Fig. 1:**
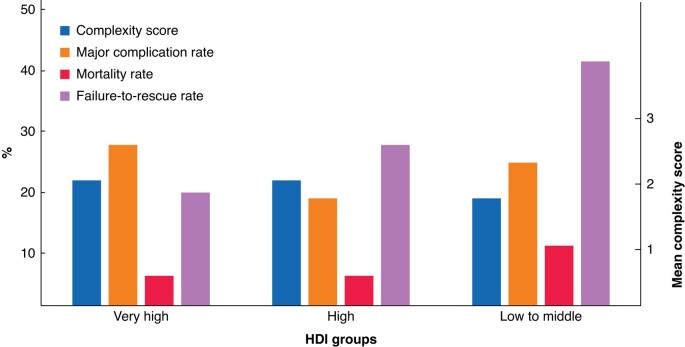
Complexity of pancreatic surgery score, and major complication, mortality, and failure-to-rescue rates among the low-to-middle-, high-, and very high-Human Development Index groups The complexity score bar scale is 10-fold for better interpretation. HDI, Human Development Index.

**Fig. 2:**
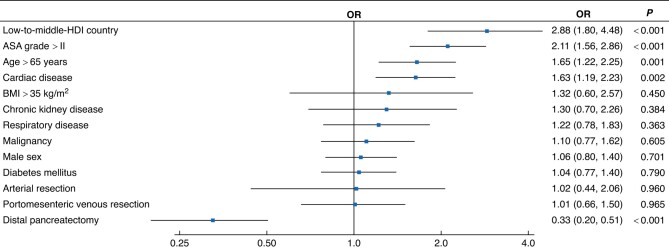
Multivariable binary logistic regression analysis for 90-day mortality ORs are shown with 95% confidence intervals. HDI, Human Development Index.

The HDI reflects the life expectancy, education levels, and income of different countries. Patient, disease, operation characteristics, and postoperative outcomes of patients among the three HDI groups are summarized in *Table S4*. Although the complexity of pancreatic surgery was similar across HDI groups, postoperative morbidity, 90-day mortality, and failure-to-rescue rates differed (*Fig. 1*). Major complication rates (Clavien–Dindo grade at least IIIa) were 24.4 per cent (69 of 285) in low-to-middle-, 18.0 per cent (89 of 494) in high-, and 27.1 per cent (932 of 3444) in very high-HDI countries. Mortality rates were 9.8 per cent (28 of 285), 4.9 per cent (24 of 494), and 5.1 per cent (177 of 3444) respectively. The overall 90-day postoperative mortality rate was 5.4 per cent (229 of 4223), but was significantly higher in the low-to-middle-HDI group (adjusted OR 2.88, 95 per cent c.i. 1.80 to 4.48) (*Fig. 2*). The failure-to-rescue rate in the low-to-middle category was 41 per cent, twice that of the very high-HDI group (19 per cent) (*P* < 0.001).

## Discussion

In this international, prospective 3-month snapshot study of 4223 patients, pancreatic surgery has been shown to be an established treatment modality worldwide. The PancreasGroup.org collaborative is a unique collaboration of pancreatic surgeons across countries of all HDI groups who have contributed to this seminal study investigating the global practice and outcomes of pancreatic surgery.

Reflecting its complexity, the minimally invasive approach remains rare for pancreatoduodenectomy worldwide. Similarly, distal pancreatectomy remains predominantly an open operation, despite wider support in the literature for minimally invasive approaches^19^. However, in this study, nearly one-tenth of distal pancreatectomies were undertaken using the robotic approach, which may well affect the prevalence of minimally invasive surgery in the field of pancreatic surgery in the future. Other examples of practice heterogeneity identified in this global cohort included stump closure technique in distal pancreatectomy and administration of enzyme supplementation. Interestingly, over 50 different stump closure combinations were noted. Over one-third of patients in this cohort did not receive pancreatic enzyme supplementation after surgery, even though the International Study Group on Pancreatic Surgery^20^ has advised universal enzyme replacement therapy after pancreatic surgery.

Evidently, the predominant indication for pancreatic surgery is cancer. Of note, over one-fifth of patients with pancreatic ductal adenocarcinoma received neoadjuvant chemotherapy. This reflects the possibility of improving outcomes with neoadjuvant therapy and the ongoing expansion of the concept of resectability in pancreatic surgery^21–23^. With regard to adjuvant systemic therapy, only half of patients received adjuvant chemotherapy during the 3-month follow-up after surgery, with considerable discrepancy between very high-HDI and low-to-middle-HDI countries, a difference of 28 per cent. Adjuvant chemotherapy has shown to improve overall survival in patients with pancreatic cancer^24–26^ and this difference, therefore, raises concerns regarding the treatment options available to patients in low-to-middle-HDI countries.

Pancreatoduodenectomy is the most commonly performed pancreatic operation, but it is far from being a standardized procedure. From initial descriptions tracing back to the late 1800s^27^, followed by developments in the mid-1900s by Allen Oldfather Whipple^28^, data in favour of different technique modifications continue to emerge. A lack of uniformity in surgical technique and postoperative management was expected as there is little high-quality evidence supporting the use of one technique or management strategy over another^29^. In the present cohort, there was a wide range of surgical and management strategies, reflecting the continued development of this complex operation as well as the lack of high-quality trials comparing techniques. Of note, although once rarely performed, vascular resections have become an integral part of pancreatic surgery^30^. More than 1 in 10 patients undergoing pancreatoduodenectomy also underwent portomesenteric venous resection. Arterial resections were performed in almost 3 per cent of operations; the disease would have been deemed unresectable in all these patients only a decade ago^31,32^.

For meaningful comparison of outcomes, a novel complexity scoring system was developed that considers patient characteristics, pancreatic gland characteristics, and extent of resection, including vascular resection or resection extended to include additional organs. Globally, the major complication and surgical complexity rates after pancreatic surgery were comparable across centres in this study. This may reflect the fact that pancreatic surgery has become a specialist area of surgery globally as opposed to a branch of general surgery with possibly similar levels of surgical skills and training worldwide. Although the major complication and surgical complexity rates were similar, the postoperative 90-day mortality rate was higher in low-to-middle-HDI countries than in high- and very high-HDI countries. Optimizing perioperative management will potentially improve postoperative outcomes. This should be made a global priority in the field to decrease postoperative mortality, particularly in low-to-middle-HDI countries.

Postoperative death following a treatable complication has emerged as a focus for tackling inequalities in general surgical care in the past decade through the depiction of global surgery as a global health field^2^. Failure to rescue is defined by death after a treatable complication, and can be used as a measure of preventable deaths^33^. Here, death after pancreatic surgery in low-to-middle-HDI countries was found to be associated with higher failure-to-rescue rates. Although approximately two-thirds of patients experienced a complication of any severity, these were mostly low grade requiring no intervention or drug treatment only, among which pancreatic fistula, infection, and delayed gastric emptying were the most frequent. Although these are well recognized complications of pancreatic surgery, they can vary greatly in severity. Modifiable factors involved in the early recognition and management of complications after pancreatic surgery may affect failure-to-rescue rates. These factors involve the wider surgical ecosystem, including infrastructure together with hospital and governance workforce, which also played a role in the reduction of mortality observed after specialization^34^. Identifying the specific modifiable drivers of postoperative failure to rescue in pancreatic surgery and their management related to mortality ought to be the next priority in the field. In response to this, the IHPBA together with the PancreasGroup.org investigators have committed to collaborate further to face the global inequalities in pancreatic surgery.

The main strengths of this study are its wide geographical capture and richness of technique-specific and outcome data. Over 350 centres performing pancreatic surgery from low- to very high-HDI countries across all continents took part. This not only makes it the largest prospective global study looking at outcomes of pancreatic surgery, but also provides a focus on data from practice that is under-reported in the literature. The information provided in this study may contribute to identifying the reasons underlying complications, which could in turn lead to improved site-specific perioperative management guidelines. Details have been reported from preoperative management, including neoadjuvant chemotherapy, to postoperative care through to 90-day outcomes in this study. This wealth of data points to specific areas in the care of patients undergoing pancreatic surgery that need further research and/or dissemination of information. Another important strength is the prospective design of this study. A snapshot representative of current global practice has been captured. Although a recruitment time frame of 3 months may appear brief and at risk of selection bias owing to seasonal variations, this study design was chosen to provide favourable conditions for the participation of centres in low-HDI countries. In requiring participating centres to include all consecutive pancreatic procedures during the chosen time frame, the authors also hoped to mitigate selective reporting.

Inherent limitations of this study relate to its global collaborative nature, and include the surveillance of prospective data reporting, adherence to the study protocol, and interpretation of data required for each patient. Participating centres were asked to follow the instructions laid out by PancreasGroup.org that included local data validation, but no independent monitoring was possible because of the scale of the study. This is, however, a recognized and accepted limitation of global surgery studies^35,36^. Online platforms were created to provide local investigators with adequate support to carry out the study. The PancreasGroup.org platform and electronic CRF included score calculators, unit convertors, and definitions to ensure uniformity of data capture. Although global recruitment, from all HDI countries, was encouraged, participation from very high-HDI countries was greatest. This may reflect a higher prevalence of pancreatic surgery practice in very high-HDI countries. Participation of certain institutions that initially registered their interest with PancreasGroup.org may also have been affected by the COVID-19 pandemic, or this may represent a true selection bias. Similarly, owing to the relatively smaller number of centres and patients submitted from low- and medium-HDI countries, such patients were grouped into one instead of two groups for the purpose of statistical analysis.

A potential limitation of this study pertains to the introduction and use of the complexity of pancreatic surgery score. Even though this score was formulated based on consistent clinical observations and analyses, it is currently in its inaugural phase of presentation in the literature. The parameters selected might still represent a subset of potential factors influencing outcomes. Although this data set encompasses wide geographical and institutional diversity, the authors recognize the need for external validation beyond the present study cohort. Plans are under way to carry out further validation of this score in non-participating institutions to better comprehend its robustness and generalizability.

Lastly, a perceived limitation of this study lies in the inherent variation in expertise across the centres that contributed data globally. Given the diverse range of institutions involved, from highly specialized centres with vast experience in pancreatic surgery to potentially less experienced regional hospitals, this study has demonstrated significant differences in surgical techniques, perioperative care, and postoperative management. These variations influenced the overall morbidity and mortality rates reported. Although this study has provided a comprehensive global perspective on outcomes after pancreatic surgery, it is essential to recognize that the benchmarks established by highly specialized centres might not be directly comparable to the broad range of outcomes observed in this study. However, the authors consider this a strength of this study as it captured the true morbidity and mortality of pancreatic surgery globally.

In conclusion, this is the first global study on pancreas surgery. Failure to rescue stands out as a key factor impacting the high postoperative mortality rates in low to middle HDI countries after pancreas surgery. Focusing on the heterogeneity in surgical approach, techniques, and postoperative management that we present, is a starting point to identify the key modifiable factors that drive failure to rescue. Further research is needed to characterize these modifiable risk factors. International Hepato-Pancreato-Biliary Association (IHPBA) and PancreasGroup.org collaboration task force will work together with the aim to tackle failure to rescue after pancreatic surgery worldwide.

## Supplementary Material

znad330_Supplementary_Data

## Data Availability

Availability of data for secondary analysis is subject to approval by the Scientific and Management Committees. All requests will be evaluated based on the quality and validity of the proposed project, with decisions reached by majority consensus.
