# Supplementary material for: Pancreatic surgery outcomes: multicentre prospective snapshot study in 67 countries
Source: Br J Surg. 2023 Nov 9;111(1):znad330. doi: 10.1093/bjs/znad330 (PMC10771125; doi:10.1093/bjs/znad330)
Supplement: znad330_Supplementary_Data [file znad330_supplementary_data.docx]

**Pancreas surgery outcomes: A multicenter, prospective snapshot study in 67 countries**

*The PancreasGroup.org Collaborative^1,2,3^* (Appendix)

^1^Department of HPB Surgery and Liver Transplant, Royal Free Hospital NHS Foundation Trust, London, UK.

^2^Division of Surgical and Interventional Science, University College London, UK.

^3^Organ Transplant Center of Excellence, King Faisal Specialist Hospital & Research Centre, Riyadh, SA

**Corresponding author:**

Professor Giuseppe Kito Fusai, MS FRCS

Department of HPB Surgery and Liver Transplant

Royal Free London NHS Foundation Trust

Pond Street, London, NW3 2QG

Phone: +442077940500

Email: g.fusai@ucl.ac.uk

**Corresponding author and address requests for reprints to:**

Dimitri A Raptis MD, PhD

Organ Transplant Center of Excellence

King Faisal Specialist Hospital & Research Centre

7790, 2602, Al Maather, Riyadh 12713

Phone: +447584560889

Email: dimitri.raptis@gmail.com

**Supplementary Materials - Index**

| **Supplementary Tables and Figures** |  |
| --- | --- |
| **Supplemental table 1.** STROBE checklist of items that should be included in reports of cross-sectional studies | *page 3* |
| **Supplemental table 2**. Patient and disease characteristics | *page 5* |
| **Supplemental table 3**. Characteristics for pancreaticoduodenectomies and distal pancreatectomies | *page 6* |
| **Supplemental Table 4**. Characteristics of patients among the 3 Human Development Index Groups | *page 7* |
| **Supplemental Figure 1**. World map of centers in countries (light green) registered to participate in the PancreasGroup.org - International Pancreas Surgery Outcomes Study | *page 8* |
| **Supplemental Figure 2**. World map of centers in countries (dark green) that participated with valid cases in the PancreasGroup.org - International Pancreas Surgery Outcomes Study | *page 8* |
| **Supplemental Figure 3**. Flow chart of patients included in the PancreasGroup.org - International Pancreas Surgery Outcomes Study | *page 9* |
| **Supplemental Figure 4**. Multivariable binary logistic analysis assessing the newly developed complexity of pancreatic surgery score in relation to 90-day mortality. | *page 9* |
| **Supplementary Appendixes** |  |
| **Appendix** – Group Authorship - Committees | *page 10* |
| **Appendix** – Group Authorship – Members | *page 13* |

**Supplemental tables**

**Supplemental table 1.** STROBE checklist of items that should be included in reports of ***cross-sectional studies***

|  | Item No | Recommendation | Check |
| --- | --- | --- | --- |
| **Title and abstract** | 1 | (*a*) Indicate the study’s design with a commonly used term in the title or the abstract | ✔ |
|  |  | (*b*) Provide in the abstract an informative and balanced summary of what was done and what was found | ✔ |
| Introduction | | |  |
| Background/rationale | 2 | Explain the scientific background and rationale for the investigation being reported | ✔ |
| Objectives | 3 | State specific objectives, including any prespecified hypotheses | ✔ |
| Methods | | |  |
| Study design | 4 | Present key elements of study design early in the paper | ✔ |
| Setting | 5 | Describe the setting, locations, and relevant dates, including periods of recruitment, exposure, follow-up, and data collection | ✔ |
| Participants | 6 | (*a*) Give the eligibility criteria, and the sources and methods of selection of participants | ✔ |
| Variables | 7 | Clearly define all outcomes, exposures, predictors, potential confounders, and effect modifiers. Give diagnostic criteria, if applicable | ✔ |
| Data sources/ measurement | 8* | For each variable of interest, give sources of data and details of methods of assessment (measurement). Describe comparability of assessment methods if there is more than one group | ✔ |
| Bias | 9 | Describe any efforts to address potential sources of bias | ✔ |
| Study size | 10 | Explain how the study size was arrived at | ✔ |
| Quantitative variables | 11 | Explain how quantitative variables were handled in the analyses. If applicable, describe which groupings were chosen and why | ✔ |
| Statistical methods | 12 | (*a*) Describe all statistical methods, including those used to control for confounding | ✔ |
|  |  | (*b*) Describe any methods used to examine subgroups and interactions | ✔ |
|  |  | (*c*) Explain how missing data were addressed | ✔ |
|  |  | (*d*) If applicable, describe analytical methods taking account of sampling strategy | ✔ |
|  |  | (*e*) Describe any sensitivity analyses | ✔ |
| Results | | |  |
| Participants | 13* | (a) Report numbers of individuals at each stage of study—eg numbers potentially eligible, examined for eligibility, confirmed eligible, included in the study, completing follow-up, and analysed | ✔ |
|  |  | (b) Give reasons for non-participation at each stage | ✔ |
|  |  | (c) Consider use of a flow diagram | ✔ |
| Descriptive data | 14* | (a) Give characteristics of study participants (eg demographic, clinical, social) and information on exposures and potential confounders | ✔ |
|  |  | (b) Indicate number of participants with missing data for each variable of interest | ✔ |
| Outcome data | 15* | Report numbers of outcome events or summary measures | ✔ |
| Main results | 16 | (*a*) Give unadjusted estimates and, if applicable, confounder-adjusted estimates and their precision (eg, 95% confidence interval). Make clear which confounders were adjusted for and why they were included | ✔ |
|  |  | (*b*) Report category boundaries when continuous variables were categorized | ✔ |
|  |  | (*c*) If relevant, consider translating estimates of relative risk into absolute risk for a meaningful time period | ✔ |
| Other analyses | 17 | Report other analyses done—eg analyses of subgroups and interactions, and sensitivity analyses | ✔ |
| Discussion | | |  |
| Key results | 18 | Summarise key results with reference to study objectives | ✔ |
| Limitations | 19 | Discuss limitations of the study, taking into account sources of potential bias or imprecision. Discuss both direction and magnitude of any potential bias | ✔ |
| Interpretation | 20 | Give a cautious overall interpretation of results considering objectives, limitations, multiplicity of analyses, results from similar studies, and other relevant evidence | ✔ |
| Generalisability | 21 | Discuss the generalisability (external validity) of the study results | ✔ |
| Other information | | |  |
| Funding | 22 | Give the source of funding and the role of the funders for the present study and, if applicable, for the original study on which the present article is based | ✔ |

**Supplemental table 2**. Patient and disease characteristics

| **Parameters** | **Values** | |
| --- | --- | --- |
| **Patient Characteristics** |  | |
| Age, median (IQR) | 64 (55-72) | |
| Female gender, n (%) | 2032 (48.2) | |
| BMI kg/m2, median (IQR) | 24.7 (22.0-27.7) | |
| ASA status, n (%) |  | |
| ASA 1 | 665 (15.8) | |
| ASA 2 | 2129 (50.4) | |
| ASA 3 | 1349 32.0) | |
| ASA 4 | 79 (1.9) | |
| **Comorbidities, n (%)** |  | |
| Cardiac disease | 789 (18.7) | |
| Diabetes mellitus | 1146 (27.1) | |
| Diet controlled | 181 (4.3) | |
| Oral antidiabetics | 649 (15.3) | |
| Insulin administration | 422 (10) | |
| Chronic kidney disease | 133 (3.2) | |
| Metastatic cancer | 74 (1.8) | |
| Cirrhosis | 52 (1.2) | |
| Stroke | 104 (2.5) | |
| COPD | 212 (5.0) | |
| Asthma | 176 (4.2) | |
| Other | 1568 (37) | |
| Pancreatic enzyme supplementation | 386 (9.9) | |
| COVID-19 Status |  | |
| Previous diagnosis | 660 (15.6) | |
| Diagnosed >4 weeks preoperatively | 310 (7.3) | |
| Current diagnosis | 32 (0.75) | |
| Unknown / not tested | 271 (6.4) | |
| **Disease Characteristics, n (%)** |  | |
| Ampullary adenoma | 40 (1.0) |  |
| Ampullary carcinoma | 417 (9.9) |  |
| Benign cystic lesions | 246 (5.9) |  |
| Cholangiocarcinoma | 239 (5.7) |  |
| Chronic pancreatitis | 320 (7.6) |  |
| Colorectal cancer metastasis | 24 (0.6) |  |
| Duodenal adenocarcinoma | 117 (2.8) |  |
| Gastric adenocarcinoma | 13 (0.3) |  |
| GIST | 14 (0.3) |  |
| Intraductal papillary mucinous neoplasm | 254 (6.1) |  |
| Neuroendocrine neoplasia | 395 (9.4) |  |
| Pancreatic acinar cell carcinoma | 14 (0.3) |  |
| Pancreatic ductal adenocarcinoma | 1894 (45.1) |  |
| Renal clear cell metastasis | 34 (0.8) |  |
| Sarcoma | 21 (0.5) |  |
| Solid pseudopapillary tumor | 65 (1.5) |  |
| Other | 90 (2.1) |  |

**Supplemental table 3.** Characteristics for pancreaticoduodenectomies and distal pancreatectomies

| **Parameters** | **Pancreaticoduodenectomy** | **Distal pancreatectomy** |
| --- | --- | --- |
| ***Preoperative blood values*** | *Median, IQR, n=2544* | *Median, IQR, n=1043* |
| Hemoglobin d/L | 121 (105-135) | 128 (113-140) |
| Creatinine μmol/L | 70 (59-85) | 70 (60-85) |
| Albumin g/L | 39 (34-42) | 42 (38-45) |
| Bilirubin μmol/L | 17 (8-56) | 8 (5-12) |
|  |  |  |
| ***Preoperative stent*** | *n, (%), n=2540* | *N/A* |
| Preoperative stent | 1210 (47.6) | - |
| ERCP | 1049 (41.3) | - |
| PTBD after failed ERCP | 56 (2.2) | - |
| PTBD | 105 (4.1) | - |
|  |  |  |
| ***Surgical approach*** | *n, (%), n=2544* | *n, (%), n=1043* |
| Open | 2428 (95.4) | 662 (63.5) |
| Minimally invasive | 116 (4.6) | 381 (36.5) |
| Laparoscopic | 61 (2.4) | 280 (26.8) |
| Converted | 41 (1.6) | 53 (5.1) |
| Robotic | 55 (2.2) | 101 (9.7) |
| Converted | 9 (0.4) | 13 (1.2) |
|  |  |  |
| ***Intraoperative findings*** | *n, (%), n=2272* | *n, (%), n=803* |
| Texture of pancreas |  |  |
| Soft / Normal | 1285 (55.9) | 588 (73.2) |
| Hard / Fibrotic | 1013 (44.1) | 215 (26.8) |
| Size of pancreatic duct |  | N/A |
| <3mm | 728 (32.0) | - |
| 3-8mm | 1393 (61.3) | - |
| >8mm | 151 (6.6) | - |
|  |  |  |
| ***Pancreatic anastomosis*** | *n, (%), n=2542* | *N/A* |
| Pancreaticojejunostomy | 2241 (88.2) | - |
| Pancreaticogastrostomy | 258 (10.1) | - |
|  |  |  |
| ***Closure of pancreatic stump in 56 various combinations*** | *N/A* | *n, (%), n=1033* |
| Hand-Sewn | - | 268 (25.8) |
| Stapler | - | 542 (52.2) |
| Reinforced staple line | - | 175 (16.9) |
| Separate ligation of the pancreatic duct | - | 103 (9.9) |
| Pancreaticoenteric anastomosis | - | 11 (1.1) |
| Seromuscular patch | - | 9 (0.9) |
| Ligament patch | - | 45 (4.4) |
| Fibrin glue sealing | - | 43 (4.2) |
| Surface active meshe application | - | 75 (7.3) |
| Other | - | 27 (2.6) |
|  |  |  |
|  |  |  |
|  |  |  |

**Supplemental Table 4**. Characteristics of patients among the 3 Human Development Index Groups

| **Parameters** | **Low to Medium HDI** | **High HDI** | **Very High HDI** | ***P* value** |
| --- | --- | --- | --- | --- |
|  | *n=285* | *n=494* | *n=3444* |  |
| Age, median (IQR) | 56 (45-65) | 59 (49-67) | 66 (57-73) | **<0.001** |
| Female gender, n (%) | 133 (47) | 231 (47) | 1668 (49) | 0.679 |
| BMI kg/m2, median (IQR) | 23 (19-26) | 25 (22-28) | 25 (22-28) | **<0.001** |
| ASA status > 2, n (%) | 51 (18) | 60 (12) | 1337 (38) | **<0.001** |
| Malignancy, n (%) | 197 (69) | 396 (80) | 2707 (79) | **0.001** |
| Minimally invasive approach, n (%) | 29 (10) | 36 (7) | 485 (14) | **<0.001** |
| Operation duration in min., median (IQR) | 320 (240-398) | 300 (210-420) | 320 (240-420) | **0.017** |
| Extended procedure to additional organs, n (%) | 40 (14) | 83 (17) | 476 (14) | 0.212 |
| Portomesenteric resection, n (%) | 11 (4) | 79 (16) | 414 (12) | **<0.001** |
| Arterial resection, n (%) | 35 (7) | 12 (4) | 72 (2) | **<0.001** |
| Complexity of pancreatic surgery score, mean(SD) | 1.8 (1.0) | 2.1 (1.0) | 2.1 (1.0) | **<0.001** |
| Complication of any severity, 90 day | 200 (70) | 304 (62) | 2389 (70) | **0.001** |
| Grade ≥3a complication, 90 day | 69 (24) | 89 (18) | 932 (27) | **<0.001** |
| Grade ≥3b complication, 90 day | 41 (14) | 53 (11) | 535 (16) | **0.019** |
| CCI*^®^* until discharge, median (IQR) | 9 (0-30 | 9 (0-23) | 9 (0-30) | **<0.001** |
| CCI*^®^* until 90 days postoperatively, median (IQR) | 17 (0-34) | 9 (0-26) | 21 (0-34) | **<0.001** |
| IMC/HDU stay, median (IQR) | 1 (0-3) | 0 (0-3) | 0 (0-1) | **<0.001** |
| Intensive care unit stay, median (IQR) | 1 (0-2) | 2 (0-3) | 1 (0-2) | 0.151 |
| Hospital stay in days, median (IQR) | 9 (6-14) | 9 (6-15) | 11 (8-18) | **<0.001** |
| Hospital readmission rate until 90 days, n (%) | 25 (12) | 66 (17) | 632 (21) | **0.003** |
| Mortality rate, 90 day, n (%) | 28 (10) | 24 (5) | 177 (5) | **0.003** |
| Failure-to-rescue rate, n (%) | 28/69 (41) | 24/89 (27) | 177/3444 (5) | **<0.001** |

**Supplemental figures**


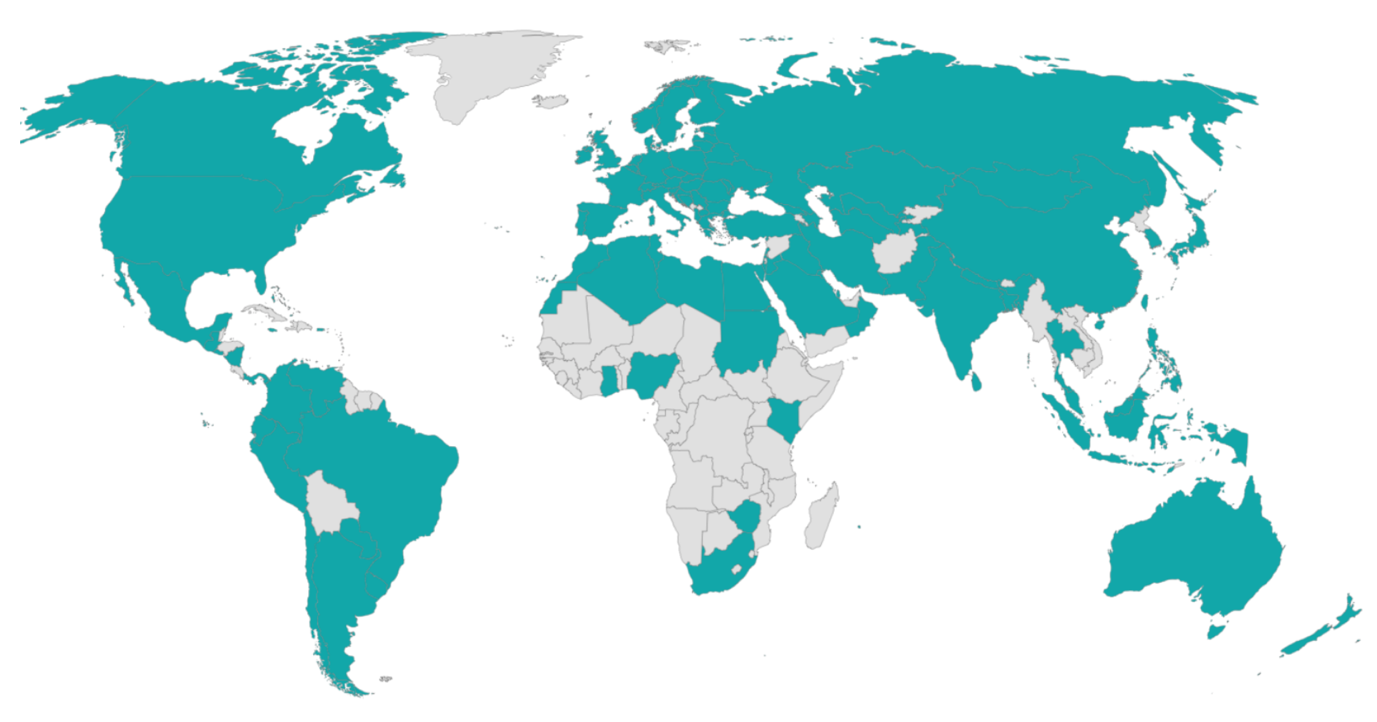


**Supplemental Figure 1.** World map of centers in countries (light green) registered to participate in the PancreasGroup.org - International Pancreas Surgery Outcomes Study


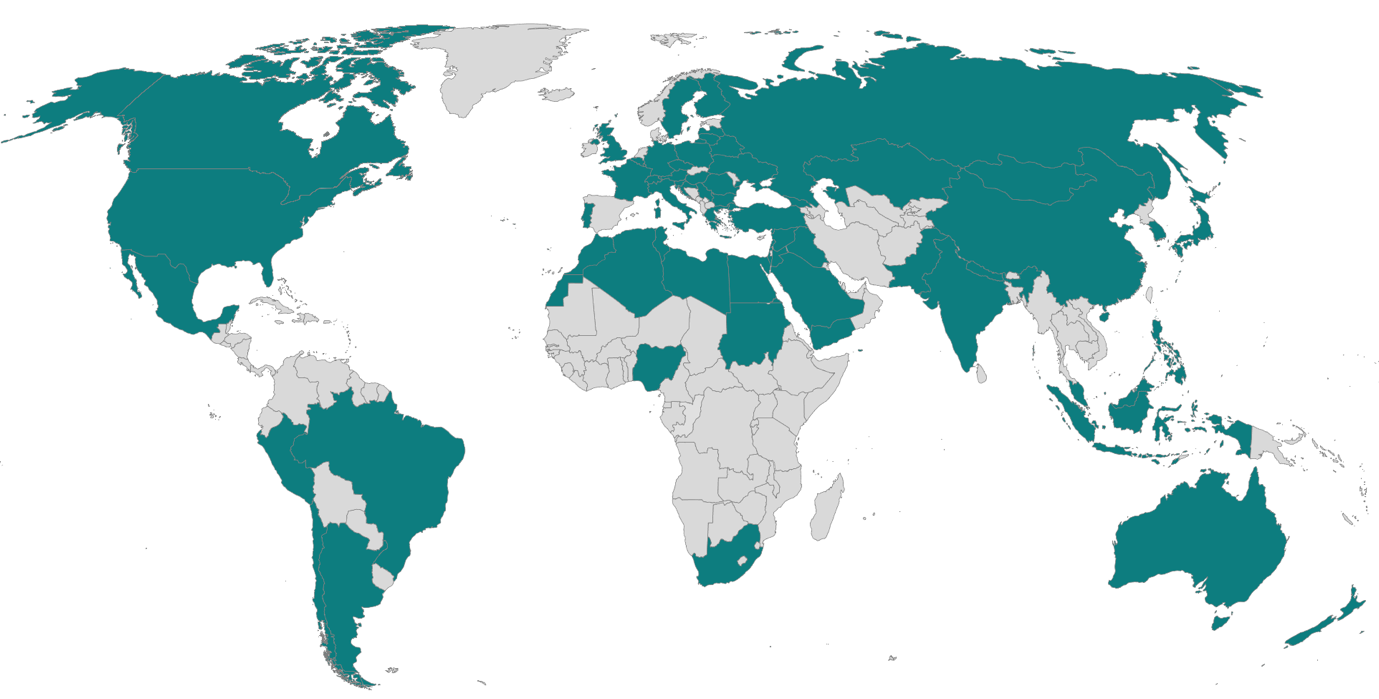


**Supplemental Figure 2.** World map of centers in countries (dark green) that participated with valid cases in the PancreasGroup.org - International Pancreas Surgery Outcomes Study


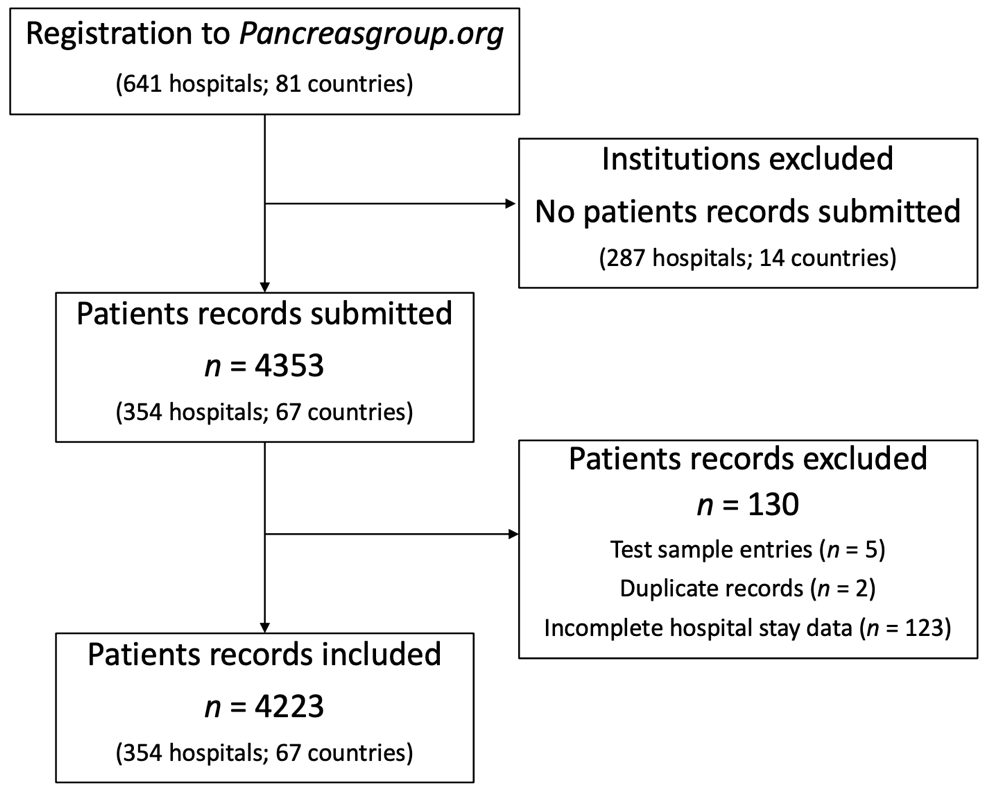


**Supplemental Figure 3.** Flow chart of patients included in the PancreasGroup.org - International Pancreas Surgery Outcomes Study


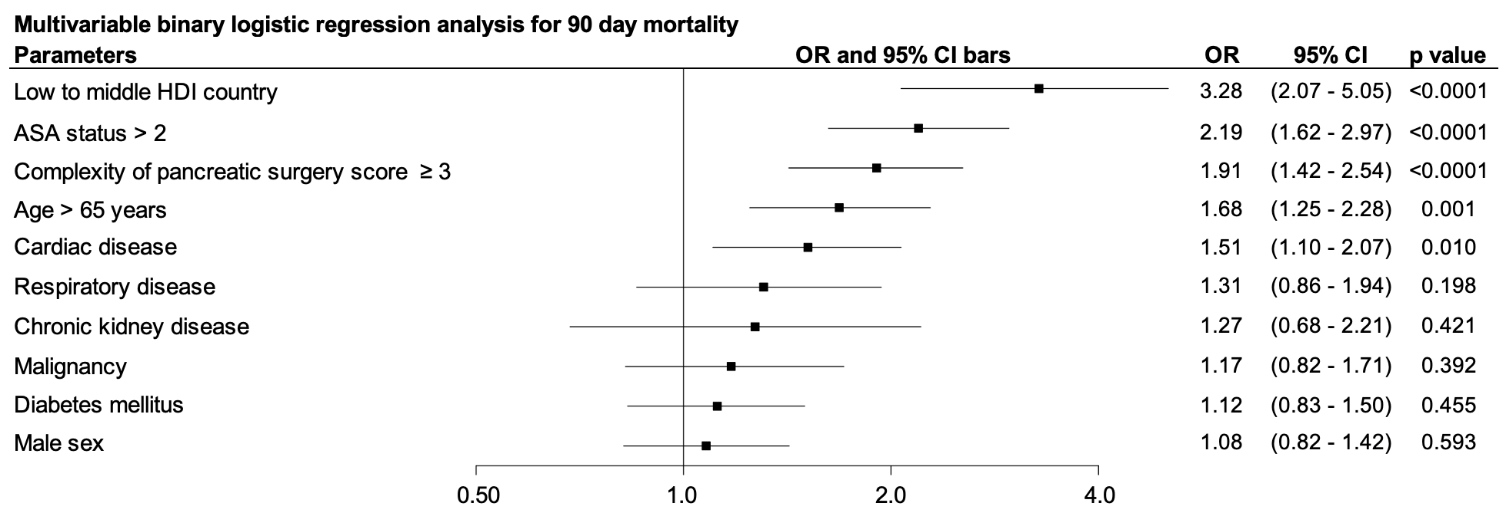


**Supplemental Figure 4.** Multivariable binary logistic analysis assessing the newly developed complexity of pancreatic surgery score in relation to 90-day mortality.

**Appendix – Group Authorship**

**PancreasGroup.org Committees**

**Chief Investigators:** Giuseppe Kito Fusai, Royal Free Hospital & University College London, UK. Cristina_Ferrone, Cedars-Sinai Medical Centerm, Los Angeles, California, USA.

**Co-Chief Investigator:** Dimitri Aristotle Raptis, Organ Transplant Center of Excellence

King Faisal Specialist Hospital & Research Centre, Riyadh, Saudi Arabia.

**Scientific Committee:**

Mohamed Abu Hilal, University Hospital Southampton, UK. Claudio Bassi, University Hospital of Verona, Italy. Marc Besselink, University of Amsterdam, Netherlands. Kevin Conlon, Trinity College Dublin, Ireland. Brian Davidson, University College London, UK. Marco Del Chiaro, University of Colorado Anschutz Medical Campus, USA. Christos Dervenis, Metropolitan Hospital Athens, Greece. Isabella Frigerio, Pederzoli Clinic, Verona, Italy. Massimo Falconi, San Raffaele Hospital, Milano, Italy. Thilo Hackert, University of Heidelberg, Germany. Ewen M. Harrison, University of Edinburgh, UK. Shailesh V. Shrikhande, Tata Memorial Hospital, Mumbai, India. Ajith Siriwardena, Manchester University, UK. Martin Smith, University of the Witwatersrand, Johannesburg, South Africa. Christopher Wolfgang, Johns Hopkins Hospital, Maryland, USA

**Management Committee**:

Aditya Borakati, Royal Free Hospital, London, UK. Deniz Balci, Ankara University Hospital, Turkey. Muhammed Elhadi, University of Tripoli, Libya. Camila Hidalgo Salinas, Royal Free Hospital, London, UK. Nikolaos Machairas, Royal Free Hospital, London, UK. Giovanni Marchegiani, University Hospital of Verona, Italy. Atsushi Oba, University of Colorado Anschutz Medical Campus, USA. Christian Oberkofler, University Hospital Zurich, Switzerland. Ioannis Passas, Metropolitan Hospital Athens, Greece. Reena Ravikumar, Royal Infirmary of Edinburgh, UK. Patricia Sánchez Velázquez, University Hospital del Mar, Barcelona, Spain. Martin de Santibañes, Italian Hospital of Buenos Aires, Argentina. Andreas Anton Schnitzbauer, University Hospital Frankfurt, Germany. Fiammetta Soggiu, Royal Free Hospital, London, UK. Domenico Tamburrino, San Raffaele Hospital, Milano, Italy. Pascale Tinguely, Royal Free Hospital, London, UK. Alice Wei, Memorial Sloan Kettering Cancer, New York, USA. Marinos Zachiotis, University of Patras, Greece.

**Country Leaders:**

**Algeria:** Kamel Bentabak, Pierre and Marie Curie Cancer Center, Algiers; Salah Eddine Kacimi, Faculty of Medicine, University of Tlemcen Tlemcen. **Australia:** Mehrdad Nikfarjam, University of Melbourne, Melbourne. **Belarus**: Aliaksei Shcherba, Minsk medical Center for Surgery, Transplantation and Hematology Minsk. **Belgium:** Gregory Sergeant, Jessa Ziekenhuis Hasselt. **Brazil:** Gustavo Coelho, Federal University of Ceara Fortaleza. Orlando Torres, Maranhão Federal University Sao Luis. **Bulgaria**: Nikolay Belev, UMHAT-"Eurohospital", Medical Training and Simulation Centre, MU-Plovdiv; Burundi Fabrice, Muhezagiro University of Burundi, Bujumbura. **Canada:** Ephraim Tang, London Health Sciences Centre - Western University London, Ontario; Janet Martin, Western University London. **Chile**: Christian Diaz, Hospital Dr Sotero del Rio, Santiago; Nicolas Devaud Instituto Oncologico Fundacion Arturo Lopez Perez (FALP) Santiago. **China**: Kongyuan Wei, Heidelberg University Hospital/ Chinese PLA General Hospital, Beijing; Maher Hendi, Zhejiang University School of Medicine, Sir Ren Ren Shaw Hospital, Hangzhou. **Croatia**: Danko Mikulic University Hospital Merkur Zagreb. **Cyprus**: Nikolaos Gouvas, University of Cyprus, Nicosia. Thalis Christophides, Nicosia General Hospital. **Czech Republic**: Andrej Nikov, University Hospital Kralovske Vinohrady, Prague. **Egypt**: Dalia Fathallah, Alexandria University Main Hospital, Alexandria; Mahmoud Saad, Assiut Faculty of Medicine, Assiut. **Estonia**: Olav Tammik, University Clinic of Tartu, Tartu. **Finland:** Heikki Huhta, Oulu University Hospital, Oulu. **France:** Laurent Sulpice, University Hospital Rennes, Rennes; Renato Lupinacci, Ambroise Pare Hospital, Paris-Saclay University, Paris. **Georgia**: Zaza Demetrashvili, Tbilisi State Medical University, Tbilisi. **Germany**: Gregor A. Stavrou, Klinikum Saarbrücken. **Greece** Evangelos Felekouras, National and Kapodistrian University of Athens; Vasileios Papaziogas, Aristotle University of Thessaloniki. **India**: Sanjeev Misra, All India Institute of Medical Sciences Jodhpur. **Indonesia**: Erik Prabowo Diponegoro University / Kariadi General Hospital, Semarang. **Iraq**: Hashim Talib Hashim University of Baghdad, College of Medicine, Baghdad; Maytham Al-Juaifari Al-Sader, Teaching Hospital, An Najaf. **Japan**: Sohei Satoi, Kansai Medical University Hirakata. **Jordan**: Khaled Obeidat, Jordan University of Science and Technology, Irbid; Maram Mohsen, Jordan University Hospital, Amman. **Kazakhstan**: Ildar Fakhradiyev, Republic of **Korea**: Ho-Seong Han, Seoul National University Bundang Hospital, Seongnam. **Lebanon**: Mohamad Khalife, American University of Beirut. **Libya**: Muhammed Elhadi, Faculty of Medicine, University of Tripoli. **Lithuania**: Audrius Dulskas, National Cancer Institute Vilnius. **Malaysia**: Jin Bong Sunway Medical Center Petaling Jaya. **Maldives:** Shahi Ghani, Tree Top Hospital, Male'. Mexico: Alejandro Eduardo Padilla, Rosciano National Cáncer Institute, Mexico City; Javier Melchor-Ruan, National Cancer Institute Mexico. **Mongolia**: Sarnai Erdene, Mongolian National University of Medical Sciences, Ulaanbaatar. **Morocco**: Amine Benkabbou, National Institute of Oncology, Mohammed V University in Rabat. **Namibia**: Pueya Nashidengo, Windhoek Central Academic Hospital/University of Namibia School of Medicine, Windhoek. **New Zealand**: Jonathan Koea, North Shore Hospital, Auckland. **Nigeria** Ademola Adeyeye, Afe Babalola University Ado-Ekiti(ABUAD) Ado-Ekiti; Olusegun Alatise, Obafemi Awolowo University, Ile Ife. **Pakistan:** Sami Ullah Services Hospital Lahore Lahore. Palestine, Mustafa Abu Jayyab Alshifaa Hospital, Gaza; Sarah Amro, Alia Govermental Hospital, Hebron; Walaa Mohammed Alnammourah, Alia Governmental Hospital, Hebron. **Philippines**: Catherine The, Makati Medical Center, Makati. **Poland**: Michał Pędziwiatr, Jagiellonian University Medical College, Kraków; Wojciech Polkowski, Medical University of Lublin. **Romania**: Sorin Traian Barbu, "Iuliu Hatieganu" University of Medicine & Pharmacy, Cluj-Napoca **Serbia**: Aleksandar Karamarkovic, Surgical Clinic "Nikola Spasic", University Clinical Center "Zvezdara", Belgrade, Faculty of Medicine University of Belgrade; Daniel Galun, Clinic for Digestive Surgery, Clinical Center of Serbia, Belgrade. **Singapore**: Brian K. P. Goh, Singapore General Hospital, Singapore. **Slovenia**: Blaž Trotovšek, University Medical Centre Ljubljana. **South** **Africa**: Jones Omoshoro-Jones, Chris Hani Baragwanath Academic Hospital/University of the Witwatersrand, Johannesburg. **Spain**: Benedetto Ielpo, Hospital del Mar, Barcelona. **Sudan**: Abdelfatah Abdelmageed, Omdurman Islamic University, Khartoum. **Sweden**: Per Sandström, University Hospital of Linkoping. **Switzerland**: Alessandra Cristaudi, Ospedale Regionale di Lugano; Beat Gloor, Inselspital, University Bern; Christoph Kuemmerli, Clarunis, University Centre for Gastrointestinal and Liver Diseases, St. Clara Hospital and University Hospital, Basel. **Syria**: Alaa Hamdan Tishreen, University Hospital Lattakia; Mohammad Karam Chaaban Al-Mouwasat University Hospital, Damascus. **Taiwan**: Chien Hui Wu, National Taiwan University Hospital, Taipei; Po-Chih Yang Fu Jen, Catholic University Hospital, New Taipei City. **Tunisia**: Ammar Houssem Sahloul Hospital, Sousse; Oussama Baraket, Hopital Habib Bouguetfa de Bizerte, University Tunis El Labar Bizerte. **Turkey**: Ahmet Çoker Ege University, Izmir HPB Clinic, Izmir. **United Kingdom**: Mark Taylor, Belfast Health and Social Care Trust, Belfast; Nigel Jamieson, University of Glasgow; Satheesh Iype, Royal Free Hospital London. **United States of America**: Emmanouil Giorgakis, UAMS Medical Center/ Winthrop P Rockefeller Cancer Institute, Little Rock; Motaz Qadan, Massachusetts General Hospital, Boston; Sabha Ganai, University of North Dakota, Sanford Health, Fargo. **Yemen**: Hamza Al-Naggar, Faculty of Medicine - Sana'a University, Sana'a. **Zimbabwe**: Onesai Chihaka, University of Zimbabwe, Harare.

**PancreasGroup.org Members:**

*Please see next page…*

| **First name(s)** | **Last name** | **Institution** | **City** | **Country** |
| --- | --- | --- | --- | --- |
| Amor | El Behi | University Of Sousse | Alger | Algeria |
| Aya Tinhinane | Kouicem | Université Ferhat Abbas 1 | Algerie | Algeria |
| Aissam | Chibane | Pierre And Marie Curie Cancer Center | Algiers | Algeria |
| Chafik | Bouzid | Pierre And Marie Curie Cancer Center | Algiers | Algeria |
| Kamel | Bentabak | Pierre And Marie Curie Cancer Center | Algiers | Algeria |
| Imene | Bouali | Faculty Of Medicine - Batna | Batna | Algeria |
| Noureddine | Samai | Faculty Of Medicine - Batna | Batna | Algeria |
| Boudouh | Aya | University Of Batna 2 | Batna | Algeria |
| Boutheyna | Drid | University Of Batna 2 | Batna | Algeria |
| Anisse | Tidjane | University Oran 1 | Oran | Algeria |
| Benali | Tabeti | University Oran 1 | Oran | Algeria |
| Nabil | Boudjenan-Serradj | University Oran 1 | Oran | Algeria |
| Mohammed Hakim | Larbi | University Oran 1 | Oran | Algeria |
| Ilhem | Ouahab | Ferhat Abbas University | Setif | Algeria |
| Souhem | Touabti | Medical School | Setif | Algeria |
| Ouahab | Ilhem | Chu Saadna Abdenour Sétif | Sétif | Algeria |
| Souad | Bouaoud | Chu Saadna Abdenour Sétif | Sétif | Algeria |
| Abdoun | Meriem | Ferhat Abbas University | Sétif | Algeria |
| Amel | Ouyahia | Ferhat Abbas University | Sétif | Algeria |
| Aya | Tinhinane Kouicem | Ferhat Abbas University | Sétif | Algeria |
| Meriem | Abdoun | Ferhat Abbas University | Sétif | Algeria |
| Rais | Mounira | Ferhat Abbas University | Sétif | Algeria |
| Mounira | Rais | Saadna Abdennour University Hospital | Sétif | Algeria |
| Omar | Riffi | Abu Bakr Belkaid University | Tlemcen | Algeria |
| Salah Eddine | Kacimi | Abu Bakr Belkaid University | Tlemcen | Algeria |
| Lucas | McCormack | Hospital Aleman Of Buenos Aires | Buenos Aires | Argentina |
| Pablo | Capitanich | Hospital Aleman Of Buenos Aires | Buenos Aires | Argentina |
| Jeremias | Goransky | Hospital Italiano De Buenos Aires | Buenos Aires | Argentina |
| Martin | de Santibanes | Hospital Italiano De Buenos Aires | Buenos Aires | Argentina |
| Oscar | Mazza | Hospital Italiano De Buenos Aires | Buenos Aires | Argentina |
| Ivana | Pedraza Salazar | Instituto Alexander Fleming | Buenos Aires | Argentina |
| Dario Roberto | Ramallo | Hospital Aleman Of Buenos Aires | Buenso Aires | Argentina |
| Farinelli | Pablo | Universitary Hospital Favaloro Foundation | Buenso Aires | Argentina |
| Gabriel E. | Gondolesi | Universitary Hospital Favaloro Foundation | Buenso Aires | Argentina |
| Pablo | Barros Schelotto | Universitary Hospital Favaloro Foundation | Buenso Aires | Argentina |
| Jorge | Rodriguez | University of La Plata | La Plata | Argentina |
| Christos | Apostolou | Sydney Adventist Hospital, Australian National University | Sydney | Australia |
| Neil | Merrett | Western Sydney University, Bankstown Hospital | Bankstown | Australia |
| Adrian | Fox | Box Hill Hospital | Burwood | Australia |
| Sayed | Hassen | Box Hill Hospital | Burwood | Australia |
| Shantanu | Joglekar | Box Hill Hospital | Burwood | Australia |
| Sivakumar | Gananadha | Canberra Hospital | Canberra | Australia |
| Rudyard | Wake | Canberra Hospital | Canberra | Australia |
| Krista | Hagen | Canberra Hospital | Canberra | Australia |
| Mithra | Sritharan | Monash Health | Clayton | Australia |
| Kat | Hall | Austin Health / Knox Private Hospital | Melbourne | Australia |
| Vijayaragavan | Muralidharan | Austin Health / Knox Private Hospital | Melbourne | Australia |
| Kai | Brown | Austin Hospital | Melbourne | Australia |
| Mehrdad | Nikfarjam | Austin Hospital | Melbourne | Australia |
| Daniel | Croagh | Monash Health | Melbourne | Australia |
| Mithra | Sritharan | Monash Health | Melbourne | Australia |
| Roger | Berry | Monash Health | Melbourne | Australia |
| Aly | Fayed | Northern Health | Melbourne | Australia |
| Russell | Hodgson | Northern Health | Melbourne | Australia |
| Thiep | Kuany | Northern Health | Melbourne | Australia |
| Benjamin | Loveday | Royal Melbourne Hospital And Peter Maccallum Cancer Centre | Melbourne | Australia |
| Samuel | Banting | Royal Melbourne Hospital And Peter Maccallum Cancer Centre | Melbourne | Australia |
| Alistair | Rowcroft | St Vincent'S Hospital Melbourne | Melbourne | Australia |
| Adrian | Fox | St Vincent’s Hospital Melbourne | Melbourne | Australia |
| Brett | Knowles | St Vincent’s Hospital Melbourne | Melbourne | Australia |
| Lillian | Taylor | St Vincent’s Hospital Melbourne | Melbourne | Australia |
| Lynn | Chong | St Vincent’s Hospital Melbourne | Melbourne | Australia |
| Simon | Banting | St Vincent’s Hospital Melbourne | Melbourne | Australia |
| Marcos | Perini | The University Of Melbourne | Melbourne | Australia |
| Mehrdad | Nikfarjam | University Of Melbourne | Melbourne | Australia |
| Yi-Ju | Lin | University Of Melbourne | Melbourne | Australia |
| Ali | Alsoudani | John Hunter Hospital | Newcastle | Australia |
| David | Burnett | John Hunter Hospital | Newcastle | Australia |
| Kalpesh | Shah | John Hunter Hospital | Newcastle | Australia |
| Matthew | Fuge | John Hunter Hospital | Newcastle | Australia |
| Nicholas | Bull | John Hunter Hospital | Newcastle | Australia |
| Stanley | Chen | John Hunter Hospital | Newcastle | Australia |
| Suresh | Navadgi | Royal Perth Hospital | Perth | Australia |
| Zi Qin | Ng | Royal Perth Hospital | Perth | Australia |
| Mikael | Johansson | Sir Charles Gairdner Hospital | Perth | Australia |
| Nur Sabrina | Binti Babe Azaman | The University Of Western Australia | Perth | Australia |
| Andrew | Pearson | Sydney Adventist Hospital | Sydney | Australia |
| Christos | Apostolou | Sydney Adventist Hospital | Sydney | Australia |
| Hans | Mischinger | Medical University Graz | Graz | Austria |
| Peter | Schemmer | Medical University Graz | Graz | Austria |
| Peter | Kornprat | Medical University Graz | Graz | Austria |
| Andreas | Hauer | Hospital Horn | Horn | Austria |
| Andreas | Hauer | Hospital Horn | Horn | Austria |
| Klaus | Kirbes | Hospital Horn | Horn | Austria |
| Reinhold | Klug | Hospital Horn | Horn | Austria |
| Rudolf | Schrittwieser | Hospital Leoben | Leoben | Austria |
| Alexander | Klaus | Hospital Sisters Of Mercy | Vienna | Austria |
| Alexandra | Entschev | Hospital Sisters Of Mercy | Vienna | Austria |
| Daniel | Reichhold | Hospital Sisters Of Mercy | Vienna | Austria |
| Keti | Ugrekhelidze | Hospital Sisters Of Mercy | Vienna | Austria |
| Marcus | Fink | Hospital Sisters Of Mercy | Vienna | Austria |
| Radoslava | Stoyanova | Hospital Sisters Of Mercy | Vienna | Austria |
| Marah | Sabateen | Beit- Jala Hospital | Bethlehem | Azerbaijan |
| Zainab | Mahfoodh | Alexandrine Faculty Of Medicine | Alexandrine | Bahrain |
| Hamdi | Al Shenawi | Cmms - Arabian Gulf University | Manama | Bahrain |
| Rami | Yaghan | Cmms - Arabian Gulf University | Manama | Bahrain |
| Mohammad | Chowdhury | Bangabandhu Sheikh Mujib Medical University. Dhaka | Dhaka | Bangladesh |
| Aliaksei | Shcherba | Minsk Medical Center For Surgery, Transplantation And Hematology | Minsk, Belarus | Belarus |
| Leonid | Kirkovsky | Minsk Medical Center For Surgery, Transplantation And Hematology | Minsk, Belarus | Belarus |
| Sergey | Korotkov | Minsk Medical Center For Surgery, Transplantation And Hematology | Minsk, Belarus | Belarus |
| Bert | Van den Bossche | A.S.Z. Aalst - Geraardsbergen - Wetteren | Aalst | Belgium |
| Kim | Boterbergh | A.S.Z. Aalst - Geraardsbergen - Wetteren | Aalst | Belgium |
| Martin | Poortmans | A.S.Z. Aalst - Geraardsbergen - Wetteren | Aalst | Belgium |
| Bart | Smet | Az Delta - Az St. Jan Brugge | Bruges | Belgium |
| Sébastien | Strypstein | Az Delta - Az St. Jan Brugge | Bruges | Belgium |
| Tom | Feryn | Az Delta - Az St. Jan Brugge | Bruges | Belgium |
| El Mahdi | Wahib | Free University Of Brussels | Brussels | Belgium |
| sara | oubella | Free University Of Brussels | Brussels | Belgium |
| Geert | Roeyen | Antwerp University Hospital | Edegem | Belgium |
| Vera | Hartman | Antwerp University Hospital | Edegem | Belgium |
| Bart | Bracke | Antwerp University Hospital | Edegem | Belgium |
| Bart | Hendrikx | University Hospital Ghent | Gent | Belgium |
| Filip | Gryspeerdt | University Hospital Ghent | Gent | Belgium |
| Frederik | Berrevoet | University Hospital Ghent | Gent | Belgium |
| Natalie | Poortmans | University Hospital Ghent | Gent | Belgium |
| Thomas | Apers | University Hospital Ghent | Gent | Belgium |
| Bart | Appeltans | Jessa Ziekenhuis | Hasselt | Belgium |
| Bart | Appeltans | Jessa Ziekenhuis | Hasselt | Belgium |
| Dennis | Wicherts | Jessa Ziekenhuis | Hasselt | Belgium |
| Gregory | Sergeant | Jessa Ziekenhuis | Hasselt | Belgium |
| Fernanda | Oliveira Barreto Garcia | Hospital Universitário João De Barros Barreto | Belém | Brazil |
| Ian | Barroso dos Santos | Hospital Universitário João De Barros Barreto | Belém | Brazil |
| Rafael | Garcia | Hospital Universitário João De Barros Barreto | Belém | Brazil |
| Rinaldo | Pinto | Hospital Santa Catarina De Blumenau | Blumenau | Brazil |
| Thais | Lins Soares Leite | Hospital Santa Catarina De Blumenau | Blumenau | Brazil |
| Marciano | Anghinoni | Centro De Oncologia Do Paraná | Curitiba | Brazil |
| Caroline | Celestino Girão Nobre | Federal University Of Ceara | Fortaleza | Brazil |
| Gustavo | Coelho | Federal University Of Ceara | Fortaleza | Brazil |
| Ivens | Filizola Soares Machado | Federal University Of Ceara | Fortaleza | Brazil |
| Nubyhelia | Carvalho | Hospital Geral De Fortaleza | Fortaleza | Brazil |
| Lucio | Morais | University Federal Of The Goiás | Goiânia | Brazil |
| Aldo | Vieira Barros | Santa Casa De Misericórdia De Maceió | Maceió | Brazil |
| Gustavo | Gomes | Santa Casa De Misericórdia De Maceió | Maceió | Brazil |
| Igor | Lima Buarque | Santa Casa De Misericórdia De Maceió | Maceió | Brazil |
| Alessandro | Bersch Osvaldt | Hospital De Clínicas De Porto Alegre | Porto Alegre | Brazil |
| Matheus | Militz | Hospital De Clínicas De Porto Alegre | Porto Alegre | Brazil |
| Marcio | Boff | Hospital Mae De Deus | Porto Alegre | Brazil |
| Luciano | Marcelino | Hospital Moinhos De Vento | Porto Alegre | Brazil |
| Enilde | Guerra | Hospital Nossa Senhora Da Conceição | Porto Alegre | Brazil |
| Lucas | Torelly | Hospital Nossa Senhora Da Conceição | Porto Alegre | Brazil |
| Fábio Luiz | Waechter | Ufcspa / Santa Casa Porto Alegre | Porto Alegre | Brazil |
| Pablo | Rodrigues | Ufcspa / Santa Casa Porto Alegre | Porto Alegre | Brazil |
| Uirá | Fernandes Teixeira | Ufcspa / Santa Casa Porto Alegre | Porto Alegre | Brazil |
| Alessandro | Osvaldt | Universidade Federal Do Rio Grande Do Sul | Porto Alegre | Brazil |
| Luciano | Marcelino | Universidade Federal Do Rio Grande Do Sul | Porto Alegre | Brazil |
| Matheus | Militz | Universidade Federal Do Rio Grande Do Sul | Porto Alegre | Brazil |
| Eduardo | De Mello | Inca | Rio De Janeiro | Brazil |
| Rinaldo | Goncalves | Inca | Rio De Janeiro | Brazil |
| Silvio | Balzan | University Of Santa Cruz Do Sul | Santa Cruz Do Sul | Brazil |
| Eduardo | Jose B. Ramos | Dutra University Hospital, Maranhão Federal University | Sao Luis | Brazil |
| Jose Maria | Assunção Moraes-Junior | Dutra University Hospital, Maranhão Federal University | Sao Luis | Brazil |
| Orlando Jorge M | Torres | Dutra University Hospital, Maranhão Federal University | Sao Luis | Brazil |
| Diego | Vaz da Silva | Ac Camargo Cancer Center | São Paulo | Brazil |
| Felipe | Coimbra | Ac Camargo Cancer Center | São Paulo | Brazil |
| Felipe José | Fernández Coimbra | Ac Camargo Cancer Center | São Paulo | Brazil |
| Narimã | Marques | Ac Camargo Cancer Center | São Paulo | Brazil |
| Narimã | Marques | Ac Camargo Cancer Center | São Paulo | Brazil |
| Silvio | Melo Torres | Ac Camargo Cancer Center | São Paulo | Brazil |
| Adriano | Sampaio | Hospital Do Servidor Público Estadual De São Paulo | São Paulo | Brazil |
| Carlos | Augusto Canteras | Hospital Do Servidor Público Estadual De São Paulo | São Paulo | Brazil |
| Fabio | Ferreira | Santa Casa De São Paulo School of Medical Sciences | São Paulo | Brazil |
| Marcel Autran | Machado | Sirio Libanes Hospital | São Paulo | Brazil |
| Diego | Kleinubing | Santa Casa De Uruguaiana | Uruguaiana | Brazil |
| Livia | Lellis | Santa Casa De Uruguaiana | Uruguaiana | Brazil |
| Sarah L. | Brum | Santa Casa De Uruguaiana | Uruguaiana | Brazil |
| Muhammad | Gohar | Medical University Pleven | Pleven | Bulgaria |
| Boiko | Atanasov | Umhat Eurohospital | Plovdiv | Bulgaria |
| Mihail | Tanev Slavchev | Umhat Eurohospital | Plovdiv | Bulgaria |
| Mihail | Slavchev | Umhat Eurohospital | Plovdiv | Bulgaria |
| Nikolay | Belev | Umhat Eurohospital | Plovdiv | Bulgaria |
| Panche | Krastev | Umhat Eurohospital | Plovdiv | Bulgaria |
| Ivelin | Takorov | Military Medical Academy | Sofia | Bulgaria |
| Nikola | Vladov | Military Medical Academy | Sofia | Bulgaria |
| Radoslav | Kostadinov | Military Medical Academy | Sofia | Bulgaria |
| Tsonka | Lukanova | Military Medical Academy | Sofia | Bulgaria |
| Vassil | Mihaylov | Military Medical Academy | Sofia | Bulgaria |
| Plamen | Milchev Chernopolsky | Medical University | Varna | Bulgaria |
| Rossen | Madjov | Medical University | Varna | Bulgaria |
| Vasil | Markov Bozhkov | Medical University | Varna | Bulgaria |
| Vasil | Danielov Kostov | Military Medical Academy | Varna | Bulgaria |
| Daniel | Kostov | Military Medical Academy | Varna | Bulgaria |
| Evgeni | Nikolaev | Military Medical Academy | Varna | Bulgaria |
| Fabrice | Muhezagiro | University Of Burundi | Bujumbura | Burundi |
| Jérémie | Niyonkuru | University Of Burundi | Bujumbura | Burundi |
| Pacifique | Irakoze | University Of Burundi | Bujumbura | Burundi |
| Elijah | Dixon | University of Calgary | Calgary, Alberta | Canada |
| Elisabeth | Lo | McMaster University | Hamilton | Canada |
| Leyo | Ruo | McMaster University | Hamilton | Canada |
| Daniel | D'Souza | McMaster University | Hamilton | Canada |
| Pablo E. | Serrano | McMaster University | Hamilton | Canada |
| Anton | Skaro | London Health Sciences Centre - Western University | London - Ontario | Canada |
| Ephraim | Tang | London Health Sciences Centre - Western University | London - Ontario | Canada |
| Juan | Glinka | London Health Sciences Centre - Western University | London - Ontario | Canada |
| Janet | Martin | Western University | London - Ontario | Canada |
| George | Zogopoulos | Mcgill University | Montreal | Canada |
| Peter | Metrakos | Mcgill University | Montreal | Canada |
| Prosanto | Chaudhury | Mcgill University | Montreal | Canada |
| Rodrigo | Torres-Quevedo | Hospital Guillermo Grant Benavente | Concepción | Chile |
| Alejandro | Brañes | Hospital Dr Sotero Del Rio | Santiago | Chile |
| Alejandro | Brañes | Hospital Dr Sotero Del Rio | Santiago | Chile |
| Cristian | Diaz | Hospital Dr Sotero Del Rio | Santiago | Chile |
| Erwin | Buckel | Hospital Dr Sotero Del Rio | Santiago | Chile |
| Jean | Butte | Instituto Oncologico Falp | Santiago | Chile |
| Nicolas | Devaud | Instituto Oncologico Falp | Santiago | Chile |
| Luis | Paqui | Hospital Salvador | Santiago Chile | Chile |
| Kongyuan | Wei | Heidelberg University Hospital/ Chinese Pla General Hospital | Beijing | China |
| Huaizhi | Wang | University Of Chinese Academy Of Sciences | Chongqing | China |
| Lei | Cai | University Of Chinese Academy Of Sciences | Chongqing | China |
| Shixing | Guo | University Of Chinese Academy Of Sciences | Chongqing | China |
| Yiming | Chen | The First Affiliated Hospital Of Dali University | Dali | China |
| Maher | Hendi | Zhejiang University School Of Medicine Sir Ren Ren Shaw Hospital | Hangzhou | China |
| Tan To | Cheung | University of Hong Kong | Hong Kong | China |
| Carlos | Millan | Hospital San José | Bogota | Colombia |
| Pedro | Argüello | Clínica Valle del Lili | Cali | Colombia |
| Goran | Pavlek | University Hospital Centre Zagreb | Zagreb | Croatia |
| Hrvoje | Silovski | University Hospital Centre Zagreb | Zagreb | Croatia |
| Igor | Petrovic | University Hospital Centre Zagreb | Zagreb | Croatia |
| Ivan | Romic | University Hospital Centre Zagreb | Zagreb | Croatia |
| Jurica | Zedelj | University Hospital Centre Zagreb | Zagreb | Croatia |
| Fedor | Amic | University Hospital Dubrava | Zagreb | Croatia |
| Marijan | Kolovrat | University Hospital Dubrava | Zagreb | Croatia |
| Mislav | Rakic | University Hospital Dubrava | Zagreb | Croatia |
| Danko | Mikulic | University Hospital Merkur | Zagreb | Croatia |
| Ivan | Štironja | University Hospital Merkur | Zagreb | Croatia |
| Tomislav | Bubalo | University Hospital Merkur | Zagreb | Croatia |
| Nikolaos | Gouvas | University of Cyprus – Nicosia General Hospital | Nicosia | Cyprus |
| Panayiotis | Papatheodorou | University of Cyprus – Nicosia General Hospital | Nicosia | Cyprus |
| Thalis | Christophides | University of Cyprus – Nicosia General Hospital | Nicosia | Cyprus |
| Lukas | Burda | Comprehensive Cancer Centre And Agel Research And Training Institute | Nový Jičín | Czech Republic |
| Martin | Straka | Comprehensive Cancer Centre And Agel Research And Training Institute | Nový Jičín | Czech Republic |
| Dusan | Klos | University Hospital Olomouc | Olomouc | Czech Republic |
| Jana | Tesarikova | University Hospital Olomouc | Olomouc | Czech Republic |
| Martin | Loveček | University Hospital Olomouc | Olomouc | Czech Republic |
| Michal | Gregorik | University Hospital Olomouc | Olomouc | Czech Republic |
| Pavel | Skalicky | University Hospital Olomouc | Olomouc | Czech Republic |
| Christiana | Stögerová | University Hospital Pilsen | Pilsen | Czech Republic |
| Jakub | Fichtl | University Hospital Pilsen | Pilsen | Czech Republic |
| Skalický | Tomáš | University Hospital Pilsen | Pilsen | Czech Republic |
| Pavel | Zaruba | Military University Hospital Prague | Prague | Czech Republic |
| Andrej | Nikov | University Hospital Kralovske Vinohrady Prague | Prague | Czech Republic |
| Christoph | Tschuor | Rigshospitalet Copenhagen | Copenhagen | Denmark |
| Mohamed | Mohamed | Misr University For Science And Technology | 6Th October | Egypt |
| Bassant | Sayed | Misr University For Science And Technology | 6Th October City, Giza | Egypt |
| Ahmed | Shaheen | Alexanderia Faculty Of Medicine | Alexanderia | Egypt |
| Ahmed | Farid | Alexanderia University Main Hospital | Alexanderia | Egypt |
| Almoatazbellah | Attalla | Alexanderia University Main Hospital | Alexanderia | Egypt |
| Dalia | Fathallah Ibrahim | Alexanderia University Main Hospital | Alexanderia | Egypt |
| Dalia | Fathallah | Alexanderia University Main Hospital | Alexanderia | Egypt |
| Eman | Elmzaien | Alex Faculty Of Medicine | Alexandria | Egypt |
| Bothina | Magdy | Alex School Of Medicine | Alexandria | Egypt |
| Samer | Salah | Alexandria | Alexandria | Egypt |
| Ahmed | Saleh | Alexandria Faculty Of Medicine | Alexandria | Egypt |
| Ahmed | Abd Elglel Saker | Alexandria Faculty Of Medicine | Alexandria | Egypt |
| Ahmed | Swealem | Alexandria Faculty Of Medicine | Alexandria | Egypt |
| Esraa | Ibrahim Sallam | Alexandria Faculty Of Medicine | Alexandria | Egypt |
| Hebatullah | Rozza | Alexandria Faculty Of Medicine | Alexandria | Egypt |
| Mahmoud | Bassiony | Alexandria Faculty Of Medicine | Alexandria | Egypt |
| Manar | Elhassan | Alexandria Faculty Of Medicine | Alexandria | Egypt |
| Merna | Elmalah | Alexandria Faculty Of Medicine | Alexandria | Egypt |
| Mohamed | Belal | Alexandria Faculty Of Medicine | Alexandria | Egypt |
| Mohamed | El Gohary | Alexandria Faculty Of Medicine | Alexandria | Egypt |
| Mohamed Atef | Hassanin | Alexandria Faculty Of Medicine | Alexandria | Egypt |
| Nada | Elsayed | Alexandria Faculty Of Medicine | Alexandria | Egypt |
| Shiamaa | Aboelfath | Alexandria Faculty Of Medicine | Alexandria | Egypt |
| Islam | El-Sayes | Alexandria Main Hospital | Alexandria | Egypt |
| Mosaab | Tayiawi | Alexandria Main Hospital | Alexandria | Egypt |
| Abdulrahman | Altatari | Alexandria Main Hospital University | Alexandria | Egypt |
| Ahmad | Mohammad Altatari | Alexandria Main Hospital University | Alexandria | Egypt |
| Ahmed | Saleh | Alexandria Main University Hospital | Alexandria | Egypt |
| Mostafa Shehata | Qatora | Alexandria Main University Hospital | Alexandria | Egypt |
| Mohamed | Said | Alexandria School Of Medicine | Alexandria | Egypt |
| Amr | Najjar | Alexandria University | Alexandria | Egypt |
| Farouq | Alahmed | Alexandria University | Alexandria | Egypt |
| Fatimah | Mardhiyyah Binti Zamri | Alexandria University | Alexandria | Egypt |
| Hajer | Ealreibi | Alexandria University | Alexandria | Egypt |
| Hoor | Alahmed | Alexandria University | Alexandria | Egypt |
| Ismaeil | Alyasin | Alexandria University | Alexandria | Egypt |
| Karim | Abdelhalim | Alexandria University | Alexandria | Egypt |
| Maryam | Abd Alfatah | Alexandria University | Alexandria | Egypt |
| Mohamed | Abdallah Sharaan | Alexandria University | Alexandria | Egypt |
| Mohamed | Abd El Moneam | Alexandria University | Alexandria | Egypt |
| Mohamed | Abdelalemm | Alexandria University | Alexandria | Egypt |
| Mohamed | Mourad | Alexandria University | Alexandria | Egypt |
| Najiha | Binti Sohaimee | Alexandria University | Alexandria | Egypt |
| Nour Eldin | Abosamak | Alexandria University | Alexandria | Egypt |
| Nur | Mazlia Farzana Binti Suhaimi | Alexandria University | Alexandria | Egypt |
| Shaher | Shokralla | Alexandria University | Alexandria | Egypt |
| Yomna E. | Dean | Alexandria University | Alexandria | Egypt |
| Yousef | Tanas | Alexandria University | Alexandria | Egypt |
| Zuraiha | Waffa | Alexandria University | Alexandria | Egypt |
| Ahmed | Nafea | Alexandria University Faculty Of Medicine | Alexandria | Egypt |
| Dina | Ramadan | Alexandria University Hospital | Alexandria | Egypt |
| Abdelrahman | Abdelaal | Alexandria University Main Hospital | Alexandria | Egypt |
| Abdelrahman | Mahmoud | Alexandria University Main Hospital | Alexandria | Egypt |
| Ahmed | Mahmoud Nafea | Alexandria University Main Hospital | Alexandria | Egypt |
| Ahmed S. A. M. E. | Abuali | Alexandria University Main Hospital | Alexandria | Egypt |
| Islam | Korayem | Alexandria University Main Hospital | Alexandria | Egypt |
| Marina | Fahmy | Alexandria University Main Hospital | Alexandria | Egypt |
| Menna | Ibraheem | Alexandria University Main Hospital | Alexandria | Egypt |
| Mohammed | Hamouda | Alexandria University Main Hospital | Alexandria | Egypt |
| Rana | Helaly | Alexandria University Main Hospital | Alexandria | Egypt |
| Yazan | Fayez Khdour | Alexandria University Main Hospital | Alexandria | Egypt |
| Yazan | Khdour | Alexandria University Main Hospital | Alexandria | Egypt |
| Marina | Farag | Alexandria University Of Medicine | Alexandria | Egypt |
| Abdelrahman | Ibrahim | Alexandria University, Faculty Of Medicine | Alexandria | Egypt |
| Hajer | Ehab Elareibi | Alexandria University. | Alexandria | Egypt |
| Muneera | Alboridy | Alexandrian Faculty Of Medicine | Alexandria | Egypt |
| Ahmed | Mansour | Faculty Of Medicine | Alexandria | Egypt |
| Mohamed | Galal Ragab | Faculty Of Medicine | Alexandria | Egypt |
| Mohamed | Naguib | Faculty Of Medicine | Alexandria | Egypt |
| Shrouq | Allam | Faculty Of Medicine | Alexandria | Egypt |
| Hagar | Abo Elfarag | Faculty Of Medicine - Alexandria University | Alexandria | Egypt |
| Abdelrahman | Elsakka | Faculty Of Medicine Alexandria University | Alexandria | Egypt |
| Doaa | Mannaa | Faculty Of Medicine Alexandria University | Alexandria | Egypt |
| Mostafa | Elkeleny | Faculty Of Medicine Alexandria University | Alexandria | Egypt |
| Nurul | Ain Batrisyia Suhaimi | Faculty Of Medicine Alexandria University | Alexandria | Egypt |
| Sofia | Suhda Binti Mohd Uzir | Faculty Of Medicine Alexandria University | Alexandria | Egypt |
| Sara | Nasr | Faculty Of Medicine, Alexandria University | Alexandria | Egypt |
| Amro | El-Najjar | Gamal Abdel Nasser Hospital | Alexandria | Egypt |
| Mohamed | Dohien | Gamal Abdel Nasser Hospital | Alexandria | Egypt |
| Mohamed | Dohien | Gamal Abdel Nasser Hospital | Alexandria | Egypt |
| Nermin | Osman | Medical Research Institute | Alexandria | Egypt |
| Nuran | Gad | Mowasah University | Alexandria | Egypt |
| Mohamed | Hassanin | School Of Medicine, Alexandria University, Egypt | Alexandria | Egypt |
| Bashir A. | Fadel | Al-Rajhi Liver Hospital | Assiut | Egypt |
| Eman | Hassan Mohamed Hamdan | Al-Rajhi Liver Hospital | Assiut | Egypt |
| Fatma | Monib | Al-Rajhi Liver Hospital | Assiut | Egypt |
| Mahmoud | Saad | Assiut Faculty Of Medicine | Assiut | Egypt |
| Ahmed | Abbas | Assiut University Hospital | Assiut | Egypt |
| Ahmed | Mohammed Abu-Elfatth | Assiut University Hospital | Assiut | Egypt |
| Hossam Aldein | Abd Elazeem | Assiut University Hospital | Assiut | Egypt |
| Mohammed | Hisham Zayan Abdelhafez | Assiut University Hospital | Assiut | Egypt |
| Nehal | Omar | Assiut University Hospital | Assiut | Egypt |
| Ramy | Hassan | Assiut University Hospital | Assiut | Egypt |
| Ahmed | Mohamed | Assiut University Hospitals | Assiut | Egypt |
| Samir | Hosney Mahmoud | Assiut University Hospitals | Assiut | Egypt |
| Abobakr | Mahfoz Abobakr | Assiut Universty Hospital | Assiut | Egypt |
| Esraa | Essam Elsayed Mohamed | Assiut Universty Hospital | Assiut | Egypt |
| Randa | Ahmed | Assiut Universty Hospital | Assiut | Egypt |
| Hesham | Mahmoud Hamza | South Egypt Cancer Institute, Assiut, Egypt | Assiut | Egypt |
| Mahmoud | Mohammed | South Egypt Cancer Institute, Assiut, Egypt | Assiut | Egypt |
| Mohamed | Ali Marshod | South Egypt Cancer Institute, Assiut, Egypt | Assiut | Egypt |
| Ahmed | Mokhtar Mahmoud Hussein | Al-Rajhi Liver Hospital | Assuit | Egypt |
| Ahmed | Taha | Al-Rajhi Liver Hospital | Assuit | Egypt |
| Islam | Ibrahim | Al-Rajhi Liver Hospital | Assuit | Egypt |
| Mariam Albatoul | Nageh | Al-Rajhi Liver Hospital | Assuit | Egypt |
| Mohammed | Nageh Fouly | Al-Rajhi Liver Hospital | Assuit | Egypt |
| Ramy | Abdelrahim Hassan | Al-Rajhi Liver Hospital | Assuit | Egypt |
| Ahmed | Kamel Ali Mohamed | Al-Rajhi Liver Hospital | Assuit | Egypt |
| Mahmoud | Hasab Elnabi | Al-Rajhi Liver Hospital | Assuit | Egypt |
| Mohamed | Salah | Al-Rajhi Liver Hospital | Assuit | Egypt |
| Ahmed | Youssef Mohamed Ali | Assuit University Hospital | Assuit | Egypt |
| Esraa | Gamal Ahmed Sayed | Assuit University Hospital | Assuit | Egypt |
| Reem | Sayad | Assuit University Hospital | Assuit | Egypt |
| Mahmoud M. | Saad | Assuit Universty Hospital | Assuit | Egypt |
| Mohamed | Abdelkarem | Assuit Universty Hospital | Assuit | Egypt |
| Nehal | Gamal Omar | Assuit Universty Hospital | Assuit | Egypt |
| Alaa | Khalifa | Banha University Hospital | Banha | Egypt |
| Hazem | Faragalla | Ain Shams University | Benha | Egypt |
| Ahmed | Barakat | Benha University | Benha | Egypt |
| Ahmed | Tarek Mohamed Barakat | Benha University Hospital | Benha | Egypt |
| Ahmed | Elshafey | Benha University Hospital | Benha | Egypt |
| Mahmoud | Fares Eleisawy | Benha University Hospital | Benha | Egypt |
| Mahmoud | Eleisawy | Benha University Hospital | Benha | Egypt |
| Mohamed | Samir Mohamed Zahed | Benha University Hospital | Benha | Egypt |
| Mohamed | Zahed | Benha University Hospital | Benha | Egypt |
| Mohammed | Omer | Benha University Hospital | Benha | Egypt |
| Mohamed | Allam | Benha University Hospitals | Benha | Egypt |
| Yasmeen | Abuelnaga | Ain Shams Specialized Hospital | Cairo | Egypt |
| Abdurrahman | Abdelzaher | Ain Shams University | Cairo | Egypt |
| Ahmed | Alnimr | Ain Shams University | Cairo | Egypt |
| Hany | Dabbous | Ain Shams University | Cairo | Egypt |
| Hatem | Sayed | Ain Shams University | Cairo | Egypt |
| Ibrahim | Elgarhy | Ain Shams University | Cairo | Egypt |
| Mahmoud | Elmeteini | Ain Shams University | Cairo | Egypt |
| Mohamed | Bahaa | Ain Shams University | Cairo | Egypt |
| Mostafa | Farag | Ain Shams University | Cairo | Egypt |
| Mouhanad | Eid | Ain Shams University | Cairo | Egypt |
| Omar | Anas | Ain Shams University | Cairo | Egypt |
| Omar | Ismail | Ain Shams University | Cairo | Egypt |
| Omar | Nageeb | Ain Shams University | Cairo | Egypt |
| Reham | Lasheen | Ain Shams University | Cairo | Egypt |
| Samuel | Tanyous | Ain Shams University | Cairo | Egypt |
| Sherein | Diab | Ain Shams University | Cairo | Egypt |
| Youssef | Badran | Ain Shams University | Cairo | Egypt |
| Abdelrahman | Fahim | Ain Shams University Hospitals | Cairo | Egypt |
| Emad | Alazab | Ain Shams University Hospitals | Cairo | Egypt |
| Ibrahim | Mohamed Elgarhy | Ain Shams University Hospitals | Cairo | Egypt |
| Mahmoud | Abdeljalil | Ain Shams University Hospitals | Cairo | Egypt |
| Marya | Hanna | Ain Shams University Hospitals | Cairo | Egypt |
| Mira | Gobran | Ain Shams University Hospitals | Cairo | Egypt |
| Mira | Gobran | Ain Shams University Hospitals | Cairo | Egypt |
| Mohamed | Osama Mohammed Kamel Abdelmawla | Ain Shams University Hospitals | Cairo | Egypt |
| Mostafa | Nagy | Ain Shams University Hospitals | Cairo | Egypt |
| Omar | Emad Nageeb | Ain Shams University Hospitals | Cairo | Egypt |
| Salma | Ramadan | Ain Shams University Hospitals | Cairo | Egypt |
| Sherif | Abdelmawgoud | Ain Shams University Hospitals | Cairo | Egypt |
| Taha | Zidan | Ain Shams University Hospitals | Cairo | Egypt |
| Yasmeen | Abuelnaga | Ain Shams University Hospitals | Cairo | Egypt |
| Yasmeen | Tarkhan | Ain Shams University Hospitals | Cairo | Egypt |
| Ahmed | Saad | Ain Shams University Hospitals. | Cairo | Egypt |
| Ahmed K. | Awad | Ain Shams University Hospitals. | Cairo | Egypt |
| Merihan A | Elbadawy | Ain Shams University Hospitals. | Cairo | Egypt |
| Mohamed | Abdelmawla | Ain Shams University Hospitals. | Cairo | Egypt |
| Emad | Mansy | Al Azhar Medical School | Cairo | Egypt |
| Modather | Moharam | Al- Hussein University Hospital | Cairo | Egypt |
| Mohamed | Elabd | Al-Azhar University | Cairo | Egypt |
| Ahmed | Eldabour | Al-Hussein University Hospital | Cairo | Egypt |
| Lama | Elwakil | Cairo University | Cairo | Egypt |
| Marwan | Sayed Hassanien | Cairo University | Cairo | Egypt |
| Amr | Elnashar | Children Cancer Hospital | Cairo | Egypt |
| Hossam El-Dien | Saleh | Demerdash Hospital, Ainshams University | Cairo | Egypt |
| Marina | Michail | Dmerdash | Cairo | Egypt |
| Ahmed | Said | Ein Shams University | Cairo | Egypt |
| Mahmoud | El Garhy | Ein Shams University | Cairo | Egypt |
| Mohamed | Bahaa Eldin Ahmed | Ein Shams University | Cairo | Egypt |
| Omar | Anas | El Demerdash Hospital Asu | Cairo | Egypt |
| Omar | Ismail | Faculty Of Medicine Ain Shams University | Cairo | Egypt |
| Kirellos | Abboud | Faculty Of Medicine, Ain Shams University, Cairo, Egypt. | Cairo | Egypt |
| Ahmed | Nabil | Kasr Al Ainy Faculty Of Medicine, Cairo University | Cairo | Egypt |
| Mahmoud | Elfiky | Kasr Al Ainy Faculty Of Medicine, Cairo University | Cairo | Egypt |
| Abdelrahman | Murad | El Azhar University | Damanhour | Egypt |
| Ahmed | Azzam | Damietta Specialized Hospital | Damietta | Egypt |
| Mohammed A. | Azab | Damietta Specialized Hospital | Damietta | Egypt |
| Selmy | Awad | Mansoura University | Damietta | Egypt |
| Zeinab | Othman | Tanta University | Egypt | Egypt |
| Abdelrahman | Mohamed Fahim | Faculty Of Medicine-Ainshams University | El Quliobia | Egypt |
| Abdurrahman | Taha Abdelzaher | Faculty Of Medicine-Ainshams University | El Quliobia | Egypt |
| Taha | Zidan | Faculty Of Medicine-Ainshams University | El Quliobia | Egypt |
| Reham | Abdelrhman | Alexandria University | Elbehira | Egypt |
| Engy | Amgad Nasr Tolis | Alexandria Faculty Of Medicine | Elbuhera | Egypt |
| Mustafa | Salem | Ain Shams University Hospitals | Giza | Egypt |
| Hussein | Ebrahim | Azhar University | Giza | Egypt |
| Hussein A. | Abdelrazek | Azhar University | Giza | Egypt |
| Noha | Abdelmoneim | Cairo University (Kasr Al Ainy) | Giza | Egypt |
| Dinah | Salman | Kasr Al Ainy Medical School | Giza | Egypt |
| Hussam | Saa'd | Kasr Al Ainy Medical School | Giza | Egypt |
| Dina | Ali | Kasr Alainy Medical School | Giza | Egypt |
| Ahmed | Farouk | Gastrointestinal Surgery Center, Mansoura University | Mansoura | Egypt |
| Ahmed | Rafaat Mandor | Gastrointestinal Surgery Center, Mansoura University | Mansoura | Egypt |
| Ahmed | Monier | Gastrointestinal Surgery Center, Mansoura University | Mansoura | Egypt |
| Ahmed | Shehta | Gastrointestinal Surgery Center, Mansoura University | Mansoura | Egypt |
| Amr | Kassem | Gastrointestinal Surgery Center, Mansoura University | Mansoura | Egypt |
| Amr | Sanad | Gastrointestinal Surgery Center, Mansoura University | Mansoura | Egypt |
| Reem | Elsaadany | Mansoura Manchester Program For Medical Education | Mansoura | Egypt |
| Mohamed M. | Shaat | Mansoura University | Mansoura | Egypt |
| Rami | Elmorsi | Mansoura University | Mansoura | Egypt |
| Selmy | Awad | Mansoura University | Mansoura | Egypt |
| Soliman | Ghedan | Mansoura University | Mansoura | Egypt |
| Ahmed | Menessy | Mansoura University Hospitals | Mansoura | Egypt |
| Dina | Elnabawy | Mansoura University Hospitals | Mansoura | Egypt |
| Khaled | Abdou | Mansoura University Hospitals | Mansoura | Egypt |
| Mohamed | Abdelmaksoud | Mansoura University Hospitals | Mansoura | Egypt |
| Mohamed | Hassan | Mansoura University Hospitals | Mansoura | Egypt |
| Omnia | Elweza | Mansoura University Hospitals | Mansoura | Egypt |
| Rahma | Elboraei | Mansoura University Hospitals | Mansoura | Egypt |
| Ahmed | Abdallah | Oncology Center Mansoura University | Mansoura | Egypt |
| Islam H. | Metwally | Oncology Center Mansoura University | Mansoura | Egypt |
| Mohamed | Elhamamsy | Oncology Center Mansoura University | Mansoura | Egypt |
| Ahmed | M. Fareed | Oncology Centre Mansoura University | Mansoura | Egypt |
| Mohammad | Zuhdy | Oncology Centre Mansoura University | Mansoura | Egypt |
| Saleh | S. Elbalka | Oncology Centre Mansoura University | Mansoura | Egypt |
| Marwa | Nasrelden Alansary | Faculty Of Medicine, South Valley University | Qena | Egypt |
| Mohammed | Omar | Faculty Of Medicine, South Valley University | Qena | Egypt |
| Ahmed Abdelfattah | Elgharably | Faculty Of Medicine, South Valley University, Qena, Egypt | Qena | Egypt |
| Eman | Hager | National Liver Institute, Menoufia Univeraity | Shebeen El-Kom | Egypt |
| Ammar | El Gady | National Liver Institute, Menoufia Univeraity | Shebin | Egypt |
| Doaa | Sabry Alsharif | National Liver Institute, Menoufia Univeraity | Shebin | Egypt |
| Ammar | Magdy Shaaban | National Liver Institute, Menoufia Univeraity | Shebin Alkom | Egypt |
| Doaa | Alsharif | National Liver Institute, Menoufia Univeraity | Shebin Alkom | Egypt |
| Doaa | Samaan | National Liver Institute, Menoufia Univeraity | Shebin Alkom | Egypt |
| Samy Sameh Samy | Samaan | National Liver Institute, Menoufia Univeraity | Shebin Alkom | Egypt |
| Ahmed | Oteem | National Liver Institute, Menoufia Univeraity | Shebin El Koom | Egypt |
| Ammar | Magdy Shaaban | National Liver Institute, Menoufia Univeraity | Shebin El Koum | Egypt |
| Doaa | Sabry Alsharif | National Liver Institute, Menoufia Univeraity | Shebin El Koum | Egypt |
| Samy | Samaan | National Liver Institute, Menoufia Univeraity | Shebin El Koum | Egypt |
| Ahmed | Zayed | National Liver Institute, Menoufia Univeraity | Shebin El. Kom | Egypt |
| Ahmed | Allam | National Liver Institute, Menoufia Univeraity | Shebin El. Kom | Egypt |
| Ammar | El Gady | National Liver Institute, Menoufia Univeraity | Shebin El. Kom | Egypt |
| Doaa | Sabry Alsharif | National Liver Institute, Menoufia Univeraity | Shebin El. Kom | Egypt |
| Karim | Badr | National Liver Institute, Menoufia Univeraity | Shebin El. Kom | Egypt |
| Salma | Elnoamany | National Liver Institute, Menoufia Univeraity | Shebin El. Kom | Egypt |
| Sameh | Samy Samaan | National Liver Institute, Menoufia Univeraity | Shebin El. Kom | Egypt |
| Mohamed | Ellibady | Sohag University | Sohag | Egypt |
| Emad Ali | Ahmed | Sohag University | Sohag | Egypt |
| Ahmed | Elbassyiouny | Faculty Of Medicine Must University | Tanta | Egypt |
| Ahmed | Boalot | Tanta University | Tanta | Egypt |
| Helmy | Badr | Tanta University | Tanta | Egypt |
| Mohamed | Gamal | Tanta University | Tanta | Egypt |
| Mohamed | Abuelazm | Tanta University | Tanta | Egypt |
| Zeinab | Othman | Tanta University | Tanta | Egypt |
| Abdullah | Eldaly | Tanta University Hispital | Tanta | Egypt |
| Abdullah | Sami Eldaly | Tanta University Hospital | Tanta | Egypt |
| Mostafa | Essa | Tanta University Hospital | Tanta | Egypt |
| Fatma | Abdelrahman | Zagazig Uni | Zagazig | Egypt |
| Abdelrahman | Sarhan | Zagazig University | Zagazig | Egypt |
| Feras | Alsabbagh | Zagazig University | Zagazig | Egypt |
| Mohamed | Abd Allah | Zagazig University | Zagazig | Egypt |
| Abdulrahman | Bayomi | Zagazig University Hospital | Zagazig | Egypt |
| Moaz | Salama | Zagazig University Hospitals | Zagazig | Egypt |
| margus | kivisild | University Clinic Of Tartu | Tartu | Estonia |
| Olav | Tammik | University Clinic Of Tartu | Tartu | Estonia |
| Taavi | Podramagi | University Clinic Of Tartu | Tartu | Estonia |
| Heikki | Huhta | Oulu University Hospital | Oulu | Finland |
| Joonas H. | Kauppila | Oulu University Hospital | Oulu | Finland |
| Minna | Nortunen | Oulu University Hospital | Oulu | Finland |
| Lionel | Jouffret | Hôpital Henri Duffaut, Avignon | Avignon | France |
| Daniele | Sommacale | Hôpital Henri-Mondor, Assistance Publique-Hôpitaux de Paris, Université de Paris-Est | Créteil | France |
| Raffaele | Brustia | Hôpital Henri-Mondor, Assistance Publique-Hôpitaux de Paris, Université de Paris-Est | Créteil | France |
| Rim | Cherif | Hôpital Henri-Mondor, Assistance Publique-Hôpitaux de Paris, Université de Paris-Est | Créteil | France |
| Katia | Lecolle | Chu De Lille | Lille | France |
| Mehdi | El Amrani | Chu De Lille | Lille | France |
| Cesar | Beugniez | Hôpital Huriez, Chu Lille | Lille | France |
| Stéphanie | Truant | Hôpital Huriez, Chu Lille | Lille | France |
| Guillaume | Piessen | Lille University Hospital | Lille | France |
| Sebastien | Degisors | Lille University Hospital | Lille | France |
| Aurélien | Dupré | Centre Leon Berard | Lyon | France |
| Julie | Perinel | Edouard Herriot Hospital, Hcl, | Lyon | France |
| Mustapha | Adham | Edouard Herriot Hospital, Hcl, | Lyon | France |
| Olivia | Sgarbura | Institut du Cancer Montpellier | Montpellier | France |
| Francois-Regis | Souche | Chu Montpellier | Montpellier | France |
| Antonio | Iannelli | CHU de Nice, Hôpital de l' Archet | Nice | France |
| Jean | Gugenheim | CHU de Nice, Hôpital de l' Archet | Nice | France |
| Natalia | Savvala | CHU de Nice, Hôpital de l' Archet | Nice | France |
| Olivier | Scatton | Aphp | Nimes | France |
| Renato | Lupinacci | Ambroise Pare Hospital, Paris-Saclay University | Paris | France |
| Emilia | Ragot | Georges Pompidou European Hospital | Paris | France |
| Gilles | Manceau | Georges Pompidou European Hospital | Paris | France |
| Mehdi | Karoui | Georges Pompidou European Hospital | Paris | France |
| Nicolas | Goasguen | Hopital Diaconesses/Croix Saint Simon | Paris | France |
| Morgan | Anyla | Hôpital Pitié-Slapétrière | Paris | France |
| Sebastien | Gaujoux | Hôpital Pitié-Slapétrière, Sorbonne University | Paris | France |
| Rami | Rhaiem | Robert Debré University Hospital | Reims | France |
| Tullio | Piardi | University of Reims Champagne Ardenne | Reims | France |
| Fabien | Robin | University Hospital Rennes, France | Rennes | France |
| Laurent | Sulpice | University Hospital Rennes, France | Rennes | France |
| Edouard | Roussel | Rouen University Hospital | Rouen | France |
| Eloise | Papet | Rouen University Hospital | Rouen | France |
| Lilian | Schwarz | Rouen University Hospital | Rouen | France |
| Emanuele | Felli | Nouvel Hôpital Civil | Strasbourg | France |
| Fabio | Giannone | Nouvel Hôpital Civil | Strasbourg | France |
| Patrick | Pessaux | Nouvel Hôpital Civil | Strasbourg | France |
| Irakli | Pipia | Tbilisi State Medical University | Tbilisi | Georgia |
| Kakhi | Khutsishvili. | Tbilisi State Medical University | Tbilisi | Georgia |
| Zaza | Demetrashvili | Tbilisi State Medical University | Tbilisi | Georgia |
| Carsten | Krones | Marienhospital Aachen | Aachen | Germany |
| Hans-Peter | Wüllenweber | Marienhospital Aachen | Aachen | Germany |
| Isabel | Bartella | Marienhospital Aachen | Aachen | Germany |
| Carsten | Kamphues | Charité - Universitätsmedizin Berlin | Berlin | Germany |
| Florian | Loch | Charité - Universitätsmedizin Berlin | Berlin | Germany |
| Ioannis | Pozios | Charité - Universitätsmedizin Berlin | Berlin | Germany |
| Orlin | Belyaev | St. Josef Hospital, Ruhr University Bochum | Bochum | Germany |
| Prem Vignesh | Mohan | St. Josef Hospital, Ruhr University Bochum | Bochum | Germany |
| Waldemar | Uhl | St. Josef Hospital, Ruhr University Bochum | Bochum | Germany |
| Dirk | Bulian | Cologne-Merheim Medical Center, Witten/Herdecke University | Cologne | Germany |
| Niklas | Juengling | Cologne-Merheim Medical Center, Witten/Herdecke University | Cologne | Germany |
| Panagiotis | Thomaidis | Cologne-Merheim Medical Center, Witten/Herdecke University | Cologne | Germany |
| Sandra | Korn | University Hospital Carl Gustav Carus | Dresden | Germany |
| Thilo | Welsch | University Hospital Carl Gustav Carus | Dresden | Germany |
| Ulrich | Bork | University Hospital Carl Gustav Carus | Dresden | Germany |
| Christian | Praetorius | University Hospital Carl Gustav Carus Dresden | Dresden | Germany |
| Jürgen | Weitz | University Hospital Carl Gustav Carus Dresden | Dresden | Germany |
| Marius | Distler | University Hospital Carl Gustav Carus Dresden | Dresden | Germany |
| Christian | Krautz | University Hospital Erlangen | Erlangen | Germany |
| Maximilian | Brunner | University Hospital Erlangen | Erlangen | Germany |
| Robert | Grützmann | University Hospital Erlangen | Erlangen | Germany |
| Elena | Mazzella | University Hospital Frankfurt am Main | Frankfurt Am Main | Germany |
| Andreas | Hecker | University Hospital Of Giessen | Giessen | Germany |
| Martin | Reichert | University Hospital Of Giessen | Giessen | Germany |
| Azadeh | Azizian | University Medical Center Göttingen | Göttingen | Germany |
| Jochen | Gaedcke | University Medical Center Göttingen | Göttingen | Germany |
| Michael | Ghadimi | University Medical Center Göttingen | Göttingen | Germany |
| Ali | Aghdassi | University Medicine Greifswald | Greifswald | Germany |
| Jessica | Döbereiner | University Hospital Halle (Saale) | Halle (Saale) | Germany |
| Johannes | Klose | University Hospital Halle (Saale) | Halle (Saale) | Germany |
| Jörg | Kleeff | University Hospital Halle (Saale) | Halle (Saale) | Germany |
| Ulrich | Ronellenfitsch | University Hospital Halle (Saale) | Halle (Saale) | Germany |
| Karl J. | Oldhafer | Asklepios Hospital Barmbek | Hamburg | Germany |
| Ki | Wagner | Asklepios Hospital Barmbek | Hamburg | Germany |
| Tim | Reese | Asklepios Hospital Barmbek | Hamburg | Germany |
| Asmus | Heumann | University Hospital Hamburg-Eppendorf | Hamburg | Germany |
| Faik G. | Uzunoglu | University Hospital Hamburg-Eppendorf | Hamburg | Germany |
| Jakob | Izbicki | University Hospital Hamburg-Eppendorf | Hamburg | Germany |
| Mara | Goetz | University Hospital Hamburg-Eppendorf | Hamburg | Germany |
| Pasquale | Scognamiglio | University Hospital Hamburg-Eppendorf | Hamburg | Germany |
| Kim | Honselmann | University Hospital Schleswig Holstein Campus Lübeck | Lübeck | Germany |
| Tobias | Keck | University Hospital Schleswig Holstein Campus Lübeck | Lübeck | Germany |
| Ulrich | Wellner | University Hospital Schleswig Holstein Campus Lübeck | Lübeck | Germany |
| Benjamin | Struecker | Universitätsklinikum Münster (Ukm) | Münster | Germany |
| Christina | Hackl | University Medical Center Regensburg | Regensburg | Germany |
| Frank | W. Brennfleck | University Medical Center Regensburg | Regensburg | Germany |
| Stefan | Brunner | University Medical Center Regensburg | Regensburg | Germany |
| Dimitrios | Kardassis | Klinikum Saarbrücken | Saarbrücken | Germany |
| Frank | Schütze | Klinikum Saarbrücken | Saarbrücken | Germany |
| Gregor A, | Stavrou | Klinikum Saarbrücken | Saarbrücken | Germany |
| Omid | Ghamarnejad | Klinikum Saarbrücken | Saarbrücken | Germany |
| Ralf | Metzger | Klinikum Saarbrücken | Saarbrücken | Germany |
| Alfred | Koenigsrainer | University Hospital Tuebingen | Tuebingen | Germany |
| Silvio | Nadalin | University Hospital Tuebingen | Tuebingen | Germany |
| Christoph | Anthoni | St. Josefs-Hospital Wiesbaden | Wiesbaden | Germany |
| Georgios | Makridis | St. Josefs-Hospital Wiesbaden | Wiesbaden | Germany |
| Stefan A. | Farkas | St. Josefs-Hospital Wiesbaden | Wiesbaden | Germany |
| Stefan | Löb | University Hospital Würzburg | Würzburg | Germany |
| Efstathios | Nikou | 401 General Military Hospital Of Athens | Athens | Greece |
| Nikolaos | Tsoukalas | 401 General Military Hospital Of Athens | Athens | Greece |
| Eugenios | Bairamidis | 401 General Military Hospital Of Athens | Athens | Greece |
| Aliki | Vaia | Agios Savvas Anticancer Hospital Of Athens | Athens | Greece |
| Antonia | Prountzopoulou | Agios Savvas Anticancer Hospital Of Athens | Athens | Greece |
| Evangelos | Fradelos | Agios Savvas Anticancer Hospital Of Athens | Athens | Greece |
| Aristotelis | Kechagias | Athens Bioclinic Hospital | Athens | Greece |
| Dionysia | Kelgiorgi | Athens Bioclinic Hospital | Athens | Greece |
| Konstantinos | Avgerinos | Athens Bioclinic Hospital | Athens | Greece |
| Argyrios | Ioannidis | Athens Medical Center | Athens | Greece |
| Konstantinos M. | Konstantinidis | Athens Medical Center | Athens | Greece |
| Michael K. | Konstantinidis | Athens Medical Center | Athens | Greece |
| Dimitrios | Papakonstantinou | Attikon University Hospital | Athens | Greece |
| Ilectra | Papiri | Attikon University Hospital | Athens | Greece |
| Nikolaos | Michalopoulos | Attikon University Hospital | Athens | Greece |
| Zoe | Petropoulou | Attikon University Hospital | Athens | Greece |
| Spyridon | Christodoulou | Attikon University Hospital, University of Athens | Athens | Greece |
| Ioannis | Margaris | Attikon University Hospital, University Of Athens | Athens | Greece |
| Iosif | Chatzialis | Attikon University Hospital, University Of Athens | Athens | Greece |
| Jonida | Selmani | Attikon University Hospital, University Of Athens | Athens | Greece |
| Maria | Papadoliopoulou | Attikon University Hospital, University Of Athens | Athens | Greece |
| Nikolaos | Arkadopoulos | Attikon University Hospital, University Of Athens | Athens | Greece |
| Panagiotis | Kokoropoulos | Attikon University Hospital, University Of Athens | Athens | Greece |
| Pantelis | Vassiliu | Attikon University Hospital, University Of Athens | Athens | Greece |
| Stavros | Parasyris | Attikon University Hospital, University Of Athens | Athens | Greece |
| Theodoros | Sidiropoulos | Attikon University Hospital, University Of Athens | Athens | Greece |
| Paraskevas | Stamopoulos | Eugenideio Hospital | Athens | Greece |
| Dimitrios | Stergiou | Evangelismos General Hospital | Athens | Greece |
| Maria | Sotiropoulou | Evangelismos General Hospital | Athens | Greece |
| Michalis | Vaslamatzis | Evangelismos General Hospital | Athens | Greece |
| Nikolaos | Roukounakis | Evangelismos General Hospital | Athens | Greece |
| Stylianos A. | Kapiris | Evangelismos General Hospital | Athens | Greece |
| Stylianos A. | Kapiris | Evangelismos General Hospital | Athens | Greece |
| Vasileios | Vougas | Evangelismos General Hospital | Athens | Greece |
| Nikolaos | Roukounakis | Evangelismos Hpt | Athens | Greece |
| Dimitrios | Dimitroulis | General Hospital Laiko | Athens | Greece |
| Dimitrios | Mantas | General Hospital Laiko | Athens | Greece |
| Eugenia | Kotsifa | General Hospital Laiko | Athens | Greece |
| Eygenia | Kotsifa | General Hospital Laiko | Athens | Greece |
| Nefeli | Tomara | General Hospital Laiko | Athens | Greece |
| Nefeli Kaiti | Tomara | General Hospital Laiko | Athens | Greece |
| Nikolaos | Machairas | General Hospital Laiko | Athens | Greece |
| Panagiotis | Dorovinis | General Hospital Laiko | Athens | Greece |
| Stylianos | Kykalos | General Hospital Laiko | Athens | Greece |
| Theodoros | Tsirlis | General Oncology Hospital Of Athens "St Savvas" | Athens | Greece |
| Andreas | Larentzakis | Hippocratio General Hospital Of Athens | Athens | Greece |
| Gavriella Zoi | Vrakopoulou | Hippocratio General Hospital Of Athens | Athens | Greece |
| George | Tzimas | Hygeia Hospital | Athens | Greece |
| Spyridon | Pagkratis | Hygeia Hospital | Athens | Greece |
| Ioannis | Triantafyllidis | Konstantopoulio-Patision General Hospital Of Nea Ionia | Athens | Greece |
| Alexandros | Papalampros | National And Kapodistrian University Of Athens | Athens | Greece |
| Andreas | Polydorou | National And Kapodistrian University Of Athens | Athens | Greece |
| Athanasios | Syllaios | National And Kapodistrian University Of Athens | Athens | Greece |
| Christina | Kontopoulou | National And Kapodistrian University Of Athens | Athens | Greece |
| Dimitrios | Politis | National And Kapodistrian University Of Athens | Athens | Greece |
| Dimitrios | Vouros | National And Kapodistrian University Of Athens | Athens | Greece |
| Dimitrios | Schizas | National And Kapodistrian University Of Athens | Athens | Greece |
| Eleandros | Kyros | National And Kapodistrian University Of Athens | Athens | Greece |
| Evangelos | Felekouras | National And Kapodistrian University Of Athens | Athens | Greece |
| Ioannis | Karavokyros | National And Kapodistrian University Of Athens | Athens | Greece |
| John | Griniatsos | National And Kapodistrian University Of Athens | Athens | Greece |
| Konstantinos | Bramis | National And Kapodistrian University Of Athens | Athens | Greece |
| Konstantinos | Toutouzas | National And Kapodistrian University Of Athens | Athens | Greece |
| Lysandros | Karydakis | National And Kapodistrian University Of Athens | Athens | Greece |
| Manousos | Konstadoulakis | National And Kapodistrian University Of Athens | Athens | Greece |
| Nikolaos | Memos | National And Kapodistrian University Of Athens | Athens | Greece |
| Prodromos | Kanavidis | National And Kapodistrian University Of Athens | Athens | Greece |
| Dimitrios | Massaras | National and Kapodistrian University of Athens School of Medicine | Athens | Greece |
| Georgios | Fragulidis | National and Kapodistrian University of Athens School of Medicine | Athens | Greece |
| Maximos | Frountzas | National and Kapodistrian University of Athens School of Medicine | Athens | Greece |
| Kleoniki | Kordeni | National And Kapodistrian University Of Athens School Of Medicine | Athens | Greece |
| Antonios | Vezakis | National And Kapodistrian University Of Athens, Aretaieion Hospital | Athens | Greece |
| Konstantinos | Iliakopoulos | National And Kapodistrian University Of Athens, Aretaieion Hospital | Athens | Greece |
| Leonidas | Chardalias | National And Kapodistrian University Of Athens, Aretaieion Hospital | Athens | Greece |
| Ioannis | Kyriazanos | Naval And Veterans Hospital Of Athens | Athens | Greece |
| Ioannis | Kyriazanos | Naval And Veterans Hospital Of Athens | Athens | Greece |
| Meletios | Marougkas | Naval And Veterans Hospital Of Athens | Athens | Greece |
| Nikolaos | Stamos | Naval And Veterans Hospital Of Athens | Athens | Greece |
| Triantafyllos | Giannakopoulos | Naval And Veterans Hospital Of Athens | Athens | Greece |
| Vasileios | Kalles | Naval And Veterans Hospital Of Athens | Athens | Greece |
| Dimitrios | Balalis | Saint Savvas Cancer Hospital | Athens | Greece |
| Dimitrios | Manatakis | Saint Savvas Cancer Hospital | Athens | Greece |
| Dimitrios | Korkolis | Saint Savvas Cancer Hospital | Athens | Greece |
| Maria | Bourazani | Saint Savvas Cancer Hospital | Athens | Greece |
| Spiros | Delis | St Olga Hospital | Athens | Greece |
| Dimitrios | Cyrochristos | Ioannina University Hospital And School Of Medicine | Ioannina | Greece |
| Evangelos | Baltagiannis | Ioannina University Hospital And School Of Medicine | Ioannina | Greece |
| Georgios | Glantzounis | Ioannina University Hospital And School Of Medicine | Ioannina | Greece |
| Stylianos | Stylianidis | Hellenic Nhs | Kavala | Greece |
| Alexandros | Diamantis | University Hospital Of Larisa | Larisa | Greece |
| Alexandros | Valaroutsos | University Hospital Of Larisa | Larisa | Greece |
| Dimitrios | Magouliotis | University Hospital Of Larissa | Larissa | Greece |
| Dimitrios | Zacharoulis | University Hospital Of Larissa | Larissa | Greece |
| Grigorios | Christodoulidis | University Hospital Of Larissa | Larissa | Greece |
| Konstantinos | Tepetes | University Hospital Of Larissa | Larissa | Greece |
| Konstantinos | Perivoliotis | University Hospital Of Larissa | Larissa | Greece |
| Maria | Fergadi | University Of Thessaly | Larissa | Greece |
| Gregory | Tsiotos | Mitera-Hygeia Hospitals | Pallini | Greece |
| Francesk | Mulita | University Hospital Of Patras | Patra | Greece |
| Ioannis | Maroulis | University Hospital Of Patras | Patra | Greece |
| Michail | Vailas | University Hospital Of Patras | Patra | Greece |
| Apollon | Zygomalas | Olympion General Clinic Of Patras | Patras | Greece |
| Dionysios | Karavias | Olympion General Clinic Of Patras | Patras | Greece |
| Elissaios | Kontis | Metaxa Cancer Hospital | Piraeus | Greece |
| Ioannis | Katsaros | Metaxa Cancer Hospital | Piraeus | Greece |
| Nikolaos | Kopanakis | Metaxa Cancer Hospital | Piraeus | Greece |
| Andreas | Tooulias | Aristotle University Of Thessaloniki | Thessaloniki | Greece |
| Chrysanthos | Christou | Aristotle University Of Thessaloniki | Thessaloniki | Greece |
| Dimitrios | Raptis | Aristotle University Of Thessaloniki | Thessaloniki | Greece |
| Georgios | Katsanos | Aristotle University Of Thessaloniki | Thessaloniki | Greece |
| Nikolaos | Beradze | Aristotle University Of Thessaloniki | Thessaloniki | Greece |
| Vasileios | Papaziogas | Aristotle University Of Thessaloniki | Thessaloniki | Greece |
| Vasileios N. | Papadopoulos | Aristotle University Of Thessaloniki | Thessaloniki | Greece |
| Dimitris | Giakoustidis | Aristotle University Of Thessaloniki And Interbalkan Medical Center | Thessaloniki | Greece |
| Anastasios | Katsourakis | General Hospital of Thessaloniki "O Agios Dimitrios". | Thessaloniki | Greece |
| Evripidis | Efthymiou | General Hospital of Thessaloniki "O Agios Dimitrios". | Thessaloniki | Greece |
| Iosif | Chatzis | General Hospital of Thessaloniki "O Agios Dimitrios". | Thessaloniki | Greece |
| Achilleas | Νtinas | Interbalkan Medical Center | Thessaloniki | Greece |
| Efthimios | Hatzitheoklitos | Interbalkan Medical Center | Thessaloniki | Greece |
| Konstantinos | Tsalis | Interbalkan Medical Center | Thessaloniki | Greece |
| Pavlos | Koustas | Interbalkan Medical Center | Thessaloniki | Greece |
| Kambaroudis | Apostolos | Ippokratio General Hospital Of Thessaloniki | Thessaloniki | Greece |
| Panagiotis | Petras | Ippokratio General Hospital Of Thessaloniki | Thessaloniki | Greece |
| Savvas | Tsaramanidis | Ippokratio General Hospital Of Thessaloniki | Thessaloniki | Greece |
| Charalampos | Iakovidis | Saint Luke’S Hospital | Thessaloniki | Greece |
| Emmanouil | Zacharakis | St Lukes Hospital | Thessaloniki | Greece |
| Tamás | Marjai | Semmeweis Unversity | Budapest | Hungary |
| Attila | Bursics | Uzsoki Hospital | Budapest | Hungary |
| Kristóf | Dede | Uzsoki Hospital | Budapest | Hungary |
| Tamás | Tölgyes | Uzsoki Hospital | Budapest | Hungary |
| Andras | Vereczkei | Clinical Center, Medical Faculty, University of Pécs | Pécs | Hungary |
| Dezső | Kelemen | Clinical Center, Medical Faculty, University of Pécs | Pécs | Hungary |
| Papp | Robert | Clinical Center, Medical Faculty, University of Pécs | Pécs | Hungary |
| Bhavin | Vasavada | Shalby Hospitals | Ahmedabad | India |
| Dhaivat | Vaishnav | Zydus Hospital | Ahmedabad | India |
| Praful | Pawar | Mgm Medical College And Hospital | Aurangabad | India |
| Pravin | Suryawanshi | Mgm Medical College And Hospital | Aurangabad | India |
| R. M. | Shinde | Mgm Medical College And Hospital | Aurangabad | India |
| Charoo | Piplani | Clara Swain Mission Hospital | Bareilly | India |
| Ayaskanta | Singh | Ims And Sum Hospital | Bhubaneswar | India |
| Saroj | Kanta Sahu | Ims And Sum Hospital | Bhubaneswar | India |
| Satyaprakash Ray | Choudhary | Ims And Sum Hospital | Bhubaneswar | India |
| Rajesh | Gupta | Pgimer | Chandigarh | India |
| Anand | Ramamurthy | Hindu Mission Hospital | Chennai | India |
| Babu | E. | Sri Ramachandra Institute Of Higher Education And Research | Chennai | India |
| Sreenivasan | Karuparthi | Sri Ramachandra Institute Of Higher Education And Research | Chennai | India |
| Suresh | Kumar | Sri Ramachandra Institute Of Higher Education And Research | Chennai | India |
| Govind | Purushothaman | Stanley Medical College | Chennai | India |
| Jeswanth | Sathyanesan | Stanley Medical College | Chennai | India |
| Venkatesh | N. R. | Stanley Medical College | Chennai | India |
| Solomon | John | Emc Hospital, Apollo Adlux Hospital & Welcare Hospital | Cochin | India |
| Arvind K. | Singh | Synergy Institute of Medical Sciences | Dehradun | India |
| Rahul | Gupta | Synergy Institute of Medical Sciences | Dehradun | India |
| Sudhir K. | Singh | Synergy Institute of Medical Sciences | Dehradun | India |
| Dharmender | Sharma | W Pratiksha Hospital, Gurgaon | Gurugram | India |
| Kaushal | Yadav | W Pratiksha Hospital, Gurgaon | Gurugram | India |
| Nitin | Leekha | W Pratiksha Hospital, Gurgaon | Gurugram | India |
| Rashmitha | Pippari | Osmania Medical College/Osmania General Hospital | Hyderabad | India |
| Manoj | Pandey | Institute of Medical Sciences, Banaras Hindu University, Varanasi | India | India |
| Neville | Joseph Francis | Institute of Medical Sciences, Banaras Hindu University, Varanasi | India | India |
| Tarun | Kumar | Institute of Medical Sciences, Banaras Hindu University, Varanasi | India | India |
| Sundeep | Jain | Ck Birla Hospitals/ Rbh Jaipur, India | Jaipur | India |
| Dharma | Ram Poonia | All India Institute of Medical Sciences Jodhpur | Jodhpur | India |
| Jeewan Ram | Vishnoi | All India Institute of Medical Sciences Jodhpur | Jodhpur | India |
| Nivedita | Sharma | All India Institute of Medical Sciences Jodhpur | Jodhpur | India |
| Puneet | Pareek | All India Institute of Medical Sciences Jodhpur | Jodhpur | India |
| Rajendar | Byshetty | All India Institute of Medical Sciences Jodhpur | Jodhpur | India |
| Sanjeev | Misra | All India Institute of Medical Sciences Jodhpur | Jodhpur | India |
| Vaibhav | Varshney | All India Institute of Medical Sciences Jodhpur | Jodhpur | India |
| Ramdip | Ray | Apollo Hospital, Kolkata | Kolkata | India |
| Sumit | Gulati | Apollo Hospital, Kolkata | Kolkata | India |
| Supriyo | Ghatak | Apollo Hospital, Kolkata | Kolkata | India |
| Kshaunish | Das | Institute Of Postgraduate Medical Education & Research | Kolkata | India |
| Sujan | Khamrui | Institute Of Postgraduate Medical Education & Research | Kolkata | India |
| Sukanta | Ray | Institute Of Postgraduate Medical Education & Research | Kolkata | India |
| George | Mathew Sebastian | Caritas Hospital | Kottayam | India |
| Jithin | Thulsi Chand | Caritas Hospital | Kottayam | India |
| Murali | Appukuttan | Caritas Hospital | Kottayam | India |
| Arun | Chaturvedi | King George'S Medical University | Lucknow | India |
| Naseem | Akhtar | King George'S Medical University | Lucknow | India |
| Puneet | Prakash | King George'S Medical University | Lucknow | India |
| Sameer | Gupta | King George'S Medical University | Lucknow | India |
| Shiv | Rajan | King George'S Medical University | Lucknow | India |
| Vijay | Kumar | King George'S Medical University | Lucknow | India |
| Abhinav | Arun Sonkar | King George’S Medical University | Lucknow | India |
| Ahmad | Ozair | King George’S Medical University | Lucknow | India |
| Vinay | Suresh | King George’S Medical University | Lucknow | India |
| Sargun | Virk | Dayanand Medical College And Hospital | Ludhiana | India |
| Mohan | Narasimhan | Meenakshi Mission Hospital & Research Centre | Madurai | India |
| Ramesh | Ardhanari | Meenakshi Mission Hospital & Research Centre | Madurai | India |
| Srinivasan | Ramachandran | Meenakshi Mission Hospital & Research Centre | Madurai | India |
| Divakar | Jain | Kokilaben Dhirubhai Ambani Hospital | Mumbai | India |
| Jayapala | Reddy Velagala | Kokilaben Dhirubhai Ambani Hospital | Mumbai | India |
| Somnath | Chattopadhyay | Kokilaben Dhirubhai Ambani Hospital | Mumbai | India |
| Charishma | Vodyala | Kokilaben Dhirubhai Ambani Hospital & Medical Research Institute | Mumbai | India |
| Jayapala Reddy | Velagala | Kokilaben Dhirubhai Ambani Hospital & Medical Research Institute | Mumbai | India |
| Kanchan | Motwani | Kokilaben Dhirubhai Ambani Hospital & Medical Research Institute | Mumbai | India |
| Ramlal | Prajapati | Seth G.S Medical College And K.E.M Hospital | Mumbai | India |
| Shruti | Tilak | Seth G.S Medical College And K.E.M Hospital | Mumbai | India |
| Varun | Bansal | Seth G.S Medical College And K.E.M Hospital | Mumbai | India |
| Raja | Kalayarasan | Jawaharlal Institute Of Postgraduate Medical Education & Research (Jipmer) | Puducherry | India |
| Suryabhan | Bhalerao | Jupiter Hospital , Baner Pune | Pune | India |
| Induchoodan | P. S | Government Medical College, Thiruvananthapuram | Thiruvananthapuram | India |
| Meer M. | Chisthi | Government Medical College, Thiruvananthapuram | Thiruvananthapuram | India |
| Nizarudeen | A | Government Medical College, Thiruvananthapuram | Thiruvananthapuram | India |
| Abdul | Latheef | Goverment Medical College,Trivandrum | Trivandrum | India |
| Induprabha | Yadev | Goverment Medical College,Trivandrum | Trivandrum | India |
| R. C. | Sreekumar | Goverment Medical College,Trivandrum | Trivandrum | India |
| Induprabha | Yadev | Medical College Trivandrum | Trivandrum | India |
| Viswanathan | KV | Medical College Trivandrum | Trivandrum | India |
| Durgatosh | Pandey | Tata Memorial Centre, Varanasi | Varanasi | India |
| Mayank | Tripathi | Tata Memorial Centre, Varanasi | Varanasi | India |
| Ahmad | Fathi Fuadi | Diponegoro University / Kariadi General Hospital | Semarang | Indonesia |
| Erik | Prabowo | Diponegoro University / Kariadi General Hospital | Semarang | Indonesia |
| Ali H. | Abbood | Al-Sader Teaching Hospital, An Najaf, Iraq | An Najaf | Iraq |
| Hayder | Hammoodi | Al-Sader Teaching Hospital, An Najaf, Iraq | An Najaf | Iraq |
| Maytham Aqeel | Al-juaifari | Al-Sader Teaching Hospital, An Najaf, Iraq | An Najaf | Iraq |
| Ali | Al-Isawi | Al-Hilla Teaching Hospital | Babil | Iraq |
| Sarah | Al-Tekreeti | Al-Kindy Teaching Hospital | Baghdad | Iraq |
| Mustafa | Al-Ogaili | Imamein Kadmein Teaching Hospital | Baghdad | Iraq |
| Hashim Talib | Hashim | University Of Baghdad, College Of Medicine | Baghdad | Iraq |
| Eran | Sadot | ​Rabin Medical Center | Petah Tikva | Israel |
| Roy | Apel | ​Rabin Medical Center | Petah Tikva | Israel |
| Omri | Sulimani | ​Rabin Medical Center | Petah Tikva | Israel |
| Evgeny | Solomonov | Ziv Medical Center | Safed | Israel |
| Ortal | Itzhaki | Shamir Medical Center | Zerifin | Israel |
| Ron | Lavy | Shamir Medical Center | Zerifin | Israel |
| Zahar | Shapira | Shamir Medical Center | Zerifin | Israel |
| Daniele | Nicolini | Division Of Hepatobiliary, Pancreatic And Transplant Unit | Ancona | Italy |
| Marco | Vivarelli | Division Of Hepatobiliary, Pancreatic And Transplant Unit | Ancona | Italy |
| MD | Roberta Rossi | Division Of Hepatobiliary, Pancreatic And Transplant Unit | Ancona | Italy |
| Federico | Mocchegiani | Ospedali Riuniti Ancona, Università Politecnica Delle Marche | Ancona | Italy |
| Riccardo | Memeo | Miulli Hospital | Bari | Italy |
| Leonardo | Vincenti | Policlinico Di Bari | Bari | Italy |
| Salvatore | Fedele | Policlinico Di Bari | Bari | Italy |
| Valeria | Andriola | Policlinico Di Bari | Bari | Italy |
| Angela | Gurrado | University Of Bari "Aldo Moro" Medical School | Bari | Italy |
| Giovanna | Di Meo | University Of Bari "Aldo Moro" Medical School | Bari | Italy |
| Mario | Testini | University Of Bari "Aldo Moro" Medical School | Bari | Italy |
| Vincenzo | Neri | University Of Foggia | Bari | Italy |
| Andrea | Zironda | Asst Papa Giovanni Xxiii | Bergamo | Italy |
| Arianna | Trizzino | Asst Papa Giovanni Xxiii | Bergamo | Italy |
| Domenico | Pinelli | Asst Papa Giovanni Xxiii | Bergamo | Italy |
| Michele | Colledan | Asst Papa Giovanni Xxiii | Bergamo | Italy |
| Paolo | Pizzini | Asst Papa Giovanni Xxiii | Bergamo | Italy |
| Riccardo | Cirelli | Asst Papa Giovanni Xxiii | Bergamo | Italy |
| M. | Masetti | Irccs - Azienda Ospedaliera Universitaria Di Bologna | Bologna | Italy |
| Matteo | Zanello | Irccs - Azienda Ospedaliera Universitaria Di Bologna | Bologna | Italy |
| Elio | Jovine | University Alma Mater Studiorum Bologna | Bologna | Italy |
| Laura | Mastrangelo | University Alma Mater Studiorum Bologna | Bologna | Italy |
| Raffaele | Lombardi | University Alma Mater Studiorum Bologna | Bologna | Italy |
| Riccardo | Casadei | University Of Bologna | Bologna | Italy |
| Anna | Malpaga | Central Hospital Of Bolzano | Bolzano | Italy |
| Antonio | Frena | Central Hospital Of Bolzano | Bolzano | Italy |
| Stefan | Patauner | Central Hospital Of Bolzano | Bolzano | Italy |
| Michele | Ciola | Regional Hospital Bolzano/Bozen | Bolzano | Italy |
| Jacopo | Andreuccetti | Asst Spedali Civili Of Brescia | Brescia | Italy |
| Alberto | Manzoni | Fondazione Poliambulanza Instituto Ospedaliero | Brescia | Italy |
| Mohammad | Abu Hilal | Fondazione Poliambulanza Instituto Ospedaliero | Brescia | Italy |
| Nine | de Graaf | Fondazione Poliambulanza Instituto Ospedaliero | Brescia | Italy |
| Marie Sophie | Alfano | Spedali Civili Di Brescia, Università Degli Studi Di Brescia | Brescia | Italy |
| Sarah | Molfino | Spedali Civili Di Brescia, Università Degli Studi Di Brescia | Brescia | Italy |
| Gian Luca | Baiocchi | University of Brescia, ASST Cremona | Brescia | Italy |
| Adolfo | Pisanu | Cagliari University Hospital | Cagliari | Italy |
| Alfredo | Mellano | Fpo- Ircc - Candiolo | Candiolo | Italy |
| Mario Virgilio | Papa | Aorn Caserta | Caserta | Italy |
| Isidoro | Di Carlo | University Of Catania | Catania | Italy |
| Marcello | Donati | University Of Catania | Catania | Italy |
| Michela | Zanatta | University Of Catania | Catania | Italy |
| Prof. | Francesco Basile | University Of Catania | Catania | Italy |
| Adelmo | Antonucci | Sant'Anna Hospital | Como | Italy |
| Davide | Papis | Sant'Anna Hospital | Como | Italy |
| Marina | Pighin | Sant'Anna Hospital | Como | Italy |
| Andrea | Celotti | Uo Chirurgia Generale - Asst Cremona | Cremona | Italy |
| Diego | Sasia | Santa Croce And Carle Hospital, Cuneo | Cuneo | Italy |
| Fabrizio | Allisiardi | Santa Croce And Carle Hospital, Cuneo | Cuneo | Italy |
| Felice | Borghi | Santa Croce And Carle Hospital, Cuneo | Cuneo | Italy |
| Francesca | Maione | Santa Croce And Carle Hospital, Cuneo | Cuneo | Italy |
| Giorgio | Giraudo | Santa Croce And Carle Hospital, Cuneo | Cuneo | Italy |
| Marco | Migliore | Santa Croce And Carle Hospital, Cuneo | Cuneo | Italy |
| Sara | Salomone | Santa Croce And Carle Hospital, Cuneo | Cuneo | Italy |
| Stefano | Giaccardi | Santa Croce And Carle Hospital, Cuneo | Cuneo | Italy |
| Valentina | Testa | Santa Croce And Carle Hospital, Cuneo | Cuneo | Italy |
| Marco | Giacometti | San Biagio Hospital | Domodossola | Italy |
| Sandro | Zonta | San Biagio Hospital | Domodossola | Italy |
| Antonio | Taddei | Careggi University Hospital, University Of Florence | Firenze | Italy |
| Matteo | Risaliti | Careggi University Hospital, University Of Florence | Firenze | Italy |
| Paolo | Muiesan | Careggi University Hospital, University Of Florence | Firenze | Italy |
| Irene | Urciuoli | Azienda Ospedaliero-Universitaria Careggi | Florence | Italy |
| Lapo | Bencini | Azienda Ospedaliero-Universitaria Careggi | Florence | Italy |
| Luca | Moraldi | Azienda Ospedaliero-Universitaria Careggi | Florence | Italy |
| Alessandro | Anastasi | Ospedale San Giovanni Di Dio | Florence | Italy |
| Giuseppe | Canonico | Ospedale San Giovanni Di Dio | Florence | Italy |
| Tommaso | Nelli | Ospedale San Giovanni Di Dio | Florence | Italy |
| Giuseppe | Lo Storto | Ospedali Riuniti Foggia | Foggia | Italy |
| Fabrizio | D'Acapito | Ausl Romagna, Morgagni-Pierantoni Hospital | Forlì | Italy |
| Giorgio | Ercolani | Ausl Romagna, Morgagni-Pierantoni Hospital | Forlì | Italy |
| Leonardo | Solaini | Ausl Romagna, Morgagni-Pierantoni Hospital | Forlì | Italy |
| Alessandro | Cucchetti | Morgagni-Pierantoni Hospital | Forlì | Italy |
| Andrea | Gardini | Morgagni-Pierantoni Hospital | Forlì | Italy |
| Carlo Alberto | Pacilio | Morgagni-Pierantoni Hospital | Forlì | Italy |
| Andrea | Barberis | Galliera Hospital | Genoa | Italy |
| Marco | Filauro | Galliera Hospital | Genoa | Italy |
| Franco | De Cian | Ospedale Policlinico S. Martino | Genoa | Italy |
| Roberto | Valente | Ospedale Policlinico S. Martino | Genoa | Italy |
| Stefano | Didomenico | Ospedale Policlinico S. Martino | Genoa | Italy |
| Francesco Saverio | Papadia | Policlinico San Martino | Genoa | Italy |
| Stefano | Di Domenico | Policlinico San Martino | Genoa | Italy |
| Raffaele | De Rosa | University of Genoa | Genoa | Italy |
| Andrea | Massobrio | University Of Genoa | Genoa | Italy |
| Stefano | Scabini | IRCCS Ospedale Policlinico San Martino | Genoa | Italy |
| Giacomo | Carganico | University of Genoa | Genova | Italy |
| Beatrice | Pessia | San Salvatore Hospital , University Of L'Aquila | L'Aquila | Italy |
| Federico | Sista | San Salvatore Hospital , University Of L'Aquila | L'Aquila | Italy |
| Mario | Schietroma | San Salvatore Hospital , University Of L'Aquila | L'Aquila | Italy |
| Marcello G. | Spampinato | Division Of Hpb Surgery - "Vito Fazzi" Hospital | Lecce | Italy |
| Stefano | Garritano | Division Of Hpb Surgery - "Vito Fazzi" Hospital | Lecce | Italy |
| Stefano | D'Ugo | Division Of Hpb Surgery - "Vito Fazzi" Hospital | Lecce | Italy |
| Tiziana | Marchese | Vito Fazzi Hospital | Lecce | Italy |
| Edoardo | Saladino | Ao Papardo | Messina | Italy |
| Giuseppe | Cuticone | Ao Papardo | Messina | Italy |
| Nino | Gullà | Ao Papardo | Messina | Italy |
| Alfonso | Recordare | Dell'Angelo Hospital | Mestre | Italy |
| Rubina | Palumbo | Dell'Angelo Hospital | Mestre | Italy |
| Alessandro | Giani | Asst Grande Ospedale Metropolitano Niguarda | Milan | Italy |
| Giovanni | Ferrari | Asst Grande Ospedale Metropolitano Niguarda | Milan | Italy |
| Michele | Mazzola | Asst Grande Ospedale Metropolitano Niguarda | Milan | Italy |
| Daniele | Dondossola | Fondazione Irccs Ca' Granda Ospedale Maggiore Policlinico | Milan | Italy |
| Giorgio | Rossi | Fondazione Irccs Ca' Granda Ospedale Maggiore Policlinico | Milan | Italy |
| Lucio | Caccamo | Fondazione Irccs Ca' Granda Ospedale Maggiore Policlinico | Milan | Italy |
| Alessandro | Zerbi | Humanitas Research Hospital | Milan | Italy |
| Gennaro | Nappo | Humanitas Research Hospital and Humanitas University. | Milan | Italy |
| Marco | Montorsi | Humanitas University | Milan | Italy |
| Jorgelina | Coppa | Istituto Nazionale Tumori | Milan | Italy |
| Michele | Droz dit Busset | Istituto Nazionale Tumori | Milan | Italy |
| Vincenzo | Mazzaferro | Istituto Nazionale Tumori | Milan | Italy |
| Albert | Troci | Ospedale Luigi Sacco | Milan | Italy |
| Alice | Frontali | Ospedale Luigi Sacco | Milan | Italy |
| Michele | Crespi | Ospedale Luigi Sacco | Milan | Italy |
| Caterina | Baldi | Ospedale San Carlo Borromeo, Milano | Milan | Italy |
| Laura | Benuzzi | Ospedale San Carlo Borromeo, Milano | Milan | Italy |
| Francesco | Ferrara | San Carlo Borromeo Hospital, University Of Milano | Milan | Italy |
| Marco | Stella | San Carlo Borromeo Hospital, University Of Milano | Milan | Italy |
| Gabriele | Capurso | San Raffaele Hospital | Milan | Italy |
| Massimo | Falconi | San Raffaele Hospital | Milan | Italy |
| Domenico | Tamburrino | San Raffaele Hospital Irccs | Milan | Italy |
| Fabrizio | Di Benedetto | University Of Modena And Reggio Emilia | Modena | Italy |
| Paolo | Magistri | University Of Modena And Reggio Emilia | Modena | Italy |
| Roberto | Ballarin | University Of Modena And Reggio Emilia | Modena | Italy |
| Giacomo | Zanus | Ospedale Treviso-Universita Padova | Mogliano Veneto | Italy |
| Marco | Brizzolari | Ospedale Treviso-Universita Padova | Mogliano Veneto | Italy |
| Fabio | Uggeri | University Of Milan-Bicocca | Monza | Italy |
| Luca | Gianotti | University Of Milan-Bicocca | Monza | Italy |
| Marco | Cereda | University Of Milan-Bicocca | Monza | Italy |
| Daniele | Ferraro | Aorn Antonio Cardarelli | Naples | Italy |
| Alessandro | Iacomino | Aorn Cardarelli | Naples | Italy |
| Daniele | Ferraro | Aorn Cardarelli | Naples | Italy |
| Donatella | Pisaniello | Aorn Cardarelli | Naples | Italy |
| Giovanni | Vennarecci | Aorn Cardarelli | Naples | Italy |
| Donatella | Pisaniello | Cardarelli Hospital -Naples | Naples | Italy |
| Gianluca | Rompianesi | Federico Ii University Hospital | Naples | Italy |
| Roberto Ivan | Troisi | Federico Ii University Hospital | Naples | Italy |
| Renato | Patrone | Irccs Pascale Napoli Int | Naples | Italy |
| Andrea | Belli | Istituto Nazionale Tumori - Irccs - Fondazione G. Pascale | Naples | Italy |
| Francesco | Izzo | Istituto Nazionale Tumori - Irccs - Fondazione G. Pascale | Naples | Italy |
| Raffaele | Palaia | Istituto Nazionale Tumori - Irccs - Fondazione G. Pascale | Naples | Italy |
| Marte | Gianpaolo | Ospedale Del Mare, Asl Na 1, Naples | Naples | Italy |
| Pietro | Maida | Ospedale Del Mare, Asl Na 1, Naples | Naples | Italy |
| Tammaro | Pasquale | Ospedale Del Mare, Asl Na 1, Naples | Naples | Italy |
| Domenico | Bassi | Univeristà Degli Studi Di Padova | Padova | Italy |
| Umberto | Cillo | Univeristà Degli Studi Di Padova | Padova | Italy |
| Lucia | Moletta | University Of Padova, Clinica Chirurgica 3° | Padova | Italy |
| Cosimo | Sperti | University Of Padova, Clinica Chirurgica 3° | Padova | Italy |
| Simone | Serafini | University Of Padova, Clinica Chirurgica 3° | Padova | Italy |
| Anna Caterina | Milanetto | University Of Padua | Padova | Italy |
| Claudio | Pasquali | University Of Padua | Padova | Italy |
| Francesca | Tolin | Veneto Institute Of Oncology Iov – Irccs | Padova | Italy |
| Mario | Gruppo | Veneto Institute Of Oncology Iov – Irccs | Padova | Italy |
| Ottavia | De Simoni | Veneto Institute Of Oncology Iov – Irccs | Padova | Italy |
| Salvatore | Buscemi | University of Palermo | Palermo | Italy |
| Marco V. | Marino | Azienda Ospedaliera Ospedali Riuniti Villa Sofia-Cervello | Palermo | Italy |
| Mario | Giuffrida | Parma University Hospital | Parma | Italy |
| Raffaele | Dallavalle | Parma University Hospital | Parma | Italy |
| Francesca | Calabretto | University Of Pavia | Pavia | Italy |
| Lorenzo | Cobianchi | University Of Pavia | Pavia | Italy |
| Luigi | Pugliese | University Of Pavia | Pavia | Italy |
| Alessandro | Giardino | Pederzoli Hospital | Peschiera Del Garda | Italy |
| Giovanni | Butturini | Pederzoli Hospital | Peschiera Del Garda | Italy |
| Paolo | Regi | Pederzoli Hospital | Peschiera Del Garda | Italy |
| Emanuele Federico | Kauffmann | Division Of General And Transplant Surgery | Pisa | Italy |
| Gregorio | Di Franco | University Of Pisa | Pisa | Italy |
| Luca | Morelli | University Of Pisa | Pisa | Italy |
| Niccolò | Furbetta | University Of Pisa | Pisa | Italy |
| Niccoló | Napoli | University Of Pisa | Pisa | Italy |
| Ugo | Boggi | University Of Pisa | Pisa | Italy |
| Enrico | Pinotti | Policlinico San Pietro | Ponte San Pietro | Italy |
| Mauro | Montuori | Policlinico San Pietro | Ponte San Pietro | Italy |
| Antonio | Giuliani | Aor San Carlo, Unit Of General And Emergency Surgery | Potenza | Italy |
| Maria Lucia | Izzo | Aor San Carlo, Unit Of General And Emergency Surgery | Potenza | Italy |
| Nicola | Zanini | Ausl Romagna | Rimini | Italy |
| Luigi | Veneroni | Ospedale Infermi Di Rimini | Rimini | Italy |
| Marco | Giordano | Ospedale Infermi Di Rimini | Rimini | Italy |
| Gian Marco | Palini | Ospedale Infermi Rimini | Rimini | Italy |
| Gianluca | Garulli | Ospedale Infermi Rimini | Rimini | Italy |
| Vincenzo | La Vaccara | Fondazione Policlinico Universitario Campus Bio-Medico | Rome | Italy |
| Agostino Maria | de Rose | Foundation "Policlinico Universitario A. Gemelli", Catholic University, Irccs | Rome | Italy |
| Felice | Giuliante | Foundation "Policlinico Universitario A. Gemelli", Catholic University, Irccs | Rome | Italy |
| Francesco | Ardito | Foundation "Policlinico Universitario A. Gemelli", Catholic University, Irccs | Rome | Italy |
| Andrea | Mingoli | Policlinico Umberto I Sapienza University Of Rome | Rome | Italy |
| Paolo | Sapienza | Policlinico Umberto I Sapienza University Of Rome | Rome | Italy |
| Pierfrancesco | Lapolla | Policlinico Umberto I Sapienza University Of Rome | Rome | Italy |
| Niccolo | Petrucciani | Sapienza University | Rome | Italy |
| Alessandra | Cossa | Sapienza University of Rome | Rome | Italy |
| Alessandro | Coppola | Sapienza University of Rome | Rome | Italy |
| Elena | Belloni | Sapienza University Of Rome | Rome | Italy |
| Giuseppe | Nigri | Sapienza University Of Rome | Rome | Italy |
| Giuseppe | Tisone | Tor Vergata University | Rome | Italy |
| Roberta | Angelico | Tor Vergata University | Rome | Italy |
| Tommaso Maria | Manzia | Tor Vergata University | Rome | Italy |
| Damiano | Caputo | University Campus Bio-Medico Of Rome | Rome | Italy |
| Salomone | Di Saverio | Madonna del Soccorso Hospital, AST 5 ASUR Marche | San Benedetto del tronto Ascoli Piceno | Italy |
| Alberto | Porcu | Azienda Ospedaliero Universitaria di Sassari | Sassari | Italy |
| Teresa | Perra | Azienda Ospedaliero Universitaria di Sassari | Sassari | Italy |
| Claudio | Feo | University Of Sassari | Sassari | Italy |
| Giulia | Deiana | University Of Sassari | Sassari | Italy |
| Lodovico | Sartarelli | Santa Annunziata Hospital Taranto - University Of Bologna | Taranto | Italy |
| Salvatore | Pisconti | Santa Annunziata Hospital Taranto - University Of Bologna | Taranto | Italy |
| Valeria | Tonini | Santa Annunziata Hospital Taranto - University Of Bologna | Taranto | Italy |
| Damiano | Patrono | Azienda Ospedaliero Universitaria Città Della Salute E Della Scienza Torino | Torino | Italy |
| Francesco | Moro | Azienda Ospedaliero Universitaria Città Della Salute E Della Scienza Torino | Torino | Italy |
| Luca | Grasso | Azienda Ospedaliero Universitaria Città Della Salute E Della Scienza Torino | Torino | Italy |
| Alberto | Brolese | APSS - Azienda Provinciale per i Servizi Sanitari | Trento | Italy |
| Francesco | Ciarleglio | APSS - Azienda Provinciale per i Servizi Sanitari | Trento | Italy |
| Stefano | Marcucci | APSS - Azienda Provinciale per i Servizi Sanitari | Trento | Italy |
| Cristina | Nistri | 1 Chirurgia Ospedale di Treviso | Treviso | Italy |
| Marco | Massani | 1 Chirurgia Ospedale di Treviso | Treviso | Italy |
| Stecca | Tommaso | Azienda ULSS2 Marca Trevigiana, Ospedale Ca’ Foncello Treviso | Treviso | Italy |
| Elisa | Galasso | 4 Chirurgia, Ospedale Di Treviso; Università Di Padova | Treviso | Italy |
| Giacomo | Zanus | 4 Chirurgia, Ospedale Di Treviso; Università Di Padova | Treviso | Italy |
| Marco | Brizzolari | 4 Chirurgia, Ospedale Di Treviso; Università Di Padova | Treviso | Italy |
| Maurizio | Romano | 4 Chirurgia, Ospedale Di Treviso; Università Di Padova | Treviso | Italy |
| Serena | Rossi | 4 Chirurgia, Ospedale Di Treviso; Università Di Padova | Treviso | Italy |
| Simone | Novello | 4 Chirurgia, Ospedale Di Treviso; Università Di Padova | Treviso | Italy |
| Alessandro | Ferrero | Ospedale Mauriziano | Turin | Italy |
| Serena | Langella | Ospedale Mauriziano | Turin | Italy |
| Serena | Armentano | Ospedale Mauriziano | Turin | Italy |
| Nadia | Russolillo | Ospedale Mauriziano | Turin | Italy |
| Lorenzin | Dario | Università Degli Studi Di Udine, Asufc Ospedale Santa Maria Della Misericordia | Udine | Italy |
| Sergio | Intini | Università Degli Studi Di Udine, Asufc Ospedale Santa Maria Della Misericordia | Udine | Italy |
| Terrosu | Giovanni | Università Degli Studi Di Udine, Asufc Ospedale Santa Maria Della Misericordia | Udine | Italy |
| Alfonso Giovanni | Recordare | Ospedale Dell'Angelo, Venezia | Venice | Italy |
| Giovanni | Pirozzolo | Ospedale Dell'Angelo, Venezia | Venice | Italy |
| Rubina | Palumbo | Ospedale Dell'Angelo, Venezia | Venice | Italy |
| Francesco | Calabrese | Ospedale "Castelli", Verbania - Asl Vco | Verbania | Italy |
| Giorgio | Querini | Ospedale "Castelli", Verbania - Asl Vco | Verbania | Italy |
| Sandro | Zonta | Ospedale "Castelli", Verbania - Asl Vco | Verbania | Italy |
| Andrea | Caneparo | Ospedale Unico Plurisede Asl Vco | Verbania-Domodossola | Italy |
| Marco | Giacometti | Ospedale Unico Plurisede Asl Vco | Verbania-Domodossola | Italy |
| Maura | De Francesco | Ospedale Unico Plurisede Asl Vco | Verbania-Domodossola | Italy |
| Alberto | Balduzzi | University Hospital of Verona | Verona | Italy |
| Alfredo | Guglielmi | University Hospital of Verona | Verona | Italy |
| Andrea | Ruzzenente | University Hospital of Verona | Verona | Italy |
| Calogero | Iacono | University Hospital of Verona | Verona | Italy |
| Edoardo | Poletto | University Hospital of Verona | Verona | Italy |
| Giovanni | Marchegiani | University Hospital of Verona | Verona | Italy |
| Giulia | Isa | University Hospital of Verona | Verona | Italy |
| Giuseppe | Malleo | University Hospital of Verona | Verona | Italy |
| Laura | Alaimo | University Hospital of Verona | Verona | Italy |
| Roberto | Salvia | University Hospital of Verona | Verona | Italy |
| Simone | Conci | University Hospital of Verona | Verona | Italy |
| Stefano Francesco | Crinò | University Hospital of Verona | Verona | Italy |
| Tommaso | Campagnaro | University Hospital of Verona | Verona | Italy |
| Isabella | Frigerio | Pederzoli Clinic | Verona | Italy |
| Francesco | De Marchi | San Bortolo Hospital | Vicenza | Italy |
| Michele | Bonomo | San Bortolo Hospital | Vicenza | Italy |
| Sara | Napetti | San Bortolo Hospital | Vicenza | Italy |
| Alice | Frontali | ASST-Brianza, Vimercate HospitalVia Santi Cosma e Damiano | Vimercate | Italy |
| Andrea | Chierici | ASST-Brianza, Vimercate HospitalVia Santi Cosma e Damiano | Vimercate | Italy |
| Christian | Cotsoglou | ASST-Brianza, Vimercate HospitalVia Santi Cosma e Damiano | Vimercate | Italy |
| Elson | Gjoni | ASST-Brianza, Vimercate HospitalVia Santi Cosma e Damiano | Vimercate | Italy |
| Sissi | Paleino | ASST-Brianza, Vimercate HospitalVia Santi Cosma e Damiano | Vimercate | Italy |
| Stefano | Granieri | ASST-Brianza, Vimercate HospitalVia Santi Cosma e Damiano | Vimercate | Italy |
| Daisuke | Hashimoto | Kansai Medical University | Hirakata | Japan |
| Sohei | Satoi | Kansai Medical University | Hirakata | Japan |
| Tomohisa | Yamamoto | Kansai Medical University | Hirakata | Japan |
| Kenichiro | Uemura | Hiroshima University | Hiroshima | Japan |
| Ippei | Matsumoto | Kindai University | Osaka-Sayama | Japan |
| Keiko | Kamei | Kindai University | Osaka-Sayama | Japan |
| Hiromitsu | Maehira | Shiga University of Medical Science | Otsu | Japan |
| Masaji | Tani | Shiga University of Medical Science | Otsu | Japan |
| Satoshi | Hirano | Hokkaido University Faculty Of Medicine | Sapporo | Japan |
| Toru | Nakamura | Hokkaido University Faculty Of Medicine | Sapporo | Japan |
| Toshimichi | Asano | Hokkaido University Faculty Of Medicine | Sapporo | Japan |
| Keiichi | Akahoshi | Tokyo Medical And Dental University | Tokyo | Japan |
| Minoru | Tanabe | Tokyo Medical And Dental University | Tokyo | Japan |
| Takeshi | Ishii | Tokyo Medical And Dental University | Tokyo | Japan |
| Mohamed H. | Al Saffaf | King Abdullah University Hospital | Allramtha | Jordan |
| Mohammad | Al Hamoud | King Abdullah University Hospital | Allramtha | Jordan |
| Rahaf | Khattab | King Abdullah University Hospital | Allramtha | Jordan |
| Subhi | Alissawi | Al-Basheer Hospital | Amman | Jordan |
| Anas | Hassouneh | Albashir Hospital | Amman | Jordan |
| Basma | Al-Maadani | Hashemite University | Amman | Jordan |
| Mahmoud | Bilal Alali | Hashemite University | Amman | Jordan |
| Quasi | Ahmad Hmdan | Hashemite University | Amman | Jordan |
| Aiman | Obed | Jordan Hospital | Amman | Jordan |
| Dania | Nijadat | Jordan University Hospital | Amman | Jordan |
| Khayry | Al-Shami | Jordan University Hospital | Amman | Jordan |
| Manar | Al-shami | Jordan University Hospital | Amman | Jordan |
| Maram | Mohsen | Jordan University Hospital | Amman | Jordan |
| Rozana | Al-Mallah | Jordan University Hospital | Amman | Jordan |
| Safa' | Albaba | Jordan University Of Science And Technology | Amman | Jordan |
| Mohammad | Theab | Jordanian Royal Medical Services | Amman | Jordan |
| Noor | Massadeh | Jordanian Royal Medical Services | Amman | Jordan |
| Reem Abdelrahman | Khader Theab | Jordanian Royal Medical Services | Amman | Jordan |
| Salah | Wardeh | Jordanian Royal Medical Services | Amman | Jordan |
| Salahwardeh |  | Jordanian Royal Medical Services | Amman | Jordan |
| Subhi | Zahi Alissawi | Jordanian Royal Medical Services | Amman | Jordan |
| Anas | Mardini | Jordanian Royal Medical Services | Amman | Jordan |
| Hamza | Ayman Abdelhalem Arabiyat | Jordanian Royal Medical Services | Amman | Jordan |
| Hamza | Arabiyat | Jordanian Royal Medical Services | Amman | Jordan |
| Abdelkareem | Al-Hyari | King Hussein Cancer Center | Amman | Jordan |
| Hazim | Ababneh | King Hussein Medical Center | Amman | Jordan |
| Mo'taz | Fawzat Naffa' | King Hussein Medical Center | Amman | Jordan |
| Mohammad | Musallam Buwaitel | King Hussein Medical Center | Amman | Jordan |
| Ayat | Al-Mekhlafi | The University Of Jordan | Amman | Jordan |
| Hebah | Rababa | The University Of Jordan | Amman | Jordan |
| Mohammed | Alzoubi | The University Of Jordan | Amman | Jordan |
| Almuatasim | Khamees | V.N Karazin Kharkov National University | Amman | Jordan |
| Amro | Abuleil | V.N Karazin Kharkov National University | Amman | Jordan |
| Sari | Almiani | V.N Karazin Kharkov National University | Amman | Jordan |
| Khaled | Obeidat | Jordan University Of Science And Technology | Irbid | Jordan |
| Zouhair | Amarin | Jordan University Of Science And Technology | Irbid | Jordan |
| Abdulaziz | Al-Samawi | King Abdullah University Hospital | Irbid | Jordan |
| Amr A. | Al Hammoud | King Abdullah University Hospital | Irbid | Jordan |
| H. | Hammad | King Abdullah University Hospital | Irbid | Jordan |
| Maeen | Ali Alkhadem | King Abdullah University Hospital | Irbid | Jordan |
| Mahmoud R. | Mahafdah | King Abdullah University Hospital | Irbid | Jordan |
| Mohamed | AlSaffaf | King Abdullah University Hospital | Irbid | Jordan |
| Mohamed | Alsabah | King Abdullah University Hospital | Irbid | Jordan |
| Mohammad | Bani Hani | King Abdullah University Hospital | Irbid | Jordan |
| Mohammad A. | AL Hamoud | King Abdullah University Hospital | Irbid | Jordan |
| Mohammad W. | Alzghoul | King Abdullah University Hospital | Irbid | Jordan |
| Rahaf N. | Khattab | King Abdullah University Hospital | Irbid | Jordan |
| Saeed K. | Shumrakh | King Abdullah University Hospital | Irbid | Jordan |
| Ansam | Rababah | Yarmouk University | Irbid | Jordan |
| Heyam | Alghazo | Yarmouk University | Irbid | Jordan |
| Tasneem | Rababah | Yarmouk University | Irbid | Jordan |
| Almu'Atasim | Khamees | Yarmouk University - Irbid | Irbid | Jordan |
| Sajeda | Awadi | Yarmouk University - Irbid | Irbid | Jordan |
| Sarah | Al Sharie | Yarmouk University - Irbid | Irbid | Jordan |
| Ahmad | Abdelnoor | Royal Medical Services Hopsital | Jordan | Jordan |
| Duaa | Mohammad Shaout | New Zarqa Governmental Hospital | Zarqa | Jordan |
| Qais | Omarieh | New Zarqa Governmental Hospital | Zarqa | Jordan |
| Sara | Yassir Abu-Ghazal | New Zarqa Governmental Hospital | Zarqa | Jordan |
| Sara | Abu-Ghazal | New Zarqa Governmental Hospital | Zarqa | Jordan |
| Dua'A | Shaout | Zarqa New Governmental Hospital | Zarqa | Jordan |
| Sara | Yasser Abughazal | Zarqa New Governmental Hospital | Zarqa | Jordan |
| Ildar | Fakhradiyev | S.D. Asfendiyarov Kazakh National Medical University | Almaty | Kazakhstan |
| Shynar | Tanabayeva | S.D. Asfendiyarov Kazakh National Medical University | Almaty | Kazakhstan |
| Timur | Saliev | S.D. Asfendiyarov Kazakh National Medical University | Almaty | Kazakhstan |
| Ibragim | Issabekov | National Research Oncology Center | Nur-Sultan | Kazakhstan |
| Zhanat | Spatayev | National Research Oncology Center | Nur-Sultan | Kazakhstan |
| Ho-Seong | Han | Seoul National University Bundang Hospital | Seongnam | Korea |
| Yeongsoo | Jo | Seoul National University Bundang Hospital | Seongnam | Korea |
| Haralds | Plaudis | Riga East Clinical University Hospital | Riga | Latvia |
| Katrina Deja | Martinsone | Riga East Clinical University Hospital | Riga | Latvia |
| Kristaps | Atstupens | Riga East Clinical University Hospital | Riga | Latvia |
| Mohamad | Jawad | American University Of Beirut | Beirut | Lebanon |
| Mohamad | Khalife | American University Of Beirut | Beirut | Lebanon |
| Walid | Ghazi Faraj | American University Of Beirut | Beirut | Lebanon |
| Fatoom | Alowjali | Banghazi Aljala Hospital | Banghazi | Libya |
| Mohammed N. | Albaraesi | Benghazi Medical Center | Benghazi | Libya |
| Wafa | Aldressi | Benghazi Medical Center | Benghazi | Libya |
| Aihab | Benamwor | Naitional Insititution _ Misurata | Misrata | Libya |
| Suhil | Ben Ali | National Cancer Institute Misrata | Misrata | Libya |
| Suhil | Saleh | National Cancer Institute Misrata | Misrata | Libya |
| Abdulhadi | Alshatshat | National Cancer Institute | Misurata | Libya |
| Eman | Younes | National Cancer Institute Of Sabratah | Sabratah | Libya |
| Eman | Younes | National Cancer Institute Of Sabratah | Sabratah | Libya |
| Reyad | Ekhmaj | National Cancer Institute Of Sabratah | Sabratah | Libya |
| Sumayyah | Bahroun | National Cancer Institute Of Sabratah | Sabratah | Libya |
| Sumayyah Ghayth | Bahroun | National Cancer Institute Of Sabratah | Sabratah | Libya |
| Reyad | Ekhmaj | National Cancer Institute Sabratah | Sabratah | Libya |
| Sumayyah | Ghayth Bahroun | National Cancer Institute Sabratah | Sabratah | Libya |
| Dania | Burgan | National Cancer Institute, Sabratha - Libya | Sabratha | Libya |
| Hajer | Abd Alhamed Aalem | National Cancer Institute, Sabratha - Libya | Sabratha | Libya |
| Ramadan Kamoka | El Hussain | National Cancer Institute, Sabratha - Libya | Sabratha | Libya |
| Marwa | Morgom | Alestiklal Hospital At Libya | Tripoli | Libya |
| Entisar | Alshareea | Cenral Hospital | Tripoli | Libya |
| Amera | Ali Dakheel Ellafi | El Khadra Hospital | Tripoli | Libya |
| Shoukrie | Shoukrie | El Khadra Hospital | Tripoli | Libya |
| Taha | Mahmoud Omar Shamakhi | El Khadra Hospital | Tripoli | Libya |
| Elham | Bareig | Medical Care Clinic | Tripoli | Libya |
| Malek | Abusannuga | Medical Care Clinic | Tripoli | Libya |
| Abdulbari | Dkhakhni | Tripoli Central Hospital | Tripoli | Libya |
| Ahmed | Abdualla Gerwash | Tripoli Central Hospital | Tripoli | Libya |
| Ahmed | Gerwash | Tripoli Central Hospital | Tripoli | Libya |
| Ali | Zawia | Tripoli Central Hospital | Tripoli | Libya |
| Eman | Othman | Tripoli Central Hospital | Tripoli | Libya |
| Muhannud | Binnawara | Tripoli Central Hospital | Tripoli | Libya |
| Sabriya | Juma Ali Abdalsalam | Tripoli Central Hospital | Tripoli | Libya |
| Sarah | Aljamal | Tripoli Central Hospital | Tripoli | Libya |
| Sultan | Ahmeed | Tripoli Central Hospital | Tripoli | Libya |
| Wegdan | Ibrahim Almabrouk Khalil | Tripoli Central Hospital | Tripoli | Libya |
| Wegdan | Khalel | Tripoli Central Hospital | Tripoli | Libya |
| Tasneem | Faraj | Tripoli Central Hospital - Tch | Tripoli | Libya |
| Fras | Elhajdawe | Tripoli Medical Center | Tripoli | Libya |
| Marwa | Emhemed | Tripoli Medical Center | Tripoli | Libya |
| Osama | Salem | Tripoli Medical Center | Tripoli | Libya |
| Eman | Abdulwahed | Tripoli University | Tripoli | Libya |
| Wegdan | Khalil | Tripoli University | Tripoli | Libya |
| Heba | Rhuma | Tripoli University Hospital | Tripoli | Libya |
| Mohamed | Alsori | Tripoli University Hospital | Tripoli | Libya |
| Tahani | Mustafa | Tripoli University Hospital | Tripoli | Libya |
| Sofian | Albarouni | Tripoli University/Libya | Tripoli | Libya |
| Ahmed | Albishti | University of Tripoli | Tripoli | Libya |
| Muhammed | Elhadi | University of Tripoli | Tripoli | Libya |
| Taha | Elkhuja | University of Tripoli | Tripoli | Libya |
| Ahmed | Msherghi | University Of Tripoli Faculty Of Medicine | Tripoli | Libya |
| Najat | Shaban Ben Hasan | Zliten Medical Centre | Zliten | Libya |
| Hayat | Ben Hasan | Zliten Medical College Of Asmarya University | Zliten | Libya |
| Najat | Ben Hasan | Zliten Medical College Of Asmarya University | Zliten | Libya |
| Antanas | Gulbinas | Lithuanian University Of Health Sciences | Kaunas | Lithuania |
| Giedrius | Barauskas | Lithuanian University Of Health Sciences | Kaunas | Lithuania |
| Povilas | Ignatavicius | Lithuanian University Of Health Sciences | Kaunas | Lithuania |
| Romualdas | Riauka | Lithuanian University Of Health Sciences | Kaunas | Lithuania |
| Tomas | Vanagas | Lithuanian University Of Health Sciences | Kaunas | Lithuania |
| Algirdas | Slepavicius | Klaipeda University Hospital | Klaipeda | Lithuania |
| Jonas | Jurgaitis | Klaipeda University Hospital | Klaipeda | Lithuania |
| Sarunas | Dailidenas | Klaipeda University Hospital | Klaipeda | Lithuania |
| Vitalijus | Eismontas | Klaipeda University Hospital | Klaipeda | Lithuania |
| Vytenis | Mikutaitis | Klaipeda University Hospital | Klaipeda | Lithuania |
| Algirdas | Šlepavičius | Klaipėda University Hospital | Klaipėda | Lithuania |
| Jonas | Jurgaitis | Klaipėda University Hospital | Klaipėda | Lithuania |
| Vytenis | Mikutaitis | Klaipėda University Hospital | Klaipėda | Lithuania |
| Audrius | Dulskas | National Cancer Institute | Vilnius | Lithuania |
| Justas | Kuliavas | National Cancer Institute | Vilnius | Lithuania |
| Laura | Aniukstyte | National Cancer Institute | Vilnius | Lithuania |
| Audrius | Sileikis | Vilnius University Hospital Santaros Klinikos | Vilnius | Lithuania |
| Aiste | Gulla | Vilnius University Hospital Santaros Klinikos | Vilnius | Lithuania |
| Audrius | Šileikis | Vilnius University Hospital Santaros Klinikos | Vilnius | Lithuania |
| Jaroslav | Tumas | Vilnius University Hospital Santaros Klinikos | Vilnius | Lithuania |
| Kestutis | Strupas | Vilnius University Hospital Santaros Klinikos | Vilnius | Lithuania |
| Marius | Petrulionis | Vilnius University Hospital Santaros Klinikos | Vilnius | Lithuania |
| Mindaugas | Kvietkauskas | Vilnius University Hospital Santaros Klinikos | Vilnius | Lithuania |
| Prof.Kestutis | Strupas | Vilnius University Hospital Santaros Klinikos | Vilnius | Lithuania |
| Deblasi | Vito | Centre Hospitalier Du Luxembourg | Luxembourg | Luxembourg |
| Edoardo | Rosso | Centre Hospitalier Du Luxembourg | Luxembourg | Luxembourg |
| Jih Huei | Tan | Hsajb | Johor | Malaysia |
| Andee Dzulkarnaen | Zakaria | School of Medical Sciences & Hospital USM, Universiti Sains Malaysia | Kota Bharu, Kelantan | Malaysia |
| Ikhwan Sani | Mohamad | School of Medical Sciences & Hospital USM, Universiti Sains Malaysia | Kota Bharu, Kelantan | Malaysia |
| Leow | Voon Meng | School of Medical Sciences & Hospital USM, Universiti Sains Malaysia | Kota Bharu, Kelantan | Malaysia |
| Teoh | Zhan Huai | School of Medical Sciences & Hospital USM, Universiti Sains Malaysia | Kota Bharu, Kelantan | Malaysia |
| Firdaus | Hayati | Universiti Malaysia Sabah | Kota Kinabalu | Malaysia |
| Harivinthan | Sellappan | Universiti Malaysia Sabah | Kota Kinabalu | Malaysia |
| Thanesh | Kumar Maiyauen | Universiti Malaysia Sabah | Kota Kinabalu | Malaysia |
| Azlanudin | Azman | Universiti Kebangsaan Malaysia Medical Centre | Kuala Lumpur | Malaysia |
| Ian | Chik | Universiti Kebangsaan Malaysia Medical Centre | Kuala Lumpur | Malaysia |
| Zamri | Zuhdi | Universiti Kebangsaan Malaysia Medical Centre | Kuala Lumpur | Malaysia |
| Boon | Yoong | University Malaya Medical Centre | Kuala Lumpur | Malaysia |
| Koh | Peng Soon | University Malaya Medical Centre | Kuala Lumpur | Malaysia |
| Koong | Jun Kit | University Malaya Medical Centre | Kuala Lumpur | Malaysia |
| Boon | Koon Yoong | University Of Malaya | Kuala Lumpur | Malaysia |
| Jun | Kit Koong | University Of Malaya | Kuala Lumpur | Malaysia |
| Peng Soon | Koh | University Of Malaya | Kuala Lumpur | Malaysia |
| Aini | Ibrahim | Universiti Malaysia Sarawak (Unimas ) | Kuching, Sarawak | Malaysia |
| Nik | Azim Nik Abdullah | Universiti Malaysia Sarawak (Unimas ) | Kuching, Sarawak | Malaysia |
| Jin | Bong | Sunway Medical Center | Petaling Jaya | Malaysia |
| Shahi | Ghani | Tree Top Hospital | Male' | Maldives |
| Carlos | Florez Zorrilla | Centro Medico Nacional 20 De Noviembre | Mexico | Mexico |
| Miguel | Charco Cruz | Centro Medico Nacional 20 De Noviembre | Mexico | Mexico |
| Andre | Moguel Valladares | Instituto Nacional De Ciencias Medicas Y Nutricion Salvador Zubiran | Mexico | Mexico |
| Ismael | Dominguez-Rosado | Instituto Nacional De Ciencias Medicas Y Nutricion Salvador Zubiran | Mexico | Mexico |
| Alejandro Eduardo | Padilla Rosciano | National Cáncer Institute | Mexico | Mexico |
| Garcia-Herrera | Sebastian | National Cancer Institute Mexico | Mexico | Mexico |
| Javier | Melchor-Ruan | National Cancer Institute Mexico | Mexico | Mexico |
| Juan Sebastian | Garcia-Herrera | National Cancer Institute Mexico | Mexico | Mexico |
| Erdene | Sandag | Mongolian National University of Medical Sciences | Ulaanbaatar | Mongolia |
| Sarnai | Erdene | Mongolian National University of Medical Sciences | Ulaanbaatar | Mongolia |
| Sergelen | Orgoi | Mongolian National University of Medical Sciences | Ulaanbaatar | Mongolia |
| Moniba | Korch | University Hospital Mohammed Vi | Marrakech | Morocco |
| Ahmed Sami | Boutti | University Hospital Mohammed Vi | Marrakech | Morocco |
| yassmine | boumzebra | University Hospital Mohammed Vi | Marrakech | Morocco |
| Yassmine | Boumzebra | Cadi Ayad University | Marrakesh | Morocco |
| Fatine | Hourri | University Hospital Center Mohamed 6 | Marrakesh | Morocco |
| Iltimass | Gouazar | University Hospital Center Mohamed 6 | Marrakesh | Morocco |
| Wafae | Ait Belaid | University Hospital Center Mohamed 6 | Marrakesh | Morocco |
| Badr | Serji | Oncology Hospital Hasssan Ii. Mohammed Ist University | Oujda | Morocco |
| Bouhout | Tarik | Oncology Hospital Hasssan Ii. Mohammed Ist University | Oujda | Morocco |
| El | Harroudi Tijani | Oncology Hospital Hasssan Ii. Mohammed Ist University | Oujda | Morocco |
| Aziz | Zentar | Military Universitaire Hospital Med V Rabat Morocco | Rabat | Morocco |
| Abdelilah | Ghannam | National Institute Of Oncology, Mohammed V University In Rabat | Rabat | Morocco |
| Ahmed | Bounaim | National Institute Of Oncology, Mohammed V University In Rabat | Rabat | Morocco |
| Amine | Souadka | National Institute Of Oncology, Mohammed V University In Rabat | Rabat | Morocco |
| Amine | Benkabbou | National Institute Of Oncology, Mohammed V University In Rabat | Rabat | Morocco |
| Brahim | El Ahmadi | National Institute Of Oncology, Mohammed V University In Rabat | Rabat | Morocco |
| Houmada | Amina | National Institute Of Oncology, Mohammed V University In Rabat | Rabat | Morocco |
| Lahnaoui | Oumayma | National Institute Of Oncology, Mohammed V University In Rabat | Rabat | Morocco |
| Laila | Amrani | National Institute Of Oncology, Mohammed V University In Rabat | Rabat | Morocco |
| Mohammed Anass | Majbar | National Institute Of Oncology, Mohammed V University In Rabat | Rabat | Morocco |
| Raouf | Mohsine | National Institute Of Oncology, Mohammed V University In Rabat | Rabat | Morocco |
| Reda | Elhassouni | National Institute Of Oncology, Mohammed V University In Rabat | Rabat | Morocco |
| Sabrillah | Echiguer | National Institute Of Oncology, Mohammed V University In Rabat | Rabat | Morocco |
| Zakaria | Belkhadir | National Institute Of Oncology, Mohammed V University In Rabat | Rabat | Morocco |
| Abdulrashid Pueya | Nashidengo | Windhoek Central Academic Hospital/University Of Namibia School Of Medicine | Windhoek | Namibia |
| Francis | Quayson | Windhoek Central Academic Hospital/University Of Namibia School Of Medicine | Windhoek | Namibia |
| John | Abebrese | Windhoek Central Academic Hospital/University Of Namibia School Of Medicine | Windhoek | Namibia |
| Pueya | Nashidengo | Windhoek Central Academic Hospital/University Of Namibia School Of Medicine | Windhoek | Namibia |
| Krishna | Mohan Adhikari | Tribhuvan University Teaching Hospital, Institute Of Medicine | Kathmandu | Nepal |
| Paleswan Joshi | Lakhey | Tribhuvan University Teaching Hospital, Institute Of Medicine | Kathmandu | Nepal |
| Ramesh | Singh Bhandari | Tribhuvan University Teaching Hospital, Institute Of Medicine | Kathmandu | Nepal |
| Marc G. | Besselink | Amsterdam University Medical Cente | Amsterdam | Netherlands |
| Matthanja | Bieze | Amsterdam University Medical Cente | Amsterdam | Netherlands |
| Simone | Augustinus | Amsterdam University Medical Cente | Amsterdam | Netherlands |
| Olivier | Busch | Amsterdam University Medical Cente | Amsterdam | Netherlands |
| B.K. | Pranger | University of Groningen and University Medical Center Groningen | Groningen | Netherlands |
| F.J.H. | Hoogwater | University of Groningen and University Medical Center Groningen | Groningen | Netherlands |
| J.M. | Klaase | University of Groningen and University Medical Center Groningen | Groningen | Netherlands |
| M. | Meerdink | University of Groningen and University Medical Center Groningen | Groningen | Netherlands |
| M.W. | Nijkamp | University of Groningen and University Medical Center Groningen | Groningen | Netherlands |
| V.E. | de Meijer | University of Groningen and University Medical Center Groningen | Groningen | Netherlands |
| Bas | Groot Koerkamp | Erasmus MC University Medical Center | Rotterdam | Netherlands |
| Casper H.J. | van Eijck | Erasmus MC University Medical Center | Rotterdam | Netherlands |
| Jacob L. | Van Dam | Erasmus MC University Medical Center | Rotterdam | Netherlands |
| Louise | Barbier | Auckland City Hospital - Auckland District Health Board | Auckland | New Zealand |
| Peter | Johnston | Auckland City Hospital - Auckland District Health Board | Auckland | New Zealand |
| Richard | Babor | Auckland City Hospital - Auckland District Health Board | Auckland | New Zealand |
| Michael Jen Jie | Chu | Auckland District Health Board | Auckland | New Zealand |
| Tiffany | Oliver | Auckland District Health Board | Auckland | New Zealand |
| Daniel | Wen | North Shore Hospital/ University Of Auckland | Auckland | New Zealand |
| Jonathan | Koea | North Shore Hospital/ University Of Auckland | Auckland | New Zealand |
| Jonathan | Koea | North Shore Hospital/ University Of Auckland | Auckland | New Zealand |
| Lisa | Brown | North Shore Hospital/ University Of Auckland | Auckland | New Zealand |
| Sanket | Srinivasa | North Shore Hospital/ University Of Auckland | Auckland | New Zealand |
| Adam | Bartlett | University Of Auckland | Auckland | New Zealand |
| John | Windsor | University Of Auckland | Auckland | New Zealand |
| Peter | Carr-Boyd | University Of Auckland | Auckland | New Zealand |
| Vanshay | Bindra | Waitemata District Health Board | Auckland | New Zealand |
| Andrea | Cross | Christchurch Hospital | Christchurch | New Zealand |
| Saxon | Connor | Christchurch Hospital | Christchurch | New Zealand |
| Todd | Hore | Christchurch Hospital | Christchurch | New Zealand |
| Ashok | Gunawardene | Waikato District Health Board | Hamilton | New Zealand |
| Fraser | Welsh | Waikato District Health Board | Hamilton | New Zealand |
| Monique | Mahadik | Waikato District Health Board | Hamilton | New Zealand |
| Alexandra | Gordon | Midcentral District Health Board | Palmerston North | New Zealand |
| Jeremy | Rossaak | Tauranga Hospital | Tauranga | New Zealand |
| Ademola | Adeyeye | Afe Babalola University Ado-Ekiti(Abuad) | Ado-Ekiti | Nigeria |
| Elizabeth | Enoch | Afe Babalola University Ado-Ekiti(Abuad) | Ado-Ekiti | Nigeria |
| Victor | Kayode-Nissi | Afe Babalola University Ado-Ekiti(Abuad) | Ado-Ekiti | Nigeria |
| Henry | Abiyere | Afe Babalola University Ado-Ekiti(Abuad) | Adoekiti | Nigeria |
| Olusegun | Alatise | Obafemi Awolowo University | Ile Ife | Nigeria |
| Andrew | Okomayin | Irrua Specialist Teaching Hospital, Irrua | Irrua, Edo State | Nigeria |
| Clement | Odion | Irrua Specialist Teaching Hospital, Irrua | Irrua, Edo State | Nigeria |
| Esteem | Tagar | Irrua Specialist Teaching Hospital, Irrua | Irrua, Edo State | Nigeria |
| Abdulrahaman | Abba Sheshe | Aminu Kano Teaching Hospital | Kano | Nigeria |
| Abubakar Bala | Muhammad | Aminu Kano Teaching Hospital | Kano | Nigeria |
| Ibrahim Umar | Garzali | Aminu Kano Teaching Hospital | Kano | Nigeria |
| Peter | Ajayi | Lagos University Teaching Hospital | Lagos | Nigeria |
| Exhevit | Kadri | City General Hospital 8Th Of September | Skopje | North Macedonia |
| Salah | Al Jabri | Royal Hospital | Muscat | Oman |
| Yahya | Al Azri | Royal Hospital | Muscat | Oman |
| Khuwaja | Muahammad Inam Pal | Aga Khan University Hospital | Karachi | Pakistan |
| Tayyab | Siddiqui | Aga Khan University Hospital | Karachi | Pakistan |
| Usama | Waqar | Aga Khan University Hospital | Karachi | Pakistan |
| Usama | Waqar | Aga Khan University Hospital | Karachi | Pakistan |
| Ahmad | Areeb Chaudhry | Aga Khan University Hospital Karachi | Karachi | Pakistan |
| Jibran | Abbasy | Aga Khan University Hospital Karachi | Karachi | Pakistan |
| Muhammad | Osama Khan | Aga Khan University Hospital Karachi | Karachi | Pakistan |
| Syed | Shafqatullah | Jinnah Postgraduate Medical Center | Karachi | Pakistan |
| Muhammad Imran | Khokhar | Ameer Ul Deen Medical College, Pgmi, Lgh | Lahore | Pakistan |
| Ali | Akbar | King Edward Medical University Mayo Hospital Lahore | Lahore | Pakistan |
| Ameer | Afzal | King Edward Medical University Mayo Hospital Lahore | Lahore | Pakistan |
| Mohammad | Asghar | King Edward Medical University Mayo Hospital Lahore | Lahore | Pakistan |
| Sami | Ullah | Services Hospital Lahore | Lahore | Pakistan |
| Usman | Ismat Butt | Services Hospital Lahore | Lahore | Pakistan |
| Usman | Butt | Services Institute of Medical Sciences , Lahore | Lahore | Pakistan |
| Hassaan | Bari | Shauakat Khanam Memorial Cancer Hospital And Research Centre | Lahore | Pakistan |
| Bilal | Nabil Mohammad | Al-Hussein Government Hospital ,Beit Jala | Bethlehem | Palestine, State of |
| Mahmoud | Hameda | Al-Hussein Government Hospital ,Beit Jala | Bethlehem | Palestine, State of |
| Mustafa | Abu Jayyab | Alshifaa Hospital | Gaza | Palestine, State of |
| Asmaa | Hasan Mohammad Alzabadiah | Hebron Governmental hospital-Aliah Hospital | Hebron | Palestine, State of |
| Islam | Adam | Hebron Governmental hospital-Aliah Hospital | Hebron | Palestine, State of |
| Khalil | Abuzaina | Hebron Governmental hospital-Aliah Hospital | Hebron | Palestine, State of |
| Mohammad | Farid | Hebron Governmental hospital-Aliah Hospital | Hebron | Palestine, State of |
| Mohammad | Farid Mohammad Emar | Hebron Governmental hospital-Aliah Hospital | Hebron | Palestine, State of |
| Mohammad | Emar | Hebron Governmental hospital-Aliah Hospital | Hebron | Palestine, State of |
| Qusai | Zreqat | Hebron Governmental hospital-Aliah Hospital | Hebron | Palestine, State of |
| Rawand | Titi | Hebron Governmental hospital-Aliah Hospital | Hebron | Palestine, State of |
| Sarah | Ameen Idkiedek | Hebron Governmental hospital-Aliah Hospital | Hebron | Palestine, State of |
| Sarah | Amro | Hebron Governmental hospital-Aliah Hospital | Hebron | Palestine, State of |
| Shahd | Al-Qasrawi | Hebron Governmental hospital-Aliah Hospital | Hebron | Palestine, State of |
| Tabarak | Abedlnaser Almasri | Hebron Governmental hospital-Aliah Hospital | Hebron | Palestine, State of |
| Walaa Mohammed | Alnammourah | Hebron Governmental hospital-Aliah Hospital | Hebron | Palestine, State of |
| Gharam | Kiswani | Makased | Jerusalem | Palestine, State of |
| Raghida | Sinnokrot | Makased | Jerusalem | Palestine, State of |
| Zahrah | Abu Harb | Makased | Jerusalem | Palestine, State of |
| Hiba | Nafa'A | Al Najah University Hospital | Nablus | Palestine, State of |
| Lyana | Shtewi | Al Najah University Hospital | Nablus | Palestine, State of |
| Abrar | Omar Salah | Al-Najah University | Nablus | Palestine, State of |
| Aseef | B. A. Joma | Al-Najah University | Nablus | Palestine, State of |
| Sireen | Faraj | Al-Najah University | Nablus | Palestine, State of |
| Abdullah | Zitawi | An-Najah National University Hospital | Nablus | Palestine, State of |
| Ahmad | Jamal Dawood | An-Najah National University Hospital | Nablus | Palestine, State of |
| Ibraheem | Saadeh | An-Najah National University Hospital | Nablus | Palestine, State of |
| Alaa | Hmeedan | An-Najah National University Hospital Nnuh | Nablus | Palestine, State of |
| Motaz | Ayman Mahmoud Daraghmeh | An-Najah National University Hospital Nnuh | Nablus | Palestine, State of |
| Amani | Netham Atia Janajreh | Najah National University (Nnu) | Nablus | Palestine, State of |
| Fatima | Manassra | Najah National University (Nnu) | Nablus | Palestine, State of |
| Laya | Moayyad Ahmed Yassin | Najah National University (Nnu) | Nablus | Palestine, State of |
| Raya | Yassin | Najah National University (Nnu) | Nablus | Palestine, State of |
| Abrar | Omar Saleh | Rafedia Government Hospital | Nablus | Palestine, State of |
| Sireen | Mahmoud Faraj | Rafedia Government Hospital | Nablus | Palestine, State of |
| Abdallah S. | Sulaiman | Rafidia | Nablus | Palestine, State of |
| Zain | Khayyat | Rafidia | Nablus | Palestine, State of |
| Aseef B. A. | Joma | Rafidia Hospital | Nablus | Palestine, State of |
| Eman | Shawahni | Rafidia Hospital | Nablus | Palestine, State of |
| Abrar | Salah | Al Najah Hospital | Nabuls | Palestine, State of |
| Abdullatef | khader | Palestine Medical Complex | Ramallah | Palestine, State of |
| Ahlam | Hammoudeh | Palestine Medical Complex | Ramallah | Palestine, State of |
| Aram | Abdulhaq | Palestine Medical Complex | Ramallah | Palestine, State of |
| Reem | Alawna | Palestinian Medical Complex | Ramallah | Palestine, State of |
| Gilbert | Roman | Clinica Delgado | Lima | Peru |
| Javier | Targarona | Clinica Delgado | Lima | Peru |
| Rafael | Garatea Grau | Clínica Ricardo Palma | Lima | Peru |
| Raquel | Molina | Hospital Edgardo Rebagliati Martins | Lima | Peru |
| Cesar | Rodriguez Alegria | Hospital Nacional Edgardo Rebagliati Martins | Lima | Peru |
| Guillermo | Coayla | Hospital Nacional Edgardo Rebagliati Martins | Lima | Peru |
| Juan | Carlos Marcos Enriquez | Hospital Nacional Edgardo Rebagliati Martins | Lima | Peru |
| Juan Carlos | Marcos | Hospital Nacional Edgardo Rebagliati Martins | Lima | Peru |
| Alyssa Nicole | Hasiman | Makati Medical Center | Makati | Philippines |
| Catherine | Teh | Makati Medical Center | Makati | Philippines |
| Ruby | Cerdeño | Makati Medical Center | Makati | Philippines |
| Avril | David | Rizal Medical Center | Manila | Philippines |
| Ray I. | Sarmiento | Rizal Medical Center | Manila | Philippines |
| Ryan Ruel | Barroso | Rizal Medical Center | Manila | Philippines |
| Cenon | Alfonso | Ateneo School Of Medicine And Public Health, Dean | Pasig | Philippines |
| Dr. | Dante Ang | Ateneo School Of Medicine And Public Health, Dean | Pasig | Philippines |
| Amornetta | Casupang | National Kidney & Transplant Institute | Quezon City | Philippines |
| Monica | Mamuric | National Kidney & Transplant Institute | Quezon City | Philippines |
| Jose | Mari Jardinero | St Luke'S Medical Center | Quezon City | Philippines |
| Agata | Motyka | Jurasz University Hospital | Bydgoszcz | Poland |
| Marta | Flisińska | Jurasz University Hospital | Bydgoszcz | Poland |
| Stanisław | Pierściński | Jurasz University Hospital | Bydgoszcz | Poland |
| Slawomir | Mrowiec | Department Of Gastrointestinal Surgery, Medical University Of Silesia, Katowice, Poland. | Katowice | Poland |
| Justyna | Rymarowicz | Universiy Hospital | Krakow | Poland |
| Maciej | Matyja | Universiy Hospital | Krakow | Poland |
| Tomasz | Wikar | Universiy Hospital | Krakow | Poland |
| Marek | Sierzega | Jagiellonian University Medical College | Kraków | Poland |
| Michał | Pędziwiatr | Jagiellonian University Medical College | Kraków | Poland |
| Piotr | Richter | Jagiellonian University Medical College | Kraków | Poland |
| Adam | Durczynski | Medical University Of Lodz | Lodz | Poland |
| Konrad | Kosztowny | Medical University Of Lodz | Lodz | Poland |
| Wojciech | Ciesielski | Medical University Of Lodz | Lodz | Poland |
| Aleksander | Wardeszkiewicz | Medical University Of Lodz | Łódź | Poland |
| Krzysztof | Szwedziak | Medical University Of Lodz | Łódź | Poland |
| Michal | Wlazlak | Medical University Of Lodz | Łódź | Poland |
| Oliwia | Grzasiak | Medical University Of Lodz | Łódź | Poland |
| Patrycja | Szewczyk | Medical University Of Lodz | Łódź | Poland |
| Piotr | Hogendorf | Medical University Of Lodz | Łódź | Poland |
| Justyna | Wyroślak-Najs | Medical University of Lublin | Lublin | Poland |
| Karol | Rawicz-Pruszyński | Medical University of Lublin | Lublin | Poland |
| Katarzyna | Sędłak | Medical University of Lublin | Lublin | Poland |
| Michał | Solecki | Medical University of Lublin | Lublin | Poland |
| Wojciech | Polkowski | Medical University of Lublin | Lublin | Poland |
| Maciej | Słodkowski | Warsaw Medical University | Warsaw | Poland |
| Michał | Wierzchowski | Warsaw Medical University | Warsaw | Poland |
| Wojciech | Korcz | Warsaw Medical University | Warsaw | Poland |
| Lukasz | Nazarewski | Medical University of Warsaw | Warszawa | Poland |
| Oskar | Kornasiewicz | Medical University of Warsaw | Warszawa | Poland |
| Maria | Lopes | Centro Hospitalar E Universitário De Coimbra | Coimbra | Portugal |
| Rui Miguel | Martins | Ipoc | Coimbra | Portugal |
| Ruben | Martins | Chua | Faro | Portugal |
| Emanuel | Vigia | Hospital De Curry Cabral - Centro Hospitalar Universitário De Lisboa Central | Lisbon | Portugal |
| Donzília | Sousa Silva | Centro Hospitalar Universitário Do Porto | Porto | Portugal |
| José | Davide | Centro Hospitalar Universitário Do Porto | Porto | Portugal |
| Andre | Pereira | Hospital De São João | Porto | Portugal |
| Nadia | Tenreiro | Chtmad | Vila Real | Portugal |
| Tiago | Castro | Chtmad | Vila Real | Portugal |
| Reem | Eisa | El Demerdash Hospital (Cairo, Egypt), Hamad Hospital (Doha, Qatar) | Doha/Cairo | Qatar |
| Bogdan | Diaconescu | Carol Davila University Of Medicine And Pharmacy | Bucharest | Romania |
| Cezar | Ciubotaru | Carol Davila University Of Medicine And Pharmacy Bucharest, Emergency Hospital Of Bucharest | Bucharest | Romania |
| Ionut | Negoi | Carol Davila University Of Medicine And Pharmacy Bucharest, Emergency Hospital Of Bucharest | Bucharest | Romania |
| Valentina | Negoiță | Carol Davila University Of Medicine And Pharmacy Bucharest, Emergency Hospital Of Bucharest | Bucharest | Romania |
| Raluca | Bievel Radulescu | Emergency Hospital Prof Dr. “Agrippa Ionescu” | Bucharest | Romania |
| Nicolae | Bacalbașa | Fundeni Clinical Institute, Carol Davila University of Medicine and Pharmacy | Bucharest | Romania |
| Simona | Dima | Fundeni Clinical Institute, Carol Davila University of Medicine and Pharmacy | Bucharest | Romania |
| Traian | Dumitrascu | Fundeni Clinical Institute, Carol Davila University of Medicine and Pharmacy | Bucharest | Romania |
| Andrada | Spanu | Monza Hospital Bucharest | Bucharest | Romania |
| Mara | Mardare | Monza Hospital Bucharest | Bucharest | Romania |
| Octav | Ginghina | Monza Hospital Bucharest | Bucharest | Romania |
| Eduard | Catrina | Spitalul Clinic Dr. I. Cantacuzino | Bucharest | Romania |
| Iulian | Brezean | Spitalul Clinic Dr. I. Cantacuzino | Bucharest | Romania |
| Mihaela | Misca | Spitalul Clinic Dr. I. Cantacuzino | Bucharest | Romania |
| Mihaela | Vilcu | Spitalul Clinic Dr. I. Cantacuzino | Bucharest | Romania |
| Sorin | Aldoescu | Spitalul Clinic Dr. I. Cantacuzino | Bucharest | Romania |
| Sorin | Petrea | Spitalul Clinic Dr. I. Cantacuzino | Bucharest | Romania |
| Adrian | Bartos | ''Iuliu Hatieganu'' University Of Medicine And Pharmacy | Cluj-Napoca | Romania |
| Cioltean | Cristian Liviu | ''Iuliu Hatieganu'' University Of Medicine And Pharmacy | Cluj-Napoca | Romania |
| Ioana | Iancu | ''Iuliu Hatieganu'' University Of Medicine And Pharmacy | Cluj-Napoca | Romania |
| Sorin Traian | Barbu | "Iuliu Hatieganu" University Of Medicine & Pharmacy | Cluj-Napoca | Romania |
| Raluca | Bodea | Regional Institute Of Gastroenterology And Hepatology | Cluj-Napoca | Romania |
| Emil | Mois | Regional Institute Of Gastroenterology And Hepatology "O. Fodor" | Cluj-Napoca | Romania |
| Graur | Florin | Regional Institute Of Gastroenterology And Hepatology "O. Fodor" | Cluj-Napoca | Romania |
| Nadim | al Hajjar | Regional Institute Of Gastroenterology And Hepatology "O. Fodor" | Cluj-Napoca | Romania |
| Sergiu | Matei | University Hospital C.F.R. Cluj-Napoca, Romania | Cluj-Napoca | Romania |
| Florin | Zaharie | University Of Medicine And Pharmacy "Iuliu Hatieganu" | Cluj-Napoca | Romania |
| Viorel | Scripcariu | Univ.Of Medicine And Pharmacy Iasi Romania. | Iasi | Romania |
| Ana-Maria | Musina | University Of Medicine And Pharmacy Gr.T. Popa Iasi | Iasi | Romania |
| Cristian Ene | Roata | University Of Medicine And Pharmacy Gr.T. Popa Iasi | Iasi | Romania |
| Gabriel Mihali | Dimofte | University Of Medicine And Pharmacy Gr.T. Popa Iasi | Iasi | Romania |
| Natalia | Velenciuc | University Of Medicine And Pharmacy Gr.T. Popa Iasi | Iasi | Romania |
| Sorinel | Lunca | University Of Medicine And Pharmacy Gr.T. Popa Iasi | Iasi | Romania |
| Wee | Liam Ong | University Of Medicine And Pharmacy Gr.T. Popa Iasi | Iasi | Romania |
| Wee Liam | Ong | University Of Medicine And Pharmacy Gr.T. Popa Iasi | Iasi | Romania |
| Ciprian | Duta | Victor Babes University Of Medicine And Pharmacy Timisoara | Timisoara | Romania |
| Dan | Brebu | Victor Babes University Of Medicine And Pharmacy Timisoara | Timisoara | Romania |
| Vlad | Braicu | Victor Babes University Of Medicine And Pharmacy Timisoara | Timisoara | Romania |
| Alexander | Belyaev | Archangel Regional Clinical Hospital | Arkhangelsk | Russian Federation |
| Alexey | Popov | Archangel Regional Clinical Hospital | Arkhangelsk | Russian Federation |
| Anastasia | Batova | Archangel Regional Clinical Hospital | Arkhangelsk | Russian Federation |
| Anastasiia | Katysheva | First Clinical City Hospital Named After E. E. Volosevich | Arkhangelsk | Russian Federation |
| Denis | Mizgirev | First Clinical City Hospital Named After E. E. Volosevich | Arkhangelsk | Russian Federation |
| Liudmila | Neledova | First Clinical City Hospital Named After E. E. Volosevich | Arkhangelsk | Russian Federation |
| Boris | Duberman | Northern State Medical University | Arkhangelsk | Russian Federation |
| Andrey | Litvin | Immanuel Kant Baltic Federal University, Regional Clinical Hospital, Kaliningrad | Kaliningrad | Russian Federation |
| Artem | Pobelenko | Immanuel Kant Baltic Federal University, Regional Clinical Hospital, Kaliningrad | Kaliningrad | Russian Federation |
| Georgy | Kuznetsov | Immanuel Kant Baltic Federal University, Regional Clinical Hospital, Kaliningrad | Kaliningrad | Russian Federation |
| Igor | Khatkov | Moscow Clinical Scientific Center Named After A.S.Loginov | Moscow | Russian Federation |
| Pavel | Tyutyunnik | Moscow Clinical Scientific Center Named After A.S.Loginov | Moscow | Russian Federation |
| Roman | Izrailov | Moscow Clinical Scientific Center Named After A.S.Loginov | Moscow | Russian Federation |
| Arkady | Bedzhanyan | Petrovsky National Research Center Of Surgery | Moscow | Russian Federation |
| Konstantin | Petrenko | Petrovsky National Research Center Of Surgery | Moscow | Russian Federation |
| Mikhail | Bredikhin | Petrovsky National Research Center Of Surgery | Moscow | Russian Federation |
| Dr. | Garnik Shatverian | Petrovsky Russian Research Center For Surgery | Moscow | Russian Federation |
| Nikita | Chardarov | Petrovsky Russian Research Center For Surgery | Moscow | Russian Federation |
| Nikolay | Bagmet | Petrovsky Russian Research Center For Surgery | Moscow | Russian Federation |
| Vladimir | Lyadov | Russian Medical Academy Of Continuous Professional Education | Moscow | Russian Federation |
| Daniil | Mudryak | Sechenovskiy University | Moscow | Russian Federation |
| Ivan | Semenenko | Sechenovskiy University | Moscow | Russian Federation |
| Mark | Tokarev | Sechenovskiy University | Moscow | Russian Federation |
| Andrey | Kriger | Vishnevsky Center Of Surgery | Moscow | Russian Federation |
| Ayrat | Kaldarov | Vishnevsky Center Of Surgery | Moscow | Russian Federation |
| Gennady | Ivanov | Vorohobov Hospital | Moscow | Russian Federation |
| Denis | Kuchin | Privolzhsky District Medical Center | Nizhny Novgorod | Russian Federation |
| Gaik | Torgomyan | Privolzhsky District Medical Center | Nizhny Novgorod | Russian Federation |
| Vladimir | Zagainov | Privolzhsky District Medical Center | Nizhny Novgorod | Russian Federation |
| Vasili | Davydkin | National Research Mordovia State University | Saransk | Russian Federation |
| Andrey | Igorevich Baranov | Siberian State Medical University | Tomsk | Russian Federation |
| Evgeniy | Drozdov | Siberian State Medical University | Tomsk | Russian Federation |
| Li | Natalya Anatolievna | Siberian State Medical University | Tomsk | Russian Federation |
| Abakar | Abdullaev | Vladimir City Emergency Hospital | Vladimir | Russian Federation |
| Mahir | Gachabayov | Vladimir City Emergency Hospital | Vladimir | Russian Federation |
| Mohammed | Ghunaim | King Abdulaziz University Hospital | Jeddah | Saudi Arabia |
| Mohammed | Alharthi | King Abdulaziz University Hospital | Jeddah | Saudi Arabia |
| Murad | Aljiffry | King Abdulaziz University Hospital | Jeddah | Saudi Arabia |
| Marko | Bogdanovic | Clinic For Digestive Surgery, Clinical Center Of Serbia | Belgrade | Serbia |
| Marko | Zivanovic | Clinic For Digestive Surgery, Clinical Center Of Serbia | Belgrade | Serbia |
| Aleksandar | Bogdanovic | Clinical Center Of Serbia | Belgrade | Serbia |
| Daniel | Galun | Clinical Center Of Serbia | Belgrade | Serbia |
| Vladimir | Dugalic | Clinical Center Of Serbia | Belgrade | Serbia |
| Dragana | Arbutina | Clinical For Surgery Nikola Spasic City Hospital | Belgrade | Serbia |
| Ljiljana | Milic | Clinical For Surgery Nikola Spasic City Hospital | Belgrade | Serbia |
| Mihailo | Bezmarevic | Military Medical Academy | Belgrade | Serbia |
| Andrija | Antic | University Of Belgrade | Belgrade | Serbia |
| Dejan | Radenkovic | University Of Belgrade | Belgrade | Serbia |
| Igor | Ignjatovic | University Of Belgrade | Belgrade | Serbia |
| Predrag | Zdujic | University Of Belgrade | Belgrade | Serbia |
| Stefan | Kmezic | University Of Belgrade | Belgrade | Serbia |
| Aleksandar | Karamarkovic | Zvezdara University Medical Center | Belgrade | Serbia |
| Dragana | Arbutina | Zvezdara University Medical Center | Belgrade | Serbia |
| Jovan | Juloski | Zvezdara University Medical Center | Belgrade | Serbia |
| Radisav | Radulovic | Zvezdara University Medical Center | Belgrade | Serbia |
| Radosav | Radulović | Zvezdara University Medical Center | Belgrade | Serbia |
| Vladica | Cuk | Zvezdara University Medical Center | Belgrade | Serbia |
| Ljiljana | Jeremic | Clinical Center, University Of Nis | Nis | Serbia |
| Milan | Radojkovic | Clinical Center, University Of Nis | Nis | Serbia |
| Miroslav | Stojanovic | Clinical Center, University Of Nis | Nis | Serbia |
| Danica | Golijanin | Oncology Institute Of Vojvodina | Novi Sad | Serbia |
| Milana | Kresoja Ignjatovic | Oncology Institute Of Vojvodina | Novi Sad | Serbia |
| Mladjan | Protic | Oncology Institute Of Vojvodina | Novi Sad | Serbia |
| Adrian | Chiow | Changi General Hospital | Singapore | Singapore |
| Lee | Lip Seng | Changi General Hospital | Singapore | Singapore |
| Nita | Thiruchelvam | Changi General Hospital | Singapore | Singapore |
| Brian | Goh Kim Poh | Singapore General Hospital | Singapore | Singapore |
| Brian  K. P | Goh | Singapore General Hospital | Singapore | Singapore |
| Darren | Chua Wei Quan | Singapore General Hospital | Singapore | Singapore |
| Ye Xin | Koh | Singapore General Hospital | Singapore | Singapore |
| Blaž | Trotovšek | University Medical Centre Ljubljana | Ljubljana | Slovenia |
| Miha | Petrič | University Medical Centre Ljubljana | Ljubljana | Slovenia |
| Mihajlo | Djokić | University Medical Centre Ljubljana | Ljubljana | Slovenia |
| Ales | Tomazic | University Medical Centre, Faculty Of Medicine Ljubljana | Ljubljana | Slovenia |
| David | Badovinac | University Medical Centre, Faculty Of Medicine Ljubljana | Ljubljana | Slovenia |
| Emil | Loots | Entabeni Hospital | Durban | South Africa |
| Leanne | Prodehl | Charlotte Maxeke Johannesburg Academic Hospital | Johannesburg | South Africa |
| Mohammed | Uzayr Khan | Charlotte Maxeke Johannesburg Academic Hospital | Johannesburg | South Africa |
| Thomas | Marumo | Charlotte Maxeke Johannesburg Academic Hospital | Johannesburg | South Africa |
| John W.S. | Devar | Chris Hani Baragwanath Academic Hospital/University Of The Witwatersrand | Johannesburg | South Africa |
| Jones | Omoshoro-Jones | Chris Hani Baragwanath Academic Hospital/University Of The Witwatersrand | Johannesburg | South Africa |
| Zafar A. | Khan | Chris Hani Baragwanath Academic Hospital/University Of The Witwatersrand | Johannesburg | South Africa |
| Ben | Jugmohan | Sandton Mediclinic | Johannesburg | South Africa |
| Ana | Quiroga Valcarcel | Hospital Universitario Príncipe De Asturias | Alcalá De Henares | Spain |
| Belén | Matías García | Hospital Universitario Príncipe De Asturias | Alcalá De Henares | Spain |
| Javier | Mínguez | Hospital Universitario Príncipe De Asturias | Alcalá De Henares | Spain |
| Manuel | Marcello | Hospital Universitario Fundación Alcorcón | Alcorcón-Madrid | Spain |
| Jose | Ramia | Hospital General Universitario Alicante | Alicante | Spain |
| Antonio | Compañ | Universidad Miguel Hernández; Hospital Universitario Sant Joan D´Alacant | Alicante | Spain |
| Carlos | Fernandes | Universidad Miguel Hernández; Hospital Universitario Sant Joan D´Alacant | Alicante | Spain |
| Miguel | Morales | Universidad Miguel Hernández; Hospital Universitario Sant Joan D´Alacant | Alicante | Spain |
| Jose | Miguel Vargas Fernández | Hospital Universitario Torrecárdenas | Almería | Spain |
| Maria Del Mar | Rico-Morales | Hospital Universitario Torrecárdenas | Almería | Spain |
| Miguel | Ángel Lorenzo Liñán | Hospital Universitario Torrecárdenas | Almería | Spain |
| Joan | Figueras | “Sagrat Cor “ Hospital Universitari. | Barcelona | Spain |
| Ramon | Soliva | “Sagrat Cor “ Hospital Universitari. | Barcelona | Spain |
| Eugenia | Butori | Hospital Clinic, Barcelona | Barcelona | Spain |
| Constantino | Fondevila | Hospital Clinic, University Of Barcelona | Barcelona | Spain |
| Fabio | Ausania | Hospital Clinic, University of Barcelona, IDIBAPS | Barcelona | Spain |
| Belén | Martín | Hospital De La Santa Creu I Sant Pau | Barcelona | Spain |
| Manuel | Rodríguez | Hospital De La Santa Creu I Sant Pau | Barcelona | Spain |
| Santiago | Sánchez-Cabús | Hospital De La Santa Creu I Sant Pau | Barcelona | Spain |
| Patricia | Sánchez-Velázquez | Hospital Del Mar | Barcelona | Spain |
| Ana Belen | Martin Arnau | Hospital Sant Pau | Barcelona | Spain |
| Ramón | Soliva Domínguez | Hospital Universitari Sagrat Cor | Barcelona | Spain |
| Benedetto | Ielpo | University Hospital Del Mar | Barcelona | Spain |
| Fernando | Burdío Pinilla | University Hospital Del Mar | Barcelona | Spain |
| Maria | Castro | Hospital Universitario Puerta Del Mar | Cadiz | Spain |
| David | Padilla Valverde | Hospital General Universitario De Ciudad Real | Ciudad Real | Spain |
| Esther Pilar | García Santos | Hospital General Universitario De Ciudad Real | Ciudad Real | Spain |
| María | del Carmen Manzanares Campillo | Hospital General Universitario De Ciudad Real | Ciudad Real | Spain |
| Patricia | Ruiz | Cruces University Hospital | Cruces | Spain |
| Ernesto | Castro Gutierrez | Hospital Universitari De Girona Dr. Josep Trueta | Girona | Spain |
| Laia | Falgueras | Hospital Universitari De Girona Dr. Josep Trueta | Girona | Spain |
| Maria | Teresa Albiol Quer | Hospital Universitari De Girona Dr. Josep Trueta | Girona | Spain |
| Farah | Al Shwely | Hospital University Guadalajara | Guadalajara | Spain |
| Raquel Latorre | Fragua | Hospital University Guadalajara | Guadalajara | Spain |
| Daniel | Bejarano Gonzalez-Serna | University Hospital Juan Ramon Jimenez | Huelva | Spain |
| Marcos | Alba Valmorisco | University Hospital Juan Ramon Jimenez | Huelva | Spain |
| Pablo | Beltran-Miranda | University Hospital Juan Ramon Jimenez | Huelva | Spain |
| Juli | Busquets | Bellvitge University Hospital | L´Hospitalet De Llobregat; Barcelona | Spain |
| Lluis | Secanella | Bellvitge University Hospital | L´Hospitalet De Llobregat; Barcelona | Spain |
| Nuria | Pelaez | Bellvitge University Hospital | L´Hospitalet De Llobregat; Barcelona | Spain |
| Gabriel | Plaza | Hospital Insular De Gran Canaria | Las Palmas De Gran Canaria | Spain |
| Marta Lourdes | Gonzalez Duaigües | Hospital Universitari Arnau de Vilanova de Lleida | Lleida | Spain |
| Pablo | Muriel Álvarez | Hospital Universitari Arnau de Vilanova de Lleida | Lleida | Spain |
| Alfredo | Escartín | Hospital Universitari Arnau de Vilanova de Lleida | Lleida | Spain |
| Carmelo | Loinaz | "12 De Octubre" University Hospital | Madrid | Spain |
| Jana | Dziakova | Hospital Clinico San Carlos | Madrid | Spain |
| Sofia | de la Serna | Hospital Clinico San Carlos | Madrid | Spain |
| Elia | Pérez-Aguirre | Hospital Clínico San Carlos | Madrid | Spain |
| Iago | Justo | Hospital Universitario 12 De Octubre | Madrid | Spain |
| Jorge | Saavedra | Hospital Universitario La Paz Madrid Spain | Madrid | Spain |
| Jose | Castell Gomez | Hospital Universitario La Paz Madrid Spain | Madrid | Spain |
| Nuria Losa | Boñar | Hospital Universitario La Paz Madrid Spain | Madrid | Spain |
| Elena | Martín-Perez | Hospital Universitario La Princesa | Madrid | Spain |
| Marcello | Di Martino | Hospital Universitario La Princesa | Madrid | Spain |
| Ángela | de la Hoz Rogriguez | Hospital Universitario La Princesa | Madrid | Spain |
| Alberto | Marcacuzco | University Hospital 12 De Octubre | Madrid | Spain |
| Carlos | Jiménez-Romero | University Hospital 12 De Octubre | Madrid | Spain |
| Jorge F. | Roldán de la Rúa | University Hospital Virgen De La Victoria | Málaga | Spain |
| Luis C. | Hinojosa-Arco | University Hospital Virgen De La Victoria | Málaga | Spain |
| Miguel Ángel | Suárez-Muñoz | University Hospital Virgen De La Victoria | Málaga | Spain |
| David | Ferreras Martinez | University “Virgen De La Arrixaca”Hospital | Murcia | Spain |
| Francisco | Sanchez-Bueno | University “Virgen De La Arrixaca”Hospital | Murcia | Spain |
| Pedro | Gil Vazquez | University “Virgen De La Arrixaca”Hospital | Murcia | Spain |
| Alberto | Miyar de León | Central University Hospital Of Asturias - Huca | Oviedo | Spain |
| Elisa | Contreras Saiz | Central University Hospital Of Asturias - Huca | Oviedo | Spain |
| Lorena | Solar García | Central University Hospital Of Asturias - Huca | Oviedo | Spain |
| Ignacio | Gonzalez-Pinto | Huca, University Of Oviedo | Oviedo | Spain |
| José Carlos | Rodríguez-Pino | University Hospital Son Espases | Palma De Mallorca | Spain |
| Juan José | Segura-Sampedro | University Hospital Son Espases | Palma De Mallorca | Spain |
| Rafa | Morales | University Hospital Son Espases | Palma De Mallorca | Spain |
| Rafael | Morales-Soriano | University Hospital Son Espases | Palma De Mallorca | Spain |
| Fernando | Rotellar | Clinica Universidad De Navarra | Pamplona | Spain |
| Gabriel | Zozaya | Clinica Universidad De Navarra | Pamplona | Spain |
| Pablo | Martí-Cruchaga | Clinica Universidad De Navarra | Pamplona | Spain |
| Jaime | López-Sánchez | Hospital Universitario De Salamanca. Universidad De Salamanca | Salamanca | Spain |
| Luis | Muñoz-Bellvis | Hospital Universitario De Salamanca. Universidad De Salamanca | Salamanca | Spain |
| Angel | Cuadrado | Hu Infanta Sofía | San Sebastián De Los Reyes Madrid | Spain |
| Irene | ortega | Hu Infanta Sofía | San Sebastián De Los Reyes Madrid | Spain |
| Rocio | Fernández | Hu Infanta Sofía | San Sebastián De Los Reyes Madrid | Spain |
| Daniel | Díaz Gómez | Hospital Quiron Salud Infanta Luisa | Sevilla | Spain |
| Valle | Vera | Hospital Quiron Salud Infanta Luisa | Sevilla | Spain |
| Javier Padillo | Padillo | University Hospital Virgen del Rocío. | Seville | Spain |
| Juan | Bellido Luque | Virgen De La Macarena Hospital | Seville | Spain |
| Erik | Ilacer Millan | Joan Xxiii University Hospital Of Tarragona | Tarragona | Spain |
| Rosa | Jorba | Joan Xxiii University Hospital Of Tarragona | Tarragona | Spain |
| María Isabel | García-Domingot | Hospital Universitari Mutua Terrassa | Terrassa | Spain |
| Carlos | Redondo | Hospital General De Valencia | Valencia | Spain |
| Dra. | Míriam Cantos | Hospital General De Valencia | Valencia | Spain |
| Enrique | Artigues | Hospital General De Valencia | Valencia | Spain |
| Carlos | Domingo-Del Pozo | Hospital Universitario Dr. Peset | Valencia | Spain |
| Carmen | Payá Llorente | Hospital Universitario Dr. Peset | Valencia | Spain |
| Sergio | Navarro Martínez | Hospital Universitario Dr. Peset | Valencia | Spain |
| Cristina | Ballester Ibáñez | Hospital Universitario La Fe | Valencia | Spain |
| Javier | Maupoey Ibáñez | Hospital Universitario La Fe | Valencia | Spain |
| Rafael | López Andujar | Hospital Universitario La Fe | Valencia | Spain |
| Dimitri | Dorcaratto | Hospital Clinico Universitario Valencia | València | Spain |
| Elena | Muñoz Forner | Hospital Clinico Universitario Valencia | València | Spain |
| Marina | Garces-Albir | Hospital Clinico Universitario Valencia | València | Spain |
| Juan | Beltran de Heredia | Valladolid University Clinic Hospital | Valladolid | Spain |
| Mario | Montes-Manrique | Valladolid University Clinic Hospital | Valladolid | Spain |
| Mario | Rodriguez-Lopez | Valladolid University Clinic Hospital | Valladolid | Spain |
| Alejandro | Serrablo | Miguel Servet University Hospital | Zaragoza | Spain |
| Daniel | Milian | Miguel Servet University Hospital | Zaragoza | Spain |
| Pablo | Ruiz-Quijano | Miguel Servet University Hospital | Zaragoza | Spain |
| Sandra | Paterna-Lopez | Miguel Servet University Hospital | Zaragoza | Spain |
| Arinda | Dharmapala | Faculty Of Medicine, University Of Peradeniya, Sri Lanka | Kandy | Sri Lanka |
| B. K. | Dassanayake | Faculty Of Medicine, University Of Peradeniya, Sri Lanka | Kandy | Sri Lanka |
| K. B. | Galketiya | Faculty Of Medicine, University Of Peradeniya, Sri Lanka | Kandy | Sri Lanka |
| Ahmed | Mohamed Ibrahim | University of Gadarif Faculty of Medicine and Health Sciences | Gadarif | Sudan |
| Hytham | Hamid | Ibn Sina Hospital | Khartoum | Sudan |
| Nassir | Alhaboob | Ibn Sina Hospital | Khartoum | Sudan |
| Abdelfatah | Abdelmageed | Omdurman Islamic University | Khartoum | Sudan |
| Samah | Suliman Osman Taha | Omdurman Islamic University | Khartoum | Sudan |
| Caroline | Vilhav | Inst. Of Clinical Sciences | Gothenburg | Sweden |
| Johanna | Hansson Wennerblom | Inst. Of Clinical Sciences | Gothenburg | Sweden |
| Svein Olav | Bratlie | Inst. Of Clinical Sciences | Gothenburg | Sweden |
| Bergthor | Bjornsson | University Hospital Of Linkoping | Linköping | Sweden |
| Linda | Lundgren | University Hospital Of Linkoping | Linköping | Sweden |
| Per | Sandström | University Hospital Of Linkoping | Linköping | Sweden |
| Bobby | Tingstedt | University Of Lund | Lund | Sweden |
| Roland | Andersson | University Of Lund | Lund | Sweden |
| Bodil | Andersson | University Of Lund | Lund | Sweden |
| Caroline | Williamsson | University Of Lund | Lund | Sweden |
| Ernesto | Sparrelid | Karolinska Institutet | Stockholm | Sweden |
| Marcus | Holmberg | Karolinska Institutet | Stockholm | Sweden |
| Poya | Ghorbani | Karolinska Institutet | Stockholm | Sweden |
| Ioannis | Gkekas | Umeå University | Umeå | Sweden |
| Christoph | Kuemmerli | Clarunis, St. Clara Hospital And University Hospital | Basel | Switzerland |
| Martin | Bolli | Clarunis, St. Clara Hospital And University Hospital | Basel | Switzerland |
| Andreas | Andreou | Inselspital, University Bern | Bern | Switzerland |
| Anna Silvia | Wenning | Inselspital, University Bern | Bern | Switzerland |
| Beat | Gloor | Inselspital, University Bern | Bern | Switzerland |
| Andrea | Peloso | University Hospital Of Geneva | Geneva | Switzerland |
| Christian | Toso | University Hospital Of Geneva | Geneva | Switzerland |
| Graziano | Oldani | University Hospital Of Geneva | Geneva | Switzerland |
| Beat | Moeckli | University Of Geneva | Geneva | Switzerland |
| Charles-Henri | Wassmer | University Of Geneva | Geneva | Switzerland |
| Alessandra | Cristaudi | Ospedale Regionale Di Lugano | Lugano | Switzerland |
| Majno-Hurst | Pietro | Ospedale Regionale Di Lugano | Lugano | Switzerland |
| Pietro Edoardo | Majno-Hurst | Ospedale Regionale Di Lugano | Lugano | Switzerland |
| Raffaello | Roesel | Ospedale Regionale Di Lugano | Lugano | Switzerland |
| Fariba | Abbassi | Kantonsspital St.Gallen | St. Gallen | Switzerland |
| Ignazio | Tarantino | Kantonsspital St.Gallen | St. Gallen | Switzerland |
| Thomas | Steffen | Kantonsspital St.Gallen | St. Gallen | Switzerland |
| Carlo | Ferrari | Hirslanden Clinics Zurich | Zurich | Switzerland |
| Jan | Schmidt | Hirslanden Clinics Zurich | Zurich | Switzerland |
| Olga | Meier | Hirslanden Clinics Zurich | Zurich | Switzerland |
| Markus | Weber | Triemli Hospital Zurich | Zurich | Switzerland |
| Stefan | Gutknecht | Triemli Hospital Zurich | Zurich | Switzerland |
| Jan Philipp | Jonas | University Hospital Zurich | Zurich | Switzerland |
| Pierre-Alain | Clavien | University Hospital Zurich | Zurich | Switzerland |
| Ahmad | Al-Haj | University Of Aleppo | Aleppo | Syrian Arab Republic |
| Ahmad | Aljaber | University Of Aleppo | Aleppo | Syrian Arab Republic |
| Ahmad Amir | Kayali | University Of Aleppo | Aleppo | Syrian Arab Republic |
| Lama | Kadoura | University Of Aleppo | Aleppo | Syrian Arab Republic |
| Ezzeldin | Nashed | University Of Aleppo | Aleppo | Syrian Arab Republic |
| Hala | Helaly | University Of Aleppo | Aleppo | Syrian Arab Republic |
| Hasan | Kayali | University Of Aleppo | Aleppo | Syrian Arab Republic |
| Mais | Alhashemi | University Of Aleppo | Aleppo | Syrian Arab Republic |
| Marwa | Aloulou | University Of Aleppo | Aleppo | Syrian Arab Republic |
| Mohammed | Alshaghel | University Of Aleppo | Aleppo | Syrian Arab Republic |
| Nihad | Mahli | University Of Aleppo | Aleppo | Syrian Arab Republic |
| Omar | Al-Abed | University Of Aleppo | Aleppo | Syrian Arab Republic |
| Oula | Azizeh | University Of Aleppo | Aleppo | Syrian Arab Republic |
| Sana | Shaikh Torab | University Of Aleppo | Aleppo | Syrian Arab Republic |
| Wael | Alkhaleel | University Of Aleppo | Aleppo | Syrian Arab Republic |
| Marwan | Al Aliwy | University Of Aleppo | Aleppo | Syrian Arab Republic |
| Omar | Alannaz | University Of Aleppo | Aleppo | Syrian Arab Republic |
| Ahmad | Ghazal | University Of Aleppo, Aleppo University Hospital | Aleppo | Syrian Arab Republic |
| Ruqaya | Masri | University Of Aleppo, Aleppo University Hospital | Aleppo | Syrian Arab Republic |
| Zain | Douba | University Of Aleppo, Aleppo University Hospital | Aleppo | Syrian Arab Republic |
| Ahmed | Saeed Saad | Ain Shams University | Cairo | Syrian Arab Republic |
| Aya | Abdulmonem | Ain Shams University | Cairo | Syrian Arab Republic |
| Mahmoud | Shaban | Ain Shams University | Cairo | Syrian Arab Republic |
| Ahmad | Nabil Alhouri | Al Assad University Hospital | Damascus | Syrian Arab Republic |
| Ahmad | Alhouri | Al Assad University Hospital | Damascus | Syrian Arab Republic |
| Alnour | Soliman | Al Assad University Hospital | Damascus | Syrian Arab Republic |
| Hasan | Nabil Al Houri | Al Assad University Hospital | Damascus | Syrian Arab Republic |
| Hasan | Al Houri | Al Assad University Hospital | Damascus | Syrian Arab Republic |
| Sarah | Omran | Al-Mouwasat Hospital | Damascus | Syrian Arab Republic |
| Aram | Abbas | Al-Mouwasat University Hospital | Damascus | Syrian Arab Republic |
| Majd | Chaaban | Al-Mouwasat University Hospital | Damascus | Syrian Arab Republic |
| Mhd Adib | Al Kudmani | Al-Mouwasat University Hospital | Damascus | Syrian Arab Republic |
| Mohammad Karam | Chaaban | Al-Mouwasat University Hospital | Damascus | Syrian Arab Republic |
| Riad | Alhmaidi | Almujtahed Hospital | Damascus | Syrian Arab Republic |
| Amal | Yousef | Damascus University | Damascus | Syrian Arab Republic |
| Amal | Youssef | Damascus University | Damascus | Syrian Arab Republic |
| Muhammad | Nasri | Damascus University | Damascus | Syrian Arab Republic |
| Hossain | Alkhateb | Tishreen University Hospital | Latakia | Syrian Arab Republic |
| Abdulrahman | Almjersah | Tishreen University Hospital | Latakia | Syrian Arab Republic |
| Naya | Hassan | Tishreen University Hospital | Latakia | Syrian Arab Republic |
| Ahmed | Moussa | Tishreen University Hospital | Lattakia | Syrian Arab Republic |
| Alaa | Hamdan | Tishreen University Hospital | Lattakia | Syrian Arab Republic |
| Ali | Hammed | Tishreen University Hospital | Lattakia | Syrian Arab Republic |
| Ali | Alloush | Tishreen University Hospital | Lattakia | Syrian Arab Republic |
| Bashar | Haj Hassan | Tishreen University Hospital | Lattakia | Syrian Arab Republic |
| Hala | Issa | Tishreen University Hospital | Lattakia | Syrian Arab Republic |
| Hiba | Talal Dahhan | Tishreen University Hospital | Lattakia | Syrian Arab Republic |
| Mahmoud | Souliman | Tishreen University Hospital | Lattakia | Syrian Arab Republic |
| Salah | Hammed | Tishreen University Hospital | Lattakia | Syrian Arab Republic |
| Tharaa | Mehdi Tobba | Tishreen University Hospital | Lattakia | Syrian Arab Republic |
| Alaa | Hamdan | Tishreen University | Tartus | Syrian Arab Republic |
| Seba | Ayoub | Tishreen University | Tartus | Syrian Arab Republic |
| Ming-Chin | Yu | New Taipei Municipal Tucheng Hospital, Chang Gung Medical Foundation | New Taipei City | Taiwan |
| Po-Chih | Yang | Fu Jen Catholic University Hospital | New Taipei City | Taiwan |
| Chien Hui | Wu | National Taiwan University Hospital | Taipei | Taiwan |
| Hanen | Bouaziz | Salah Azaiez Institut | Ariana | Tunisia |
| Khaled | Rahal | Salah Azaiez Institut | Ariana | Tunisia |
| Skander | Slim | Salah Azaiez Institut | Ariana | Tunisia |
| Ayed | Karim | Hopital Habib Bouguetfa De Bizerte. University Tunis El Labar | Bizerte | Tunisia |
| Oussama | Baraket | Hopital Habib Bouguetfa De Bizerte. University Tunis El Labar | Bizerte | Tunisia |
| Ali | Kchaou | Habib Bourguiba University Hospital | Sfax | Tunisia |
| Ammar | Houssem | Sahloul Hospital | Sousse | Tunisia |
| Mohamed | Amine Said | Sahloul Hospital | Sousse | Tunisia |
| Mohamed | Ben Mabrouk | Sahloul Hospital |  |  |
| Karim | Ben Hamida | Institut Salah Azaiez | Tunis | Tunisia |
| Montassar | Ghalleb | Institut Salah Azaiez | Tunis | Tunisia |
| Ahmed | Ben Mahmoud | La Rabta Hospital | Tunis | Tunisia |
| Houcine | Maghrebi | La Rabta Hospital | Tunis | Tunisia |
| Montasser | Jameleddine Kacem | La Rabta Hospital | Tunis | Tunisia |
| Mesut | Tez | Ankara Numune Hospital | Ankara | Turkey |
| N. | Eminesariipek | Ankara University Faculty Of Medicine | Ankara | Turkey |
| Özhan | Çetiindağ | Ankara University Faculty Of Medicine | Ankara | Turkey |
| Acar | Tüzüner | Ankara University Medical School | Ankara | Turkey |
| Kaan | Karayalçın | Ankara University Medical School | Ankara | Turkey |
| Ahmet Cihangir | Emral | Gazi University | Ankara | Turkey |
| Kursat | Dikmen | Gazi University | Ankara | Turkey |
| Mustafa | Kerem | Gazi University | Ankara | Turkey |
| Hüseyin | Bayhan | Gazi University Faculty Of Medicine, Department Of General Surgery | Ankara | Turkey |
| Mehmet | Akif Türkoğlu | Gazi University Faculty Of Medicine, Department Of General Surgery | Ankara | Turkey |
| Nidal | Iflazoğlu | Bursa City Hospital | Bursa | Turkey |
| Ahmet | Özet | Gazi Üniverstesi Tıp Fakültesi | Çankaya | Turkey |
| Ulaş | Aday | Dicle University School Of Medicine | Diyarbakır | Turkey |
| Özcem | Öfkeli | Gazi Yasargil | Diyarbakır | Turkey |
| Alpen | Gumusoglu | Bakırkoy Dr. Sadi Konuk Research And Training Hospital | Istanbul | Turkey |
| Hamit | Ahmet Kabuli | Bakırkoy Dr. Sadi Konuk Research And Training Hospital | Istanbul | Turkey |
| Mehmet | Karabulut | Bakırkoy Dr. Sadi Konuk Research And Training Hospital | Istanbul | Turkey |
| Kivanc | Peker | Basaksehir Cam And Sakura City Hospital | Istanbul | Turkey |
| Sezer | Saglam | Demiroglu Bilim University | Istanbul | Turkey |
| Fatema | Sayed İsmail Rahimi | Istanbul Faculty Of Medicine (Çapa) | Istanbul | Turkey |
| Fatema | Hanefa | Istanbul Faculty Of Medicine (Çapa) | Istanbul | Turkey |
| Arda | Isik | Istanbul Medeniyet University | Istanbul | Turkey |
| Ertugrul | Goksoy | Istanbul University-Cerrahpasa | Istanbul | Turkey |
| Ender | Dulundu | Istanbul University-Cerrahpasa, Cerrahpasa Medical Faculty | Istanbul | Turkey |
| Ali Emre | Atici | Marmara University | Istanbul | Turkey |
| Aysegul | Bahar Ozocak | Marmara University | Istanbul | Turkey |
| Cumhur | Yegen | Marmara University | Istanbul | Turkey |
| Ahmet Cem | Dural | University Of Health Sciences Bakirkoy Dr. Sadi Konuk Training And Research Hospital | Istanbul | Turkey |
| Nuri Alper | Sahbaz | University Of Health Sciences Bakirkoy Dr. Sadi Konuk Training And Research Hospital | Istanbul | Turkey |
| Hanife Seyda | Ulgur | University Of Health Sciences, Umraniye Education And Research Hospital | Istanbul | Turkey |
| Husnu | Aydin | University Of Health Sciences, Umraniye Education And Research Hospital | Istanbul | Turkey |
| Omer Faruk | Ozkan | University Of Health Sciences, Umraniye Education And Research Hospital | Istanbul | Turkey |
| Ozgul | Duzgun | University Of Health Sciences, Umraniye Education And Research Hospital | Istanbul | Turkey |
| Muhammet | Çelik | Yedikule Surp Pırgic Armenian Hospital | Istanbul | Turkey |
| Salih | Pekmezci | Yedikule Surp Pırgic Armenian Hospital | Istanbul | Turkey |
| Ahmet | Çoker | Ege University - Izmir Hpb Clinic | Izmir | Turkey |
| Alper | Uguz | Ege University - Izmir Hpb Clinic | Izmir | Turkey |
| Omer | Vedat Unalp | Ege University - Izmir Hpb Clinic | Izmir | Turkey |
| Ismail | Sert | Egepol Surgery Hospital | Izmir | Turkey |
| Suleyman | Ertekin | Egepol Surgery Hospital | Izmir | Turkey |
| Mucahit | Ozbilgin | Izmir Hpb Clinic | Izmir | Turkey |
| Serdar | Aydoğan | Izmır Tepecık Educatıon Abd Tranıng Hospital | Izmir | Turkey |
| Enver | Tekin | Sbü Izmir Dr Suat Seren Göğüs Hastaliklari Ve Cerrahisi Eah | Izmir | Turkey |
| Bulent | Calik | University Of Health Sciences Tepecik Training And Research Hospital | Izmir | Turkey |
| Degercan | Yesilyurt | University Of Health Sciences Tepecik Training And Research Hospital | Izmir | Turkey |
| Semra | Demirli Atici | University Of Health Sciences Tepecik Training And Research Hospital | Izmir | Turkey |
| Türkmen | Bahadır Arıkan | Ercıyes Unıversty | Kayseri | Turkey |
| Turkmen | Arıkan | Ercıyes Unıversty | Kayseri | Turkey |
| Emre | Gonullu | Sakarya Universitesi Egitim Arastirma Hastanesi | Sakarya | Turkey |
| Enis | Dikicier | Sakarya University Faculty Of Medicine | Sakarya | Turkey |
| Recayi | Capoglu | Sakarya University Faculty Of Medicine | Sakarya | Turkey |
| Zulfu | Bayhan | Sakarya University Faculty Of Medicine | Sakarya | Turkey |
| Sarah | Alfurais | Ondokuz Mayıs University | Samsun | Turkey |
| Elif | Colak | Samsun Training And Research Hospital | Samsun | Turkey |
| Suleyman | Polat | Samsun Training And Research Hospital | Samsun | Turkey |
| Ahmet Burak | Çiftci | University Of Health Sciences Turkey | Samsun | Turkey |
| James | Milburn | Nhs Grampian | Aberdeen | UK |
| Claire | Jones | Belfast Health And Social Care Trust | Belfast | UK |
| David | Vass | Belfast Health And Social Care Trust | Belfast | UK |
| Mark | Taylor | Belfast Health And Social Care Trust | Belfast | UK |
| Bobby VM | Dasari | Queen Elizabeth Hospital | Birmingham | UK |
| Ambareen | Kausar | East Lancashire Nhs Trust | Blackburn | UK |
| Asma | Sultana | East Lancashire Teaching Hospitals Nhs Trust | Blackburn | UK |
| Daren | Subar | East Lancashire Teaching Hospitals Nhs Trust | Blackburn | UK |
| Quentin | Nunes | East Lancashire Teaching Hospitals Nhs Trust | Blackburn | UK |
| James | Skipworth | University Hospitals Bristol and Weston NHS Foundation Trust | Bristol | UK |
| Obi | Nwogwugwu | University Hospitals Bristol and Weston NHS Foundation Trust | Bristol | UK |
| Stijn | van Laarhoven | University Hospitals Bristol and Weston NHS Foundation Trust | Bristol | UK |
| Amar | Kourdouli | Chesterfield Royal Hospital | Chesterfield | UK |
| Altaf Awan | Awan | University Hospitals Of Derby & Burton | Derby | UK |
| Imran | Bhatti | University Hospitals Of Derby & Burton | Derby | UK |
| Javed | Latif | University Hospitals Of Derby & Burton | Derby | UK |
| Fiona | Hand | Royal Infirmary Of Edinburgh | Edinburgh | UK |
| Francis | Robertson | Royal Infirmary Of Edinburgh | Edinburgh | UK |
| David | Holroyd | University Of Glasgow | Glasgow | UK |
| David | Holroyd | University Of Glasgow | Glasgow | UK |
| Nigel | Jamieson | University Of Glasgow | Glasgow | UK |
| William | Lim | University Of Glasgow | Glasgow | UK |
| David | Chang | University Of Glasgow / Glasgow Royal Infirmary | Glasgow | UK |
| Adam | Frampton | Royal Surrey County Hospital | Guildford | UK |
| Rajiv | Lahiri | Royal Surrey County Hospital | Guildford | UK |
| Saurav | Chakravartty | Royal Surrey County Hospital | Guildford | UK |
| Harris | Siddique | Northwick Park Hospital | Harrow | UK |
| Manahil | Bashir | Northwick Park Hospital | Harrow | UK |
| Stephen | Mcnally | Nhs Highland | Inverness | UK |
| Alistair | Young | Leeds Teaching Hospitals Nhs Trust | Leeds | UK |
| Andrew | Smith | Leeds Teaching Hospitals Nhs Trust | Leeds | UK |
| James | Pine | Leeds Teaching Hospitals Nhs Trust | Leeds | UK |
| Giuseppe | Garcea | University Hospitals Of Leicester | Leicester | UK |
| Jonathan | Haqq | University Hospitals Of Leicester | Leicester | UK |
| Deep | Malde | University Hospitals Of Leicester Nhs Trust | Leicester | UK |
| Declan | Dunne | Liverpool University Hospitals | Liverpool | UK |
| Isobel | Burridge | Liverpool University Hospitals | Liverpool | UK |
| Peter | Szatmary | Liverpool University Hospitals | Liverpool | UK |
| Deepak | Hariharan | Barts Health Nhs Trust | London | UK |
| Hemant | Kocher | Barts Health Nhs Trust | London | UK |
| Vincent | Yip | Barts Health Nhs Trust | London | UK |
| Amjad | Khalil | HCA | London | UK |
| Ashitha | Mohandas Nair | Kings College Hospital | London | UK |
| Irene | Liova | Pancreasgroup.Org | London | UK |
| Aleem | O'Balogun | Royal Free London NHS Foundation Trust | London | UK |
| Alex | Rothnie | Royal Free London NHS Foundation Trust | London | UK |
| Bhargava | Chikkala | Royal Free London NHS Foundation Trust | London | UK |
| Camila | Hidalgo Salinas | Royal Free London NHS Foundation Trust | London | UK |
| Carlo | Frola | Royal Free London NHS Foundation Trust | London | UK |
| Charalampos | Tsakiris | Royal Free London NHS Foundation Trust | London | UK |
| Dimitri | Raptis | Royal Free London NHS Foundation Trust | London | UK |
| Dimitrios | Chasiotis | Royal Free London NHS Foundation Trust | London | UK |
| Dinesh | Sharma | Royal Free London NHS Foundation Trust | London | UK |
| Fatema | Jessa | Royal Free London NHS Foundation Trust | London | UK |
| Fiammetta | Soggiu | Royal Free London NHS Foundation Trust | London | UK |
| Giuseppe | Fusai | Royal Free London NHS Foundation Trust | London | UK |
| Ioannis | Kostakis | Royal Free London NHS Foundation Trust | London | UK |
| Manikandan | Kathirvel | Royal Free London NHS Foundation Trust | London | UK |
| Mohamed | Elnagar | Royal Free London NHS Foundation Trust | London | UK |
| Nikolaos | Dimitrokallis | Royal Free London NHS Foundation Trust | London | UK |
| Satheesh | Iype | Royal Free London NHS Foundation Trust | London | UK |
| Stephanos | Pericleous | Royal Free London NHS Foundation Trust | London | UK |
| Ahmed | Mohamed | Royal Free London NHS Foundation Trust | London | UK |
| Alejandro | Ramirez-Del Val | Royal Free London NHS Foundation Trust | London | UK |
| Pascale | Tinguely | Royal Free London NHS Foundation Trust | London | UK |
| Marina | Likos-Corbett | Royal Marsden Hospital | London | UK |
| Iqraa | Afzal | The London Clinic | London | UK |
| Ricky | Bhogal | The Royal Marsden Hospital | London | UK |
| Krishnakumure | Patel | University Of Nottingham | London | UK |
| Ajith K. | Siriwardena | Manchester Royal Infirmary, University Of Manchester | Manchester | UK |
| Nicola | de' Liguori Carino | Manchester Royal Infirmary, University Of Manchester | Manchester | UK |
| Professor | Aali Sheen | Manchester Royal Infirmary, University Of Manchester | Manchester | UK |
| Fahed | Gareb | Queen Elizabith Queen Mother Hospital | Margate | UK |
| Khaled | Ammar | Freeman Hospital | Newcastle | UK |
| Rohan | Thakkar | Freeman Hospital | Newcastle | UK |
| Sanjay | Pandanaboyana | Freeman Hospital | Newcastle | UK |
| John | Leeds | Freeman Hospital | Newcastle | UK |
| Dhanny | Gomez | Nottingham University Hospitals | Nottingham | UK |
| Gordon | Gregory | Nottingham University Hospitals | Nottingham | UK |
| Carlo | Ceresa | Oxford University Hospitals NhS Foundation Trust | Oxford | UK |
| Hussain | Abbas | Oxford University Hospitals NhS Foundation Trust | Oxford | UK |
| Lucia | Lazzereschi | Oxford University Hospitals NhS Foundation Trust | Oxford | UK |
| Srikanth | Reddy | Oxford University Hospitals NhS Foundation Trust | Oxford | UK |
| Alex | Gordon-Weeks | Oxford University Hospitals NhS Foundation Trust | Oxford | UK |
| Somaiah | Aroori | University Hospitals Plymouth Nhs Trust | Plymouth | UK |
| Thomas | Russell | University Hospitals Plymouth Nhs Trust | Plymouth | UK |
| Keith | Roberts | Uhb | Redditch | UK |
| Nikolaos | Chatzizacharias | Uhb | Redditch | UK |
| Robert | Sutcliffe | Uhb | Redditch | UK |
| Bilal | Al-Sarireh | Morriston Hospital | Swansea | UK |
| Guy | Shingler | Morriston Hospital | Swansea | UK |
| Matt | Mortimer | Morriston Hospital | Swansea | UK |
| Denys | Skoryi | Kharkiv Regional Center Of Oncology | Kharkiv | Ukraine |
| Ievgen | Ilin | Kharkiv Regional Center Of Oncology | Kharkiv | Ukraine |
| Margaryta | Pisetska | Kharkiv Regional Center Of Oncology | Kharkiv | Ukraine |
| Dmytro | Cheverdiuk | National Cancer Institute | Kiev | Ukraine |
| Kopchak | Kostyantyn | National Cancer Institute | Kyiv | Ukraine |
| Kostiantyn | Kopchak | National Cancer Institute | Kyiv | Ukraine |
| Oleksandr | Kvasivka | National Cancer Institute | Kyiv | Ukraine |
| Sumarokova | Valeriia | National Cancer Institute | Kyiv | Ukraine |
| Valeriia | Sumarokova | National Cancer Institute | Kyiv | Ukraine |
| Vitalii | Kryzhevskyi | National Cancer Institute | Kyiv | Ukraine |
| Sergei | Sikachov | National Cancer Institute | Kyiv | Ukraine |
| Andrii | Khomiak | Shalimov National Institute Of Surgery And Transplantology | Kyiv | Ukraine |
| Andrii | Malik | Shalimov National Institute Of Surgery And Transplantology | Kyiv | Ukraine |
| Igor | Khomiak | Shalimov National Institute Of Surgery And Transplantology | Kyiv | Ukraine |
| Andriy | Bilyak | Lviv Regional Clinical Hospital | Lviv | Ukraine |
| Serge | Chooklin | Lviv Regional Clinical Hospital | Lviv | Ukraine |
| Serhii | Chuklin | Lviv Regional Clinical Hospital | Lviv | Ukraine |
| Iurii | Mikheiev | Zaporizhzhya State Medical University | Zaporizhia | Ukraine |
| Oleh | Shylenko | Zaporizhzhya State Medical University | Zaporizhia | Ukraine |
| Andrii | Klymenko | Zaporizhzhya State Medical University | Zaporizhzhya | Ukraine |
| Shirali | Patel | Ascension Saint Agnes Hospital And Cancer Institute | Baltimore | USA |
| Steven | Cunningham | Ascension Saint Agnes Hospital And Cancer Institute | Baltimore | USA |
| Mark | Callery | Beth Israel Deaconess Medical Center | Boston | USA |
| Tara | Kent | Beth Israel Deaconess Medical Center | Boston | USA |
| Chandrajit | Raut | Brigham And Women'S Hospital | Boston | USA |
| Jiping | Wang | Brigham And Women'S Hospital | Boston | USA |
| Mark | Fairweather | Brigham And Women'S Hospital | Boston | USA |
| Megan | Sulciner | Brigham And Women'S Hospital | Boston | USA |
| Sameer | Hirji | Brigham And Women'S Hospital | Boston | USA |
| Thomas | Clancy | Brigham And Women'S Hospital | Boston | USA |
| Martina | Nebbia | Massachusetts General Hospital | Boston | USA |
| Motaz | Qadan | Massachusetts General Hospital | Boston | USA |
| Amanda | Musser | Premier Health | Centerville | USA |
| Melissa | Hogg | Northshore University Healthsystem | Chicago | USA |
| Jennifer | Rodriquez | Uchealth Memorial Hospital Central | Colorado Springs | USA |
| John | Hamner | Uchealth Memorial Hospital Central | Colorado Springs | USA |
| Liz | Hennessy | Uchealth Memorial Hospital Central | Colorado Springs | USA |
| Aaron | Dinerman | Baylor University Medical Center | Dallas | USA |
| Amar | Gupta | Baylor University Medical Center | Dallas | USA |
| Charles | Kimbrough | Baylor University Medical Center | Dallas | USA |
| Rachel | Thompson | Baylor University Medical Center | Dallas | USA |
| Herbert J. | Zeh | The University Of Texas Southwestern | Dallas | USA |
| Imad | Radi | The University Of Texas Southwestern | Dallas | USA |
| Patricio M. | Polanco | The University Of Texas Southwestern | Dallas | USA |
| Dimitrios | Moris | Duke University Medical Center | Durham | USA |
| Michael E. | Lidsky | Duke University Medical Center | Durham | USA |
| David | Lee | Inova Schar Cancer Institute | Fairfax | USA |
| James | Piper | Inova Schar Cancer Institute | Fairfax | USA |
| Jennifer | Gnerlich | Inova Schar Cancer Institute | Fairfax | USA |
| Daniel | Tuvin | University Of North Dakota, Sanford Health | Fargo | USA |
| Robert | Sticca | University Of North Dakota, Sanford Health | Fargo | USA |
| Sabha | Ganai | University Of North Dakota, Sanford Health | Fargo | USA |
| Niraj | Gusani | Baptist Md Anderson Cancer Center | Jacksonville | USA |
| Derek | Krinock | Uams Medical Center | Little Rock | USA |
| Emmanouil | Giorgakis | Uams Medical Center/ Winthrop P Rockefeller Cancer Institute | Little Rock | USA |
| Hailey | Hardgrave | Uams Medical Center/ Winthrop P Rockefeller Cancer Institute | Little Rock | USA |
| Richard T. | Spencer-Cole | Uams Medical Center/ Winthrop P Rockefeller Cancer Institute | Little Rock | USA |
| Garrett | Klutts | University Of Arkansas For Medical Sciences | Little Rock | USA |
| Hailey | Hardgrave | University Of Arkansas For Medical Sciences | Little Rock | USA |
| Joe | Nigh | University Of Arkansas For Medical Sciences | Little Rock | USA |
| Joseph | Nigh | University Of Arkansas For Medical Sciences | Little Rock | USA |
| Juan Camilo | Barreto Andrade | University Of Arkansas For Medical Sciences | Little Rock | USA |
| Michail | Mavros | University Of Arkansas For Medical Sciences | Little Rock | USA |
| Tamara | Osborn | University Of Arkansas For Medical Sciences | Little Rock | USA |
| Cristina | Ferrone | Cedars-Sinai Medical Center | Los Angeles | USA |
| Victoria | O'Connor | Kaiser Permanente Medical Group | Los Angeles | USA |
| Brian | Boone | West Virginia University | Morgantown | USA |
| Britney | Harris | West Virginia University | Morgantown | USA |
| Carl | Schmidt | West Virginia University | Morgantown | USA |
| Beth | Schrope | Columbia University | New York | USA |
| John | Chabot | Columbia University | New York | USA |
| Michael | Kluger | Columbia University | New York | USA |
| Erika | Tay Lasso | University Of California Irvine Medical Center | Orange | USA |
| Avinoam | Nevler | Thomas Jefferson University Hospital | Philadelphia | USA |
| Charles | Yeo | Thomas Jefferson University Hospital | Philadelphia | USA |
| Francesca | Ponzini | Thomas Jefferson University Hospital | Philadelphia | USA |
| Harish | Lavu | Thomas Jefferson University Hospital | Philadelphia | USA |
| Ryan | Lamm | Thomas Jefferson University Hospital | Philadelphia | USA |
| Wilbur | Bowne | Thomas Jefferson University Hospital | Philadelphia | USA |
| Nina | Kyser | Zucker Hillside Hospital | Queens | USA |
| Christos | Galanopoulos | Renown Health | Reno | USA |
| Arezou | Abbasi | University Of Washington | Seattle | USA |
| James | Park | University Of Washington | Seattle | USA |
| Jonathan | Sham | University Of Washington | Seattle | USA |
| Lindsay | Dickerson | University Of Washington | Seattle | USA |
| Venu | Pillarisetty | University Of Washington | Seattle | USA |
| Iswanto | Sucandy | Adventhealth Tampa | Tampa | USA |
| Sharona | Ross | Adventhealth Tampa | Tampa | USA |
| Emily | Winslow | Medstar Georgetown University Hospital | Washington | USA |
| Jasn | Hawksworth | Medstar Georgetown University Hospital | Washington | USA |
| Pejman | Radkani | Medstar Georgetown University Hospital | Washington | USA |
| Thomas | Fishbein | Medstar Georgetown University Hospital | Washington | USA |
| Armando | Salim Munoz | University Of Massachusetts | Worcester | USA |
| James | Lindberg | University Of Massachusetts | Worcester | USA |
| Paulo N. | Martins | University Of Massachusetts | Worcester | USA |
| Rafat | Ameen Mohammed Al-saban | . | Sana'a | Yemen |
| Rafat | Al-Saban | . | Sana'a | Yemen |
| Waheeb | Al-Kubati | 21 September University | Sana'a | Yemen |
| Asma | Ali Ahmed Ghallab | Al-Thawra Modern General Hospital | Sana'a | Yemen |
| Ghadeer | Mohammed Alsanany | Al-Thawra Modern General Hospital | Sana'a | Yemen |
| Hassan | Almarashi | Al-Thawra Modern General Hospital | Sana'a | Yemen |
| Hytham | Al_Samawi | Al-Thawra Modern General Hospital | Sana'a | Yemen |
| Mohammed | Abdulkhaleq Mohammed Mohsen Al-Asadi | Al-Thawra Modern General Hospital | Sana'a | Yemen |
| Ramzi | Alsayadi | Al-Thawra Modern General Hospital | Sana'a | Yemen |
| Sara | Hail | Al-Thawra Modern General Hospital | Sana'a | Yemen |
| Sarah | Shream | Al-Thawra Modern General Hospital | Sana'a | Yemen |
| Hadeel | Mohmmed Bajjah | Al-Thawra Modern General Hospital | Sana'a | Yemen |
| Saba | Al - Ameri | Al-Thawra Modern General Hospital | Sana'a | Yemen |
| Hadeel | Bajjah | Al-Thawra Modern General Hospital | Sana'a | Yemen |
| Saba | Ahmed Ahmed Saleh Al-Ameri | Al-Thawra Modern General Hospital | Sana'a | Yemen |
| Nagra | Are Al-Dowsh | Alkuwait University Hospital | Sana'a | Yemen |
| Nagran | Aref AlDowsh | Alkuwait University Hospital | Sana'a | Yemen |
| Qaeid | Al-Khawlani | Alkuwait University Hospital | Sana'a | Yemen |
| Yahya | Ali Ali Murshed | Alkuwait University Hospital | Sana'a | Yemen |
| Mohammed | Al-Shehari | Sana'a University, Al-Thawra Modern General Hospital | Sana'a | Yemen |
| Amir Al-Deen | Jahaf | Althowra Hospital | Sana'a | Yemen |
| Ebrahim | Ahmed Esmail Al-sharabi | Althowra Hospital | Sana'a | Yemen |
| Hamdan | Aldumaini | Althowra Hospital | Sana'a | Yemen |
| Zainab | Alattas | Althowra Hospital | Sana'a | Yemen |
| Ali | Almassaudi | Althowrah Modern General Hospital ,21St September'S University | Sana'a | Yemen |
| Hadeel | Muhammed Ahmed Hussein Bajjah | Althowrah Modern General Hospital ,21St September'S University | Sana'a | Yemen |
| Rudaina | Albakry | Dar Al-Salam International University For Science & Technology | Sana'a | Yemen |
| Hamza | Al-Naggar | Faculty Of Medicine - Sana'A University | Sana'a | Yemen |
| Sarah | Abdulkhaliq Ali Shream | Faculty Of Medicine - Sana'A University | Sana'a | Yemen |
| Anter | Al Affary | Faculty Of Medicine And Health Sciences - Sana'A University | Sana'a | Yemen |
| Eman | Al-Markiz | Faculty Of Medicine And Health Sciences - Sana'A University | Sana'a | Yemen |
| Fatima | Al-Eryani | Faculty Of Medicine And Health Sciences - Sana'A University | Sana'a | Yemen |
| Heba | Farhat | Faculty Of Medicine And Health Sciences - Sana'A University | Sana'a | Yemen |
| Qannaf | Al Qadasi | Faculty Of Medicine And Health Sciences - Sana'A University | Sana'a | Yemen |
| Khaled | Alwafy | Sana | Sana'a | Yemen |
| Mahmmoud | Yehia Mohammed Abdualqader | Sana | Sana'a | Yemen |
| Ramzi | Ali Abdullah Yahya Ali | Sana | Sana'a | Yemen |
| Aisha | Albar | Sana'A University | Sana'a | Yemen |
| Hikma | Abdullh Bleem | Sana'A University | Sana'a | Yemen |
| Khaled | Sultan Ali Galeb | Sana'A University | Sana'a | Yemen |
| Mohammed | Ghushaim | Sana'A University | Sana'a | Yemen |
| Mohammed | Sabbar | Sana'A University | Sana'a | Yemen |
| Muhib | Esmail | Sana'A University | Sana'a | Yemen |
| Ramzi | Abdullah Yahya Ali | Sana'A University | Sana'a | Yemen |
| Rana | Hassan Mohammed Salem | Sana'A University | Sana'a | Yemen |
| Rana | Salem | Sana'A University | Sana'a | Yemen |
| Wail | Saif | Sana'A University | Sana'a | Yemen |
| Siham | Al-Faiq | Sana'A University Faculty of Medical Health And Science | Sana'a | Yemen |
| Ebrahim | Alsharabi | Sana'A University-Althawra Hospital | Sana'a | Yemen |
| Almekhlafi | Tofik Abdul Hameed | Sana’A Universty | Sana'a | Yemen |
| Tofik | Almekhlafi | Sana’A Universty | Sana'a | Yemen |
| Abdulrahman | Omairan | Sanaa | Sana'a | Yemen |
| Eman | Almarkiz | Sanaa | Sana'a | Yemen |
| Heba | Abduljawad | Sanaa | Sana'a | Yemen |
| Omair | Mansaleh | Sanaa University | Sana'a | Yemen |
| Watheeq | Al-Melhani | Sanaa University - Faculity Of Medicine | Sana'a | Yemen |
| Mahmmoud | Abdualqader | Sanna'A University | Sana'a | Yemen |
| Radfan | Al-Abdi | University Of Science And Technology Hospital | Sana'a | Yemen |
| Hussein Mohammed | Alwan | University Sana'A | Taiz | Yemen |
| Chenesa | Mbanje | University Of Zimbabwe | Harare | Zimbabwe |
| Onesai | Chihaka | University Of Zimbabwe | Harare | Zimbabwe |
